# Unleashing the Potential of Raman Spectroscopy to Estimate PEDOT:PSS Doping Level and Crystalline Morphology

**DOI:** 10.1002/advs.202513726

**Published:** 2025-12-05

**Authors:** Tzu‐Yi Thomas Yu, Tsu‐Yu Chou, Viktor Naenen, Bokai Zhang, Hasan Emre Baysal, Bora Bulut, Martin Rosenthal, Francisco Molina‐Lopez

**Affiliations:** ^1^ Department of Materials Engineering (MTM) KU Leuven Leuven 3001 Belgium; ^2^ Department of Chemistry KU Leuven Leuven 3001 Belgium; ^3^ European Synchrotron Radiation Facility (ESRF) Grenoble 38000 France

**Keywords:** charge transport, PEDOT doping level, PEDOT:PSS crystalline morphology, Raman microscopy

## Abstract

PEDOT:PSS is arguably the most popular conjugated polymer. However, its complex blend nature and low crystallinity make its characterization challenging. Raman spectroscopy is a fast and accessible technique for studying PEDOT:PSS. However, PEDOT:PSS Raman signals are convoluted, and their current interpretation, focused on the C_α_═C_β_ peak (≈1435 cm^−1^), cannot distinguish between doping and morphology information. The interpretation of PEDOT:PSS Raman spectra is advanced by expanding the research to other vibrational peaks. By comparing the Raman spectra of different PEDOT:PSS samples with GIWAX and XPS data, two equations are proposed to estimate the PEDOT π–π intermolecular spacing and doping levels from Raman data. The estimated PEDOT doping levels, which are confirmed with the corresponding equilibrium equation, allowed to calculate the PEDOT:PSS films' carrier mobility. Notably, the calculated mobilities agree with the transport coefficient fitted from the Kang–Snyder model. The high spatial resolution of Raman is exploited to realize a morphology and doping level 2D mapping on blank films that are locally treated by inkjet printing with 100 µm resolution. This study re‐establishes the interpretation of PEDOT:PSS Raman spectra, demonstrates its potential for quantitative analysis of crystalline parameters, doping, and mobility, and opens the door to the local probing of those properties.

## Graphical abstract

Herein, the correlational changes of each PEDOT Raman peak to morphology and doping level are identified. Two equations are derived to estimate PEDOT π–π spacing and doping level from Raman spectra (from which mobility is also extracted). GIWAXS and XPS results validate the estimation equations. The potential of Raman to probe locally PEDOT properties with good resolution is further demonstrated.

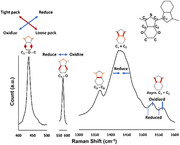

## 1 Introduction

Poly(3,4‐ethylenedioxythiophene):poly(styrenesulfonate) (PEDOT:PSS) is a popular material used in organic electronics due to its exceptionally high electrical conductivity, ambient stability, tunable electrical properties, and excellent processability. These advantages have enabled PEDOT:PSS to be employed in a broad range of device applications, including transparent electrodes,^[^ ^1^ ^]^ interconnects,^[^ ^2^, ^3^ ^]^ the active materials in organic electrochemical transistors (OECTs),^[^ ^4^, ^5^, ^6^ ^]^ components of organic thermoelectric generators (OTEs),^[^ ^7^ ^]^ and other various electronic technologies.^[^ ^8^, ^9^, ^10^, ^11^, ^12^ ^]^ Furthermore, because of its biocompatibility, PEDOT:PSS is also used in biosensor applications.^[^ ^13^, ^14^ ^]^ Despite the prevalence of PEDOT:PSS in organic electronics, it remains challenging to study the microstructure and doping level of PEDOT:PSS in its device configurations, especially for those applications that alter PEDOT:PSS properties during device operation.^[^ ^9^, ^10^, ^11^ ^]^ For microstructure, differential scanning calorimetry (DSC) shows no clear crystallization or melting peaks for PEDOT:PSS before the decomposition temperature because of its low crystallinity from the polymer blend nature.^[^ ^15^, ^16^ ^]^ Wide‐angle X‐ray scattering (WAXS) reveals broad diffraction peaks for PEDOT:PSS, but the low crystallinity of PEDOT:PSS requires a source of synchrotron X‐ray and careful signal treatment, posing a significant access barrier to the study of the material's crystallinity. Moreover, the measurable samples are limited to free‐standing bulk materials or single‐layer thin films on flat substrates in grazing‐incidence mode (GIWAXS).^[^ ^17^, ^18^ ^]^ Finally, although local probing with micro‐focused GIWAXS (µGIWAXS) has been reported, the technique remains a niche expertise of highly specialized groups.^[^ ^19^, ^20^, ^21^ ^]^ On the other hand, the measurement of PEDOT:PSS doping level is equally challenging. While Hall Effect measurement is a standard technique to determine charge carrier type and density of inorganic semiconductors/conductors, it is unreliable for organic materials due to charge localization. UV–vis absorption spectroscopy has been demonstrated to quantify the PEDOT doping level, but the reported method is only suitable for lightly doped PEDOT (<20%) on transparent substrates.^[^ ^22^ ^]^ X‐ray photoelectron spectroscopy (XPS) is the only reliable technique for PEDOT doping levels, but it is slow, requires a high‐vacuum environment, and also imposes restrictions on the sample shape.

Raman spectroscopy is an ideal alternative technique to characterize PEDOT:PSS morphology and doping level. It can be applied to most sample forms, including solutions, on any substrate under ambient conditions. It is fast (a few seconds) and allows high lateral spatial resolution (micrometer‐scale).^[^ ^23^ ^]^ Though PEDOT:PSS Raman spectra provide both the morphology and doping level information, they cannot be distinguished with the existing theories.^[^ ^24^ ^]^ The current interpretation of PEDOT:PSS Raman spectra focuses on the peak centered ≈1435 cm^−1^, attributed to the thiophene ring vibration dominated by its C_α_═C_β_ bond.^[^ ^25^, ^26^ ^]^ In the cases of secondary doped PEDOT:PSS, the red shift of C_α_═C_β_ represents PEDOT backbone extension to form a more linear conformation. Ouyang et al. interpreted this shift based on the boosted electrical conductivity after adding a secondary dopant in PEDOT:PSS.^[^ ^27^, ^28^ ^]^ This hypothesis remains widely accepted today. On the other hand, reducing the doping level either chemically or electrochemically also shifts the C_α_═C_β_ peak to a lower wavenumber.^[^ ^29^, ^30^, ^31^ ^]^ With density function theory (DFT) simulation results, Peng et al. showed that the neutral PEDOT exhibits a C_α_═C_β_ peak at a lower wavenumber (1402 cm^−1^) than that of oxidized PEDOT (1417 cm^−1^).^[^ ^31^ ^]^ Therefore, the C_α_═C_β_ peak redshifts toward 1402 cm^−1^ by PEDOT de‐doping as PEDOT is gradually dominated by neutral units. Unfortunately, because both the PEDOT morphology change and doping lead to the C_α_═C_β_ peak shifting, distinguishing between these two effects using PEDOT:PSS Raman spectra alone remains currently challenging, and could lead to misinterpretations of a specific treatment effect on PEDOT. As far as our knowledge goes, none of the reported studies covers both the PEDOT doping level and morphology changes and distinguishes the two factors. This is an important issue because, according to the Drude model, conductivity σ depends on both mobility, *µ*, and charge carrier density, *n*, as σ = *e∙µ∙n* (with *e* being the elemental charge). Thus, it is currently not possible to differentiate from Raman spectroscopy whether conductivity improvements in post‐treated PEDOT:PSS arise from mobility/morphology or doping/charge carrier density changes.

Herein, we present a comprehensive study to separate the signature of PEDOT doping level and morphology changes in PEDOT:PSS Raman spectra. Also, we provide a new method to estimate locally PEDOT intermolecular spacing and doping level with micrometer resolution, which is paramount to studying PEDOT:PSS integrated in microdevices. This study is based on two series of PEDOT:PSS thin film samples. The first series, denoted as the polar‐solvent post‐treated (PSPT) samples, was post‐treated with solvents with various polarities to induce different extents of morphological change without altering the doping level. The second series, labeled as the Na_2_SO_3_‐reduced samples, was post‐treated with sodium sulfate (Na_2_SO_3_) in various concentrations to modify the PEDOT doping level without significantly impacting the morphology. We identify Raman features able to distinguish the PEDOT morphology changes and doping level by comparing the Raman spectra of the two sample series. By correlating PEDOT:PSS Raman spectra with the PEDOT crystalline structure extracted from GIWAXS and the doping level extracted from XPS, we propose equations to simultaneously estimate PEDOT intermolecular distance (π‐π stacking distance) and doping level from Raman spectra. The estimated PEDOT doping level allows calculating the charge carrier density and mobility of PEDOT:PSS films. The charge carrier density agrees with the equilibrium equation, and the mobility correlates well with the transport coefficient, which is used as a proxy for mobility in the popular Kang‐Snyder charge transport model,^[^ ^32^ ^]^ underpinning the validity of our approach. The mapping capability of Raman was demonstrated by performing a 2D mapping of the π–π stacking distance and doping level of a blank PEDOT:PSS film treated locally by inkjet printing with 100 µm resolution.

## 2 Results

**Figure 1:**
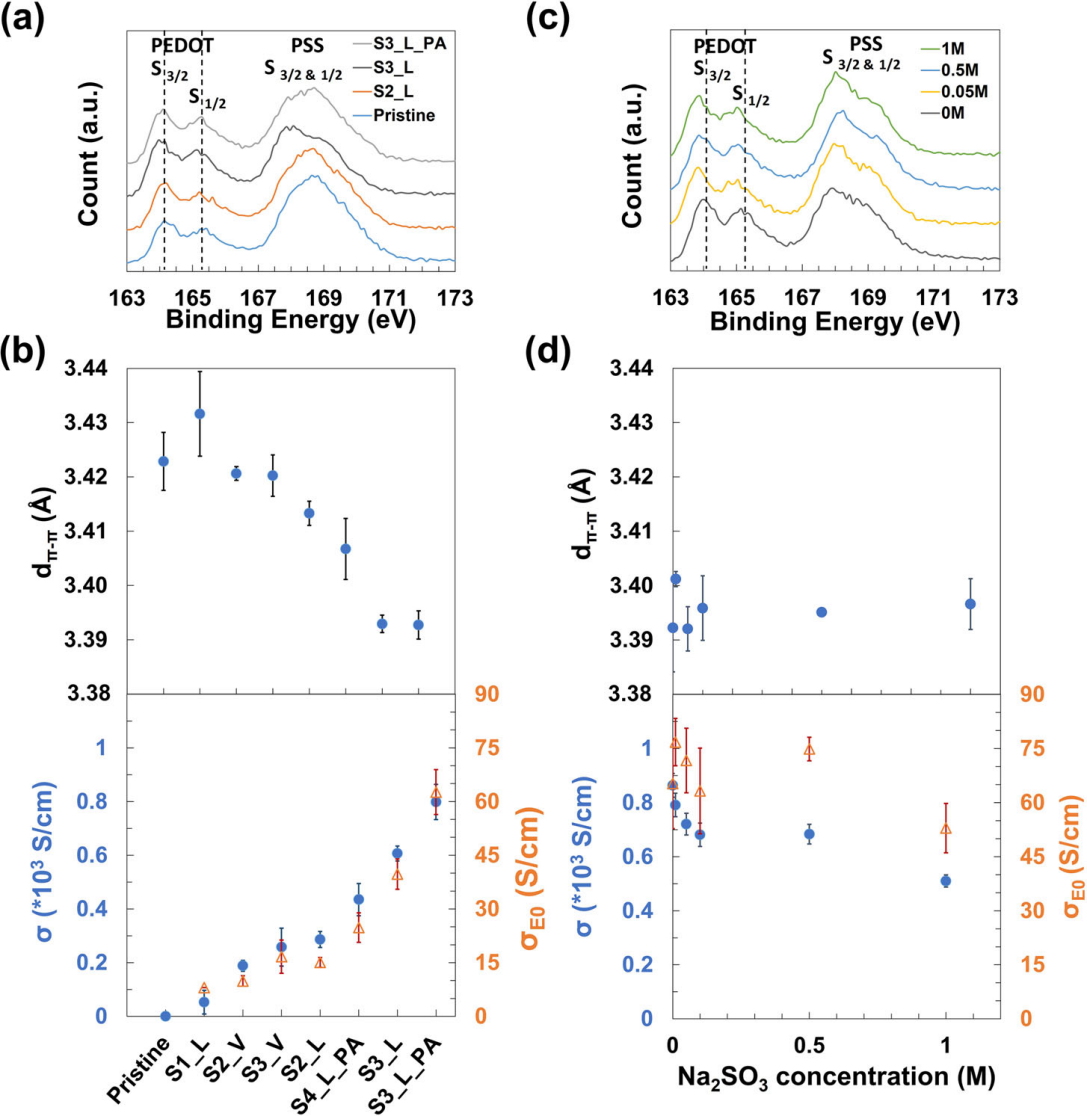
a) XPS S_2p_ spectra of pristine, S2_L, S3_L, and S3_L_PA samples (sample population n = 1). b) (Top) The PEDOT π–π spacing of PSPT thin films in order of increasing conductivity value (n = 3); (Bottom) the electrical conductivity, σ, and transport coefficient, σ_E0_, of PSPT thin films arranged in increasing order (n = 3). c) XPS S_2p_ spectra of PEDOT:PSS samples reduced by 0, 0.05, 0.5, and 1 M Na_2_SO_3_ solution, respectively (n = 1). d) (Top) The π–π spacing of Na_2_SO_3_‐reduced thin films (n = 3); (Bottom) the electrical conductivity σ and transport coefficient σ_E0_ of Na_2_SO_3_‐reduced thin films (n = 3). (Values: mean ± standard error).

**Table** **1** lists the post‐treatment method and the XPS results of PSPT PEDOT:PSS thin films. According to the literature, post‐treatment with small polar molecules results in different PEDOT:PSS morphologies while leaving the PEDOT doping level unaltered.^[^ ^34^ ^]^ Therefore, we applied various non‐reactive polar solvents post‐treatments on PEDOT:PSS thin films to induce different morphologies and avoid changing their PEDOT doping level. First, we used XPS to test the PEDOT doping levels of selected PSPT samples (**Figure** **1**a). In the XPS spectra, the PEDOT S_3/2_ and S_1/2_ bands (164 and 165.5 eV, respectively) have no significant change in intensity and position upon post‐treatment, confirming that polar solvents have a negligible effect on PEDOT doping level. Although qualitatively, UV–vis–NIR spectroscopy supports the same claim (Figure S1, Supporting Information). The PEDOT doping levels can be quantified by fitting XPS spectra with reported protocols,^[^ ^35^, ^36^ ^]^ and the results yielded ≈38% (shown in Table 1) for all tested PSPT samples. This value is close to the reported doping level of pristine PEDOT:PSS.^[^ ^37^, ^38^ ^]^ Unsurprisingly, the PSS‐to‐PEDOT ratio decreased after the polar solvent removed non‐bound PSS excess, and the higher the solvent polarity, the more effective the non‐bound PSS removal (note that the discrepancy S3_L and S3_L_PA is attributed to experimental error since mild annealing cannot alter the amount of PSS). Nonetheless, the PSS‐to‐PEDOT ratio had no apparent effect on PEDOT doping level.^[^ ^39^, ^40^, ^41^ ^]^ Then, the crystalline morphology of PSPT samples was studied by grazing‐incidence wide‐angle X‐ray scattering (GIWAXS). The 2D GIWAXS patterns and 1D line cuts are shown in Figure S2 (Supporting Information), and the crystalline structure information is shown in Figure 1b (π‐π stacking distance), Figure S3 (Supporting Information) (coherence length and paracrystallinity), and Table S1 (Supporting Information). The results show that, in general, polar solvent post‐treatment enhances the crystalline order by reducing the PEDOT intermolecular packing distance (d_π‐π_) in the π–π stacking direction (Figure 1b). Furthermore, the d_π‐π_ reduction is more effective for liquid treatment than vapor treatment and, except for sample S1_L (dioxane post‐treatment) with high experimental error and sample S4_L_PA (glycerol post‐treatment), it correlates with the solvent polarity. Similar correlations were observed for the improvement in the macroscopic electrical transport (Figure 1b). The anomalies in the trend observed for S1_L and S4_L_PA could be due to the large molecular size of dioxane and glycerol compared to ethanol and ethylene glycol, which could preclude their efficient penetration into the PSS. The electrical properties of PSPT samples are boosted from less than 1 up to ≈800 S cm^−1^ after polar‐solvent post‐treatment. Furthermore, the transport coefficient (σ_*E*0_), a mobility proxy extracted from the Kang‐Snyder model for thermoelectrics (see Experimental Methods, Supporting Information),^32^ correlates well with the d_π‐π_ reduction and closely follows the conductivity evolution (Figure 1b). Considering the Drude model, the results in Figure 1a–b suggest that the boosted electrical conductivity of PSPT films is mainly contributed by an increase in mobility resulting from the PEDOT microstructure change, rather than by an increase in carrier concentrations due to PEDOT doping.

The XPS results corresponding to Na_2_SO_3_‐reduced PEDOT: PSS thin films are listed in Table 1. After an initial EG dipping and annealing post‐treatment to stabilize morphology, we reduced the PEDOT:PSS doping level by introducing the films into a solution of sodium sulfite (Na_2_SO_3_), a reported mild reductant for PEDOT:PSS.^[^ ^30^, ^42^ ^]^ In the XPS S2p spectra (Figure 1c), while PSS signals (167–171 eV) remain unaltered, the PEDOT S_3/2_ and S_1/2_ peaks (164 and 165.5 eV, respectively) demonstrate substantial shifting to a lower binding energy after Na_2_SO_3_ post‐treatment, a sign of chemical reduction.^[^ ^35^, ^36^ ^]^ According to XPS peak fitting, the PEDOT doping level dropped consistently from 37.6% to 22.2% while the PSS‐to‐PEDOT ratio was not significantly affected after treatment with Na_2_SO_3_ solution (Table 1). The de‐doping of PEDOT: PSS was qualitatively confirmed by UV–vis–NIR spectroscopy (Figure S1b, Supporting Information). On the other hand, the PEDOT crystalline morphology was investigated with GIWAXS in Figure 1d (π‐π stacking distance), Figure S4 (Supporting Information) (GIWAXS 2D patterns), Figure S5 (Supporting Information) (coherence length and paracrystallinity), and Table S1 (Supporting Information). Figure 1d shows that Na_2_SO_3_‐reduction has a limited effect on the PEDOT π–π spacing compared to that observed in Figure 1b for PSPT films. Nevertheless, the de‐doping effect of Na_2_SO_3_ reduction was supported by a monotonic moderate decrease in electrical conductivity, from 860 S cm^−1^ down to 509 S cm^−1^ (Figure 1d). Notably, the maximum electrical conductivity reduction ratio (509/860 = 0.592) was found to be identical to the PEDOT doping level change indicated by XPS in Table 1 (22.2/37.6 = 0.59), implying that carrier mobility was not affected by the reduction reaction. Also, the transport coefficient (σ_*E*0_) from the Kang‐Snyder model fitting (Figure 1d) displayed little (and trendless) variation (compared to the measured error) among Na_2_SO_3_‐reduced samples, indicating that all the samples had similar mobility. Therefore, we can conclude that Na_2_SO_3_ post‐treatment reduced the PEDOT doping level without imparting significant changes in the PEDOT: PSS morphology.

**Table 1:** Information list of polar‐solvent post‐treated (PSPT) and Na_2_SO_3_‐reduced PEDOT:PSS samples. The PSPT samples are arranged in increasing order of solvent polarity, and the Na_2_SO_3_‐reduced samples are arranged in increasing order of Na_2_SO_3_ concentrations.

Sample Name		Post‐Treatment				XPS	
		Solvent (ENT)	Type	PA	Na_2_SO_3_ concen. [M]	PSS:PEDOT Ratio	DL [%]
PSPT	Pristine	–	–	–	–	2.7	37.1
	S1_L	D (0.164)	L	–	–	–	–
	S2_V	EtOH (0.654)	V	–	–	–	–
	S2_L		L	–	–	2.22	39.8
	S3_V	EG (0.790)	V	–	–	–	–
	S3_L		L	–	–	1.56	39.0
	S3_L_PA		L	Y	–	1.86	37.0
	S4_L_PA	Gly (0.815)	L	Y	–	–	–
Na_2_SO_3_‐reduced	0M	EG (0.790)	L	Y	0	1.50	37.6
	0.01M				0.01	–	–
	0.05M				0.05	1.70	29.0
	0.1M				0.1	–	–
	0.5M				0.5	1.63	24.0
	1M				1	1.63	22.2

D = dioxane; EtOH = ethanol; EG = ethylene glycol; Gly = glycerol; ENT = empirical solvent polarity parameter;^[^ ^33^ ^]^

V = vapor post‐treatment;

L = liquid post‐treatment;

PA = post‐thermal annealed at 120 °C for 10 min;

DL = PEDOT doping level, ≡ [EDOT^+^]/([EDOT^+^]+[EDOT^0^]) [%]. Single sample measurement (n = 1) for each condition in XPS measurement. Details of sample preparation are described in Experimental Methods (Supporting Information).

[Correction added on 22 December 2025, after first online publication: A few typographical errors in Tables 1 and 2 have been updated in this version.]

**Figure 2:**
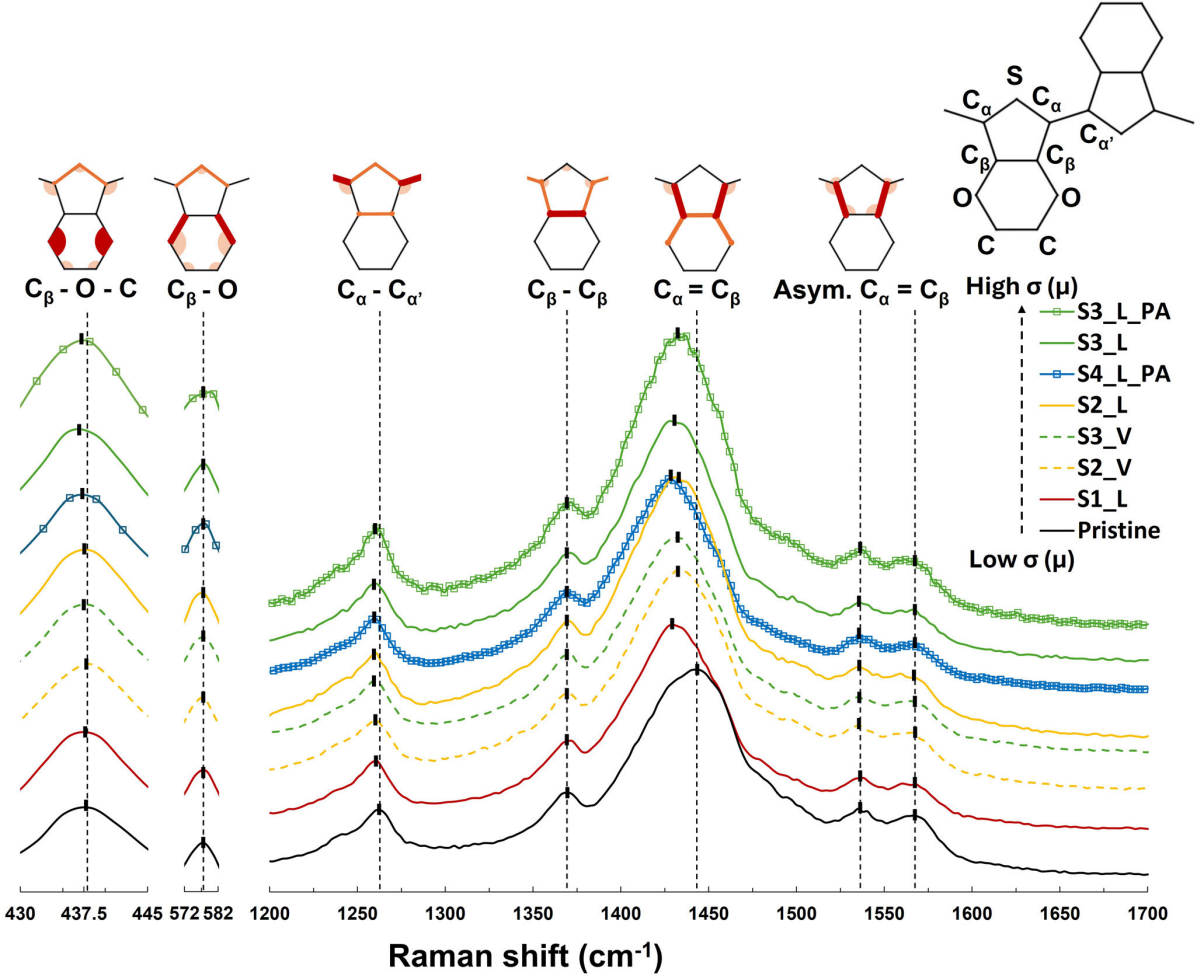
Raman spectra of PSPT PEDOT:PSS thin film samples arranged from less conductive (bottom) to more conductive (top). Each presented spectrum is the average of three replicate samples on silicon substrates (sample population n = 3). All spectra are offset with the reported silicon Raman peak (520 cm^−1^) and normalized by the C_β_–O peak area. The background signal is subtracted. C_β_–O–C (437 cm^−1^) peak slightly shifts toward lower wavenumbers after post‐treatment and correlates well with the conductivity. C_β_–O (577 cm^−1^), C_β_–C_β_ (1361 cm^−1^) and Cα═Cβ (1545 and 1565 cm−1) peaks have no significant shift. C_α_–C_α’_ (1260 cm^−1^) and C_α_═C_β_ (1435 cm^−1^) peaks have significant shifts after polar solvent post‐treatment, but the shifts do not correlate with the conductivity value. The contributing vibrational modes (circular sector: scissoring and rocking; line: stretching) of each peak are illustrated in orange in the EDOT molecular structure. Peaks are named by their most contributing vibrational bonding (highlighted in dark red).^[^ ^25^, ^26^ ^]^ (This figure contains the Raman spectra of the PSPT PEDOT:PSS samples used for GIWAXS measurements).

After characterizing the doping and crystalline properties of the two sample series, we can link them to their corresponding Raman spectra. All the spectra were probed with a 633 nm HeNe laser, which provided sufficient Raman scatter intensity without damaging PEDOT:PSS samples. The Raman peaks were fitted with a pseudo‐Voigt function to quantify the peak center and surface area, and the background was subtracted after peak‐fitting. In literature, many factors were shown to affect the absolute Raman scattering intensity, such as laser power, resonance enhancement, and film thickness.^[^ ^25^, ^43^, ^44^ ^]^ Besides, we also found that the optical focal height (Figure S6, Supporting Information), PSS/PEDOT ratio (Figure S7, Supporting Information), and film thickness (Figure S8, Supporting Information) significantly affected the PEDOT:PSS absolute Raman intensity. To eliminate these dependencies and facilitate the correlation between PEDOT Raman spectra and its intrinsic material properties (crystalline morphology and doping level), we propose an internal intensity normalization by one of the Raman peaks. We showed in Figure S6–S8 that normalizing the spectra by the C_β_–O (577 cm^−1^) peak area, we can compare different samples without the need for tedious thickness and composition calibration, while minimizing experimental errors such as defocus. We chose the C_β_–O peak for internal normalization because it is isolated, sharp, and intense (**Figure** **2**). More details about laser wavelength comparison, parameter optimization, and data processing are provided in the Experimental Method and Figures S10–S13 (Supporting Information). Noteworthily, though the Raman peaks are noted by their dominating vibration bonds, each peak is contributed by the vibrations of multiple chemical bonds rather than only one chemical bond, as shown by the EDOT chemical structure in Figure 2.^[^ ^25^, ^26^ ^]^ On a first visual inspection, several interesting features were observed in the Raman spectra of PSPT PEDOT:PSS samples in Figure 2. First, after the morphology modification by polar solvent post‐treatment, four Raman peaks remained at their peak positions: C_β_–O (577 cm^−1^, used as normalization reference), C_β_–C_β_ (1365 cm^−1^), and two peaks from the asymmetric thiophene vibration of C_α_═C_β_ (1545 and 1565 cm^−1^). The fact that the two former peaks retained their position after polar solvent post‐treatment is not surprising, considering that PSPT should induce mainly morphological transformations, and theory has revealed that none of these Raman peaks involve vibrations related to the torsional angle between EDOT thiophene rings.^[^ ^25^, ^26^ ^]^ This implies that these Raman peaks’ position, intensity, and area are theoretically independent of the PEDOT backbone planarity (or conformation). In Figure 2, the C_β_–O–C Raman peak (437 cm^−1^) position shifts to lower wavenumbers as electrical conductivity increases. Last but not least, C_α_–C_α_’ (1260 cm^−1^) and C_α_═C_β_ (1435 cm^−1^) peaks showed distinguishable and similar shifts after polar solvent post‐treatment but without apparent correlation to the electrical conductivity. Such uncorrelated shifts reflect the complicated interpretation of PEDOT morphology evolution using Raman spectra.

**Figure 3:**
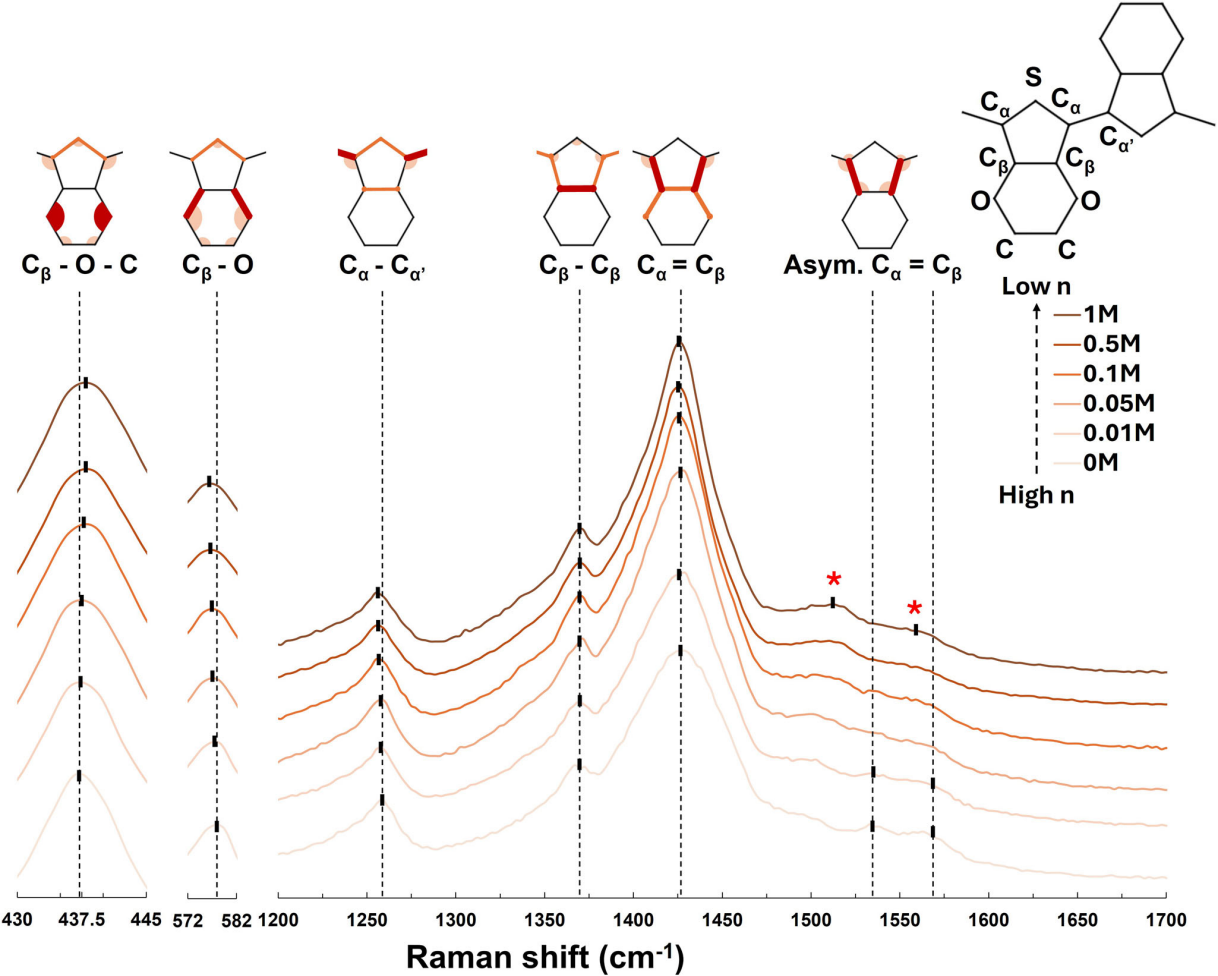
Raman spectra of Na_2_SO_3_‐reduced PEDOT:PSS thin film samples. Each presented spectrum is the average of 3 replicate samples on silicon substrates (sample population n = 3). All spectra are offset with the reported silicon Raman peak (520 cm^−1^) and normalized by the C_β_–O peak area. Most peaks significantly shifted to lower wavenumbers after the Na_2_SO_3_ post‐treatment. The two asymmetric C_α_═C_β_ peaks (1535 and 1565 cm^−1^) were replaced by two new peaks at 1515 and 1550 cm^−1^ (labeled with *) after heavy chemical de‐doping. C_α_═C_β_ (1435 cm^−1^) peak intensity increased after Na_2_SO_3_ post‐treatment. The background signal is subtracted. The contributing vibrational modes (circular sector: scissoring and rocking; line: stretching) of each peak are illustrated in orange in the EDOT molecular structure. Peaks are named by their most contributing vibrational bonding (highlighted in dark red).^[^ ^25^, ^26^ ^]^

**Figure** **3** shows the Raman spectra of Na_2_SO_3_‐reduced PEDOT:PSS thin films. Notably, post‐treatment with sodium sulfate solutions affected all peaks in PEDOT Raman spectra. All peaks had noticeable shifts toward lower wavenumbers, likely because the chemical reduction changes the electron distribution and bond length of the entire EDOT units.^[^ ^45^ ^]^ As a result, the C_β_–O peak shift, which disregards morphological changes in Figure 2, can be used as an explicit indicator of PEDOT doping level modification. Moreover, as PEDOT:PSS was gradually reduced by increasing Na_2_SO_3_ concentration, the intensity of the two asymmetric C_α_═C_β_ peaks (1535 and 1565 cm^−1^), which did not change in Figure 2 neither, started decreasing until they were eventually replaced by two new peaks centered at 1515 and 1550 cm^−1^. The spectral evolution of the asymmetric C_α_═C_β_ peaks has been reported in both electrochemical^[^ ^31^ ^]^ and chemical^[^ ^46^ ^]^ de‐doped PEDOT as an identifying characteristic of PEDOT reduction. In addition, the C_α_═C_β_ peak shifted toward a lower wavenumber and enhanced its intensity after chemical reduction. The trend of C_α_═C_β_ peak shifting due to Na_2_SO_3_ post‐treatment resembled Jialong Peng et al.’s observation.^[^ ^31^ ^]^ According to their DFT calculation, neutral EDOT units contribute a C_α_═C_β_ Raman peak ≈1402 cm^−1^ with a strong intensity, while oxidized EDOT units contribute a C_α_═C_β_ Raman peak at 1417 cm^−1^ with a relatively low intensity. When the neutral EDOT ratio increases due to the reduction reaction, the C_α_═C_β_ peak is gradually dominated by the lower wavenumber neutral EDOT C_α_═C_β_ Raman signal. Therefore, the measured C_α_═C_β_ peak shifts from 1417 cm^−1^ toward 1402 cm^−1^ and increases its Raman scatter intensity. However, the absolute value of the C_α_═C_β_ peak Raman shifts in our results (all larger than 1417 cm^−1^) were not consistent with the ones reported by Jialong Peng et al. (1402–1417 cm^−1^).^[^ ^31^ ^]^ As a result, we could not apply their theoretical approach to estimate the PEDOT doping levels of our samples, neither for the PSPT nor for the Na_2_SO_3_‐reduced samples. The inconsistency in the observed C_α_═C_β_ peak positions can be explained by two main factors: PEDOT chain length and substrate effect. Tsoi et al.’s DFT result showed that the number of repeating units (namely, conjugation length) of P3HT significantly shifted the P3HT C_α_═C_β_ peak to a lower wavenumber by as much as 23 cm^−1^.^[^ ^47^ ^]^ The PEDOT in Peng et al.’s work was electrochemically polymerized on the surface of gold nanoparticles,^[^ ^31^ ^]^ which differs from the traditional oxidation polymerization of PEDOT:PSS.^[^ ^48^ ^]^ Thus, the resulting PEDOT may significantly differ in terms of repeating units (the chain length), leading to the encountered C_α_═C_β_ peak position mismatch. Also, in the Experimental Section (Supporting Information) of Peng et al.’s report, they mentioned that “to obtain best‐fit with experimental SERS for PEDOT, interaction with a gold atom is necessary in calculations”. Our PEDOT:PSS samples do not contain gold and are deposited on silicon. Such a difference in the substrate might also affect the absolute position of the PEDOT C_α_═C_β_ Raman peak.

To study the details of relevant Raman features, each peak position, normalized area, and full width at half maximum (FWHM) were correlated with the corresponding GIWAXS and XPS measurements, yielding the correlation heatmaps shown in Table S2 (Supporting Information). We narrowed the analysis down to the parameters showing the highest absolute correlations: π–π stacking distance (d_π–π_) and doping level (DL). We evaluated both linearity, using the Pearson linear correlation factor, and monotonicity, using Spearman's rank correlation coefficient, and defined the significance of the correlation with an ANOVA test. The main correlation results are summarized in **Table** **2**. Note that some modest correlations, such as the FWHM of the C_β_–C_β_ peak with coherence length and paracrystallinity, are left out for the sake of simplicity. Linearity and monotonicity are necessary for the fitting of Equations (1) and (2). The criteria for significant correlation between a Raman feature and a measured parameter are (1) the absolute value of both Pearson and Spearman's correlation coefficients is higher than 0.5, and (2) the p‐value of both correlations is lower than 0.1 (signifying a 90% confidence interval). For the cases with any correlation coefficient lower than 0.5, we compared the root mean square error (RMSE) between the measured and fitted value for all the samples, to the experimental error, i.e., the average standard deviation for all the samples ($\bar{\sigma }$). If RMSE > 2$\bar{\sigma }$, the corresponding Raman peak might be linked to factors affected by post‐treatments but not captured by GIWAXS and XPS (for example, torsional angle between EDOT units). In such a case, the correlation was noted as “?”. Finally, those Raman features displaying a low correlation coefficient and RMSE < 2$\bar{\sigma }$, are considered independent. That means that these features do not change with post‐treatments, and the minor peak changes observed are attributed to measurement errors. Because the asymmetric C_α_═C_β_ peaks have low intensities in most Raman spectra, they are not included in the correlation analysis.

**Table 2:** Raman peak evolution summary of PSPT and Na_2_SO_3_‐reduced PEDOT:PSS samples.

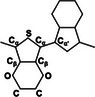	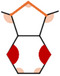 C_β_–O–C			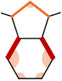 C_β_–O		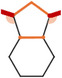 C_α_–C_α’_			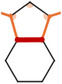 C_β_–C_β_			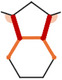 C_α_═C_β_		
	Δ$\tilde{\nu }$	A’	W	Δ$\tilde{\nu }$	W	Δ$\tilde{\nu }$	A’	W	Δ$\tilde{\nu }$	A’	W	Δ$\tilde{\nu }$	A’	W
Compact PEDOT crystal (↓ d_π–π_)	–	+	=	=	–	=	=	?	=	+	=	?	+	=
De‐doping (↓ DL)	+	+	=	–	+	?	=	+	=	+*	=	?	+*	–

d_π–π_: PEDOT intermolecular distance in π–π crystalline direction. DL: PEDOT doping level, ≡ [EDOT^+^]/([EDOT^+^]+[EDOT^0^]); Δ$\tilde{v}$: peak position; A’: peak area normalized by C_β_–O peak area; W: peak width (full‐width at half maximum, FWHM); The position shifting, area increasing, and peak narrowing are noted when the absolute value of Pearson AND Spearman correlation > 0.5 and p‐value < 0.1 (90% confidence interval); “ * ”: 85% confidence interval (0.1 < p < 0.15). “? ”: Pearson OR Spearman correlation < 0.5, but the root mean square error (RMSE) between the samples measured value and their fitted value is twice higher than the average standard deviation of the samples (2$\bar{\sigma }$). It implies peak change could be contributed by other factors, such as backbone conjugation length, which concurs with morphology change or de‐doping; “ = ”: Pearson OR Spearman correlation < 0.5 and RMSE < 2$\bar{\sigma }$, which means the feature difference between samples fits within the measurement error (feature does not change with post‐treatment).

From Table 2, the position and area of the C_β_–O–C peak were the selected features to derive the estimation equations Equations (1) and (2), for the following reasons; (1) the C_β_–O–C peak is an isolated and sharp peak with high intensity which provides a high fitting precision; (2) as a result its position and area present the two highest Pearson and Spearman correlation coefficients and significances (p < 0.08, 92% confidence interval in the worst case) to both PEDOT d_π–π_ and doping level in Table S2 (Supporting Information); and (3) the C_β_–O–C peak shifts toward opposite directions for d_π–π_ and doping level reduction, which results in a high sensitivity to distinguish these two effects. Moreover, the following equations incorporate the C_β_–O peak for the relative shift $\left(\Delta \upsilon_{C_{\beta }-O-C}-\Delta \upsilon_{C_{\beta }-O}\right)$ and the normalized area ($\frac{A_{C_{\beta }-O-C}}{A_{C_{\beta }-O}}$) to avoid mis‐calibrated Raman shifts and undesired intensity fluctuation, respectively. The C_β_‐O peak is suitable for the equations because it does not introduce uncertainty: its relative shift shows a significant correlation with DL and no correlation at all with d_π–π_. Although other factors, such as the FWHM of peak C_β_–O, could have been added to the equation, they were not used to avoid overfitting, since solving a two‐factor problem requires only two equations with two variables. Herein are the two empirical equations proposed to estimate π–π stacking distance (d_π–π_) and doping level (DL) from Raman features (more details of their derivation are discussed in the Supporting Information):

(1) $$\begin{array}{ccc} d_{\pi -\pi }\left(\mathrm{Angstrom}\right) & = & 0.0144\times \left(\Delta \upsilon_{C_{\beta }-O-C}-\Delta \upsilon_{C_{\beta }-O}\right)-0.0413\\ & & \times \frac{A_{C_{\beta }-O-C}}{A_{C_{\beta }-O}}+5.5114 \end{array}$$

(2) $$\begin{array}{ccc} DL\;\left(\%\right) & = & -4.60\times \left(\Delta \upsilon_{C_{\beta }-O-C}-\;\Delta \upsilon_{C_{\beta }-O}\right)\\ & & -6.51\times \frac{A_{C_{\beta }-O-C}}{A_{C_{\beta }-O}}-593.77 \end{array}$$

where $\Delta \upsilon_{C_{\beta }-O-C}$ and $\Delta \upsilon_{C_{\beta }-O}$ are the centers of C_β_–O–C and C_β_–O Raman peaks, and the $A_{C_{\beta }-O-C}$ and $A_{C_{\beta }-O}$ are the areas of C_β_–O–C and C_β_–O Raman peaks, respectively.

**Figure 4:**
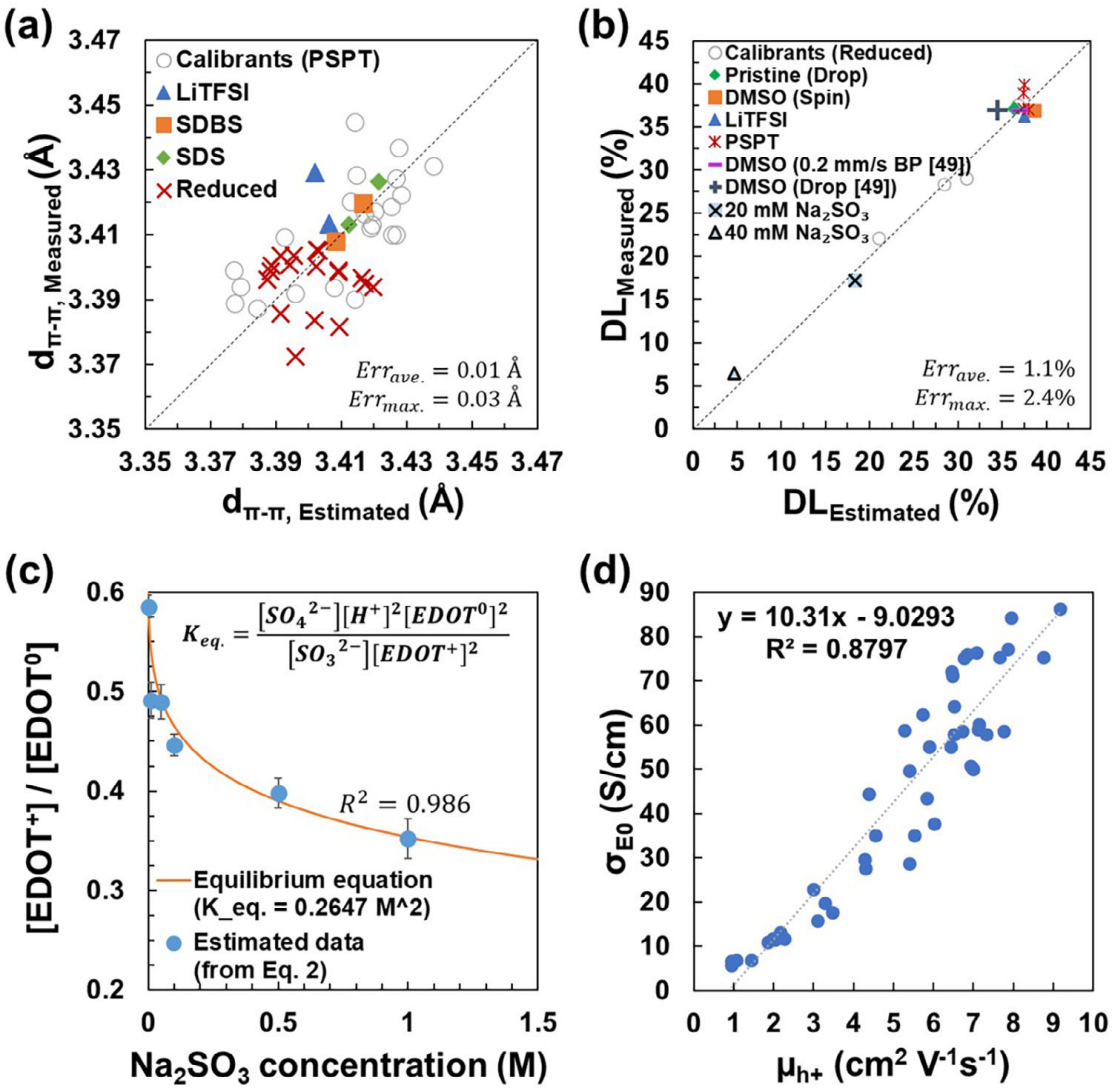
a) Comparison between the PEDOT π–π intermolecular spacing (*d_π–π_*) measured from GIWAXS and the estimated *d_π–π_* from PEDOT Raman spectra with Equation 1. b) Comparison between the PEDOT doping level (*DL*) measured from XPS versus the estimated *DL* from Raman spectra with Equation 2. Drop‐coated (Drop) and brush‐printed (BP) samples with radically different morphology but identical composition are added for completeness.^[^ ^49^ ^]^ Heavily de‐doped samples (20 mm Na_2_SO_3_ and 40 mm Na_2_SO_3_) were prepared by spin‐coating Na_2_SO_3_‐mixed PEDOT:PSS solution following the procedure reported by Nitin Saxena et al.^[^ ^30^ ^]^ Our numerical model proved to predict PEDOT doping level down to ≈5%. c) The concentration ratio of doped and neutral EDOT units (a factor correlated to doping level) versus applied Na_2_SO_3_ concentration, comparing the estimation using Equation 2 (and Equation S6, Supporting Information) and the chemical equilibrium equations Equation 3 and Equation 4 with K_eq._ = 0.2647 M^2^ (sample population n = 3), values: mean ± standard error. d) The transport coefficient from *Kang‐Snyder* versus the estimated hole mobility shows a linear relationship.

The proposed equations were verified with additional PEDOT:PSS test samples blended with different typical secondary dopants, such as dimethyl sulfoxide (DMSO), lithium bis(trifluoromethanesulfonyl)imide (LiTFSI), sodium dodecylsulfonate (SDS), and sodium dodecylbenzenesulfonate (SDBS), respectively (**Figure** **4**a,b). The GIWAXS and XPS results for these test samples can be found in Table S3 (Supporting Information). Figure 4a shows the difference between the estimated PEDOT d_π–π_ from Raman spectra (Equation 1) and the measured PEDOT d_π–π_ from GIWAXS results. Despite the different nature of the secondary dopants and post‐treatments tested, the proposed equation provides a decent estimation of PEDOT d_π–π_ (see fitting to average values for each condition in Figure S14, Supporting Information), with an average error of ≈0.01Å and a maximum error of 0.03Å, which is a few times lower than the maximum dispersion of d_π–π_ ≈0.075 Å measured for all the PEDOT‐based materials in Figure 4a. Nevertheless, being the maximum estimated error in the same order of magnitude as the maximum measured dispersion, we conclude that Equation 1 only provides a rough picture of PEDOT crystalline morphology change rather than a precise characterization.

Equation 2, on the contrary, fits well the XPS measured doping level values of PEDOT:PSS not only of the calibrants used to draw the equation, but also for the test samples containing additives (LiTFSI and DMSO), as shown in Figure 4b. Furthermore, the figure includes drop‐coated and brush‐printed PEDOT:PSS (with 5 vol.% DMSO) samples reported by our group elsewhere.^[^ ^49^ ^]^ In that previous work, we demonstrated that compared to the drop‐coated films, the shear stress induced by brush‐printing promoted crystalline phase, reduced π–π stacking distance, decreased paracrystallinity, and increased coherence length in the samples brush‐printed at 0.2 mm s^−1^. As a result of this purely physical treatment, the conductivity doubled, reaching ≈750 S cm^−1^ without any chemical post‐treatment. As expected, and despite the radically different morphology of drop‐coated and brush‐printed samples, the predicted doping level for both sample sets was almost identical and close to the non‐reduced PEDOT:PSS samples (Figure 4b). This result generalizes the validity of Equation 2 to samples whose morphology is tuned by mechanical forces rather than wet post‐treatments. To test if our numerical model can estimate PEDOT doping levels below 22% (the lowest doping level of calibrants in the model), two heavily de‐doped PEDOT:PSS samples were prepared by following the procedure reported by Nitin Saxena et al.^[^ ^30^ ^]^ Sodium sulfite was directly added into PEDOT:PSS suspension to prepare 20 and 40 mm Na_2_SO_3_ solutions, respectively, which were spin‐coated to form thin film samples. Their estimated PEDOT doping levels (by applying Equation 2 to their Raman spectra) were comparable with those calculated from their XPS spectra, as shown in Figure 4b. The result confirms the capability of our numerical model for estimating the PEDOT doping level over a wide range (from 40% down to 5%). The average and maximum errors of the estimated doping level were only ≈1% and 2.4%, respectively, which are far below the measured dispersion in the samples doping level (~35%).

The relation between the applied Na_2_SO_3_ concentration and the estimated PEDOT doping level was further investigated to confirm the validity of Equation 2, as shown in Figure 4c. According to the literature,^[^ ^30^ ^]^ the chemical reaction between sulfite anion (SO_3_^2−^) and PEDOT can be described as follows,

(3) $$2EDOT^{+}+S{O_{3}}^{2-}+H_{2}O⇌2EDOT^{0}+S{O_{4}}^{2-}+2H^{+}$$

Considering that SO_3_^2−^ is a mild reagent and the reduction reaction is reversible, the final concentrations of the involved species should follow the equilibrium equation (Equation 4),

(4) $$K_{eq.}=\frac{{\left[EDOT^{0}\right]}^{2}*\left[S{O_{4}}^{2-}\right]*{\left[H^{+}\right]}^{2}}{{\left[EDOT^{+}\right]}^{2}*\left[S{O_{3}}^{2-}\right]}$$

where K_eq_. is the equilibrium constant. Figure 4c shows that Equation 2 successfully retraces Equation 4 with a fitted K_eq_. = 0.2647M^2^ (detailed description in the Supplementary Information). The fitted DL_0_ (PEDOT doping level before reduction) is 38.77%, close to the XPS results shown in Table 1.

From Equation 2, the PEDOT:PSS carrier concentrations and carrier mobilities (µ) can be easily calculated (the detailed calculation is described in the Supporting Information). The carrier mobilities (µ) of all presented samples are plotted in Figure 4d against the corresponding transport coefficient (σ_E0_) obtained from fitting their thermoelectric properties with the Kang‐Snyder model (Figure S9, Supporting Information). Though the two factors are calculated based on different charge transport models (Drude's model versus Kang‐Snyder model), they are strongly correlated. The determination of linear correlation R^2^ is higher than 0.8. In the Kang‐Snyder model, the charge carriers with energy below the transport edge energy (E_t_) have no contribution to electrical conductivity, and those above E_t_ contribute with a weighted conductivity, the transport coefficient (σ_E0_). The transport behavior of PEDOT with doping levels > 20% isdescribed as band‐like transport in the literature.^[^ ^32^, ^35^ ^]^ As a result, the transport coefficient (σ_E0_) and carrier mobility (µ) from Drude's model should be directly proportional, as shown in Figure 4d. The estimated values of mobility matched those reported in the literature for PEDOT:PSS subjected to wet post‐treatments.^[^ ^50^ ^]^ This correlation bolsters the validity of our approach to estimating PEDOT doping levels from their Raman spectra.

**Figure 5:**
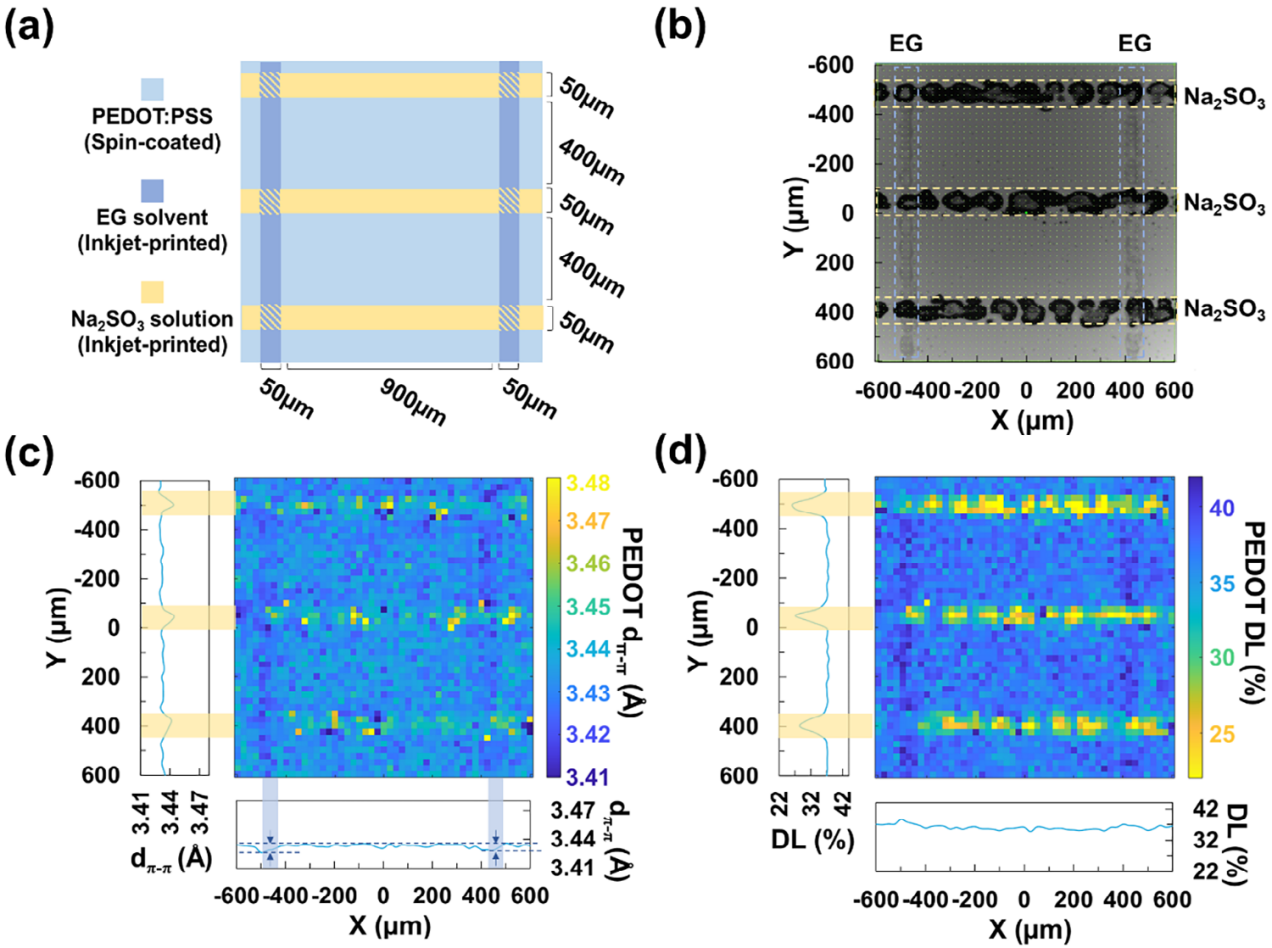
a) Layout used for the inkjet‐printed (IJP) local post‐treatment of PEDOT:PSS thin films. The sample was locally post‐treated by IJP lines of ethylene glycol and 0.1 mm Na_2_SO_3_ solution perpendicular to each other. b) optical microscope image of the IJP‐patterned PEDOT:PSS thin film. c,d) Mapping of d_π‐π_ and doping level (DL) for the IJP‐post‐treated PEDOT:PSS thin film (sample population n = 1). The spacing between mapping points is 25 µm in both X and Y directions. The profiles next to the mapping images show the average PEDOT d_π–π_ and DL from pixels at the same X or Y coordinate.

Finally, we demonstrated our model for probing the local PEDOT crystalline structure and doping level. Ethylene glycol and Na_2_SO_3_ (0.1 m) solution were sequentially inkjet‐printed (IJP) on a spin‐coated pristine PEDOT:PSS thin film for selective post‐treatment according to the layout shown in **Figure** **5**a. The IJP pattern was confirmed by the optical microscope image shown in Figure 5b, where the black dots are the precipitated Na_2_SO_3,_ and drying marks can be appreciated from the ethylene glycol lines. Note that the actual line width of 60–100 µm is larger than the layout due to drop spreading. PEDOT:PSS Raman spectra were collected every 25 µm in both X and Y direction, and d_π–π_ and DL were calculated by applying Equations 1 and 2, shown in Figure 5c,d. The average d_π–π_ and DL from pixels at the same X or Y coordinate were calculated and plotted next to the corresponding axis. In the region without any IJP post‐treatment, the calculated PEDOT d_π‐π_ and doping level were consistent with the results in Figure 1b and Table 1, ≈3.433 Å and 37%, respectively. For the regions post‐treated with ethylene glycol by IJP (X = −500 and 400 µm), the DL remained unchanged as expected. However, we observed a fainter decrease in PEDOT d_π–π_ than expected: 3.426 Å Vs 3.39 Å in Figure 1b. The difference might arise from two significant differences in the post‐treatment processes. Compared to the IJP post‐treated films, the reference samples in Figure 1b were coated with ethylene glycol and subsequently spun by a spin‐coater to remove the excess of ethylene glycol. Spinning introduces a shearing force on the solvent that might have contributed to stretching PEDOT:PSS molecules and reducing d_π–π_.^[^ ^17^, ^49^ ^]^ Moreover, the spin‐coating process removed part of the PSS (Table 1), which was not the case with local IJP post‐treatment. On the other hand, the regions IJP with Na_2_SO_3_ solution (Y = −500, −50, and 400 µm) show a clear difference in both PEDOT d_π–π_ and doping level. Upon treatment with 0.1 M Na_2_SO_3_ solution, PEDOT doping level was reduced to ≈27%, consistent with the results in Table 1. On the contrary, the d_π–π_ distance did not change in those areas overlapping with ethylene glycol, which was expected because those areas received a similar post‐treatment as the blank films in Figure 1e (corresponding to the EG + Na_2_SO_3_). However, d_π–π_ interestingly increased slightly to ≈3.44 Å upon treatment with Na_2_SO_3_ solution in those areas where ethylene glycol was not previously deposited. We did not study films treated only with Na_2_SO_3_ solution in GIWAXS to corroborate this d_π–π_ increase. However, some published reports showed that PEDOT crystals favor larger intermolecular spacing, d_π–π_, for lower doping levels. Shi et al. reported a thermodynamically stable PEDOT crystal structure calculated by the DFT method, where the d_π–π_ of lightly doped PEDOT:Tos was larger than that of heavily doped material (d_π–π_ = 3.885Å for DL ≈41% versus d_π–π_ = 3.915Å for DL ≈20%).^[^ ^45^ ^]^ This report seems to support the increased d_π‐π_ in Figure 5c where PEDOT:PSS is only IJP‐treated with Na_2_SO_3_. Overall, the PEDOT d_π–π_ and DL values obtained from 2D Raman mapping aligned well with the designed layout, the optical image of the patterned film, and the measured d_π–π_ and DL with GIWAXS and XPS, respectively, on comparable homogeneously post‐treated samples. The results demonstrated the capability of our method for characterizing local properties of patterned PEDOT:PSS materials, which holds great potential to study PEDOT:PSS integrated in microdevices, such as thermoelectrics or organic electrochemical transistors, even in operando conditions.

## 3 Discussion

In the present research, the estimation equations are applicable for PEDOT d_π–π_ between 3.37 and 3.43 Å and doping levels ranging from 5% to 38%. In the future, experimental data with a wider span could expand the estimation window. Also, a machine learning model (such as generalized additive model, random forest, and neural network) might improve the estimation precision for a wider PEDOT d_π‐π_ and doping level range. Theoretically, the doping level of pristine PEDOT:PSS (≈33%) is close to the saturated PEDOT doping level.^[^ ^37^, ^38^ ^]^ Further oxidation could denature the thiophene ring of PEDOT, causing irreversible changes in the material properties and Raman spectra.^[^ ^51^ ^]^ Therefore, our approach covers at least the upper doping limit of the PEDOT‐based materials. Furthermore, Equation 2 in combination with Equation S6 (Supporting Information) can estimate the hole mobility in PEDOT:PSS if the PSS‐to‐PEDOT ratio is known. Since the PSS‐to‐PEDOT ratio is well defined for commercial formulations, our proposed equations will serve to estimate the mobility of PEDOT:PSS films as long as they are not treated with processes involving uncontrolled PSS removal. Moreover, even for those washing steps removing PSS, the thickness change could be used presumably as an indicator of PSS removal, expanding further the usefulness of the proposed equations.

The proposed framework could be extended to other existing Raman‐based techniques, such as segregation‐enhanced Raman spectra (SERS), tip‐enhanced Raman spectra (TERS), and polarized Raman, which is a powerful tool for studying PEDOT:PSS orientation and anisotropic morphology.^[^ ^49^ ^]^ Overall, merging the proposed theoretical framework with Raman spectroscopy will enable the operando characterization of PEDOT‐based electronics, such as organic electrochemical transistors (OECTs) and thermoelectrics (OTEs), and real‐time monitoring of PEDOT synthesis and fabrication in streamlined production, facilitating the future development of PEDOT‐based electronics.

## 4 Conclusion

In the present study, the morphology and chemical reduction effects on PEDOT:PSS Raman spectra were investigated with two series of samples: polar‐solvent post‐treated (PSPT) PEDOT:PSS thin films and Na_2_SO_3_‐reduced PEDOT:PSS thin films. Their PEDOT doping level and crystalline structure were measured by XPS and GIWAXS, respectively, and correlated to Raman spectra to identify features able to unequivocally differentiate between both properties. The PEDOT C_α_═C_β_ Raman peak (1435 cm^−1^), usually employed by the community, is not an ideal analysis feature because its shifting does not show a clear correlation with doping level or crystallinity changes. This is likely because the PEDOT C_α_═C_β_ Raman peak shifting represents morphology not detectable in GIWAXS, e.g., PEDOT backbone torsional angle. Moreover, the PEDOT C_α_═C_β_ Raman peak area correlates only weakly with doping level and crystallinity changes. We identified signature Raman features that allow distinguishing between doping level and PEDOT π–π stacking distance, either because their change is exclusive to any of those properties, or because they change in opposite directions. Two empirical equations are proposed to estimate PEDOT doping level and π–π intermolecular spacing from PEDOT:PSS Raman spectra. The proposed equations were verified with additive‐loaded PEDOT:PSS samples and brush‐printed samples. The PEDOT d_π‐π_ estimation equation (Equation 1) showed moderate agreement with GIWAXS results, offering a general picture of morphology change. On the other hand, the PEDOT doping level equation (Equation 2) successfully covered a wide range of PEDOT doping levels and demonstrated a low estimation error compared to XPS results. With Equation 2, we could capture the Na_2_SO_3_‐PEDOT reduction reaction in agreement with its equilibrium equation and estimate carrier mobility consistent with the Kang‐Snyder model fitting results. Finally, the lateral spatial resolution of Raman is exploited to create 2D mappings of PEDOT:PSS properties, which can find application in studying the material when integrated in microdevices.

The findings of this research resolve the long‐standing confusion on PEDOT:PSS Raman spectra interpretation and provide a new analysis approach for simultaneously quantifying the doping level and crystalline structure of PEDOT:PSS. With our observation on PEDOT Raman peaks usually ignored in literature (especially C_β_‐O‐C and C_β_‐O peaks), we aim at igniting further theoretical investigation able to shed light on how changes in PEDOT morphology and oxidation level reflect on its Raman spectra. Also, we demonstrated the potential of the proposed equation to estimate the hole mobility in PEDOT:PSS if the PSS‐to‐PEDOT ratio is known. The proposed method promotes the potential of Raman spectroscopy in characterizing PEDOT‐containing materials, such as PEDOT‐based blends and nanocomposites, within seconds under ambient conditions.

## Conflict of Interest

The authors declare no conflict of interest.

## Supporting information



Supporting Information

## Data Availability

The data that support the findings of this study are openly available in the KU Leuven RDR repository (DOI: 10.48804/3XL1MR).
