# Supplementary material for: Unleashing the Potential of Raman Spectroscopy to Estimate PEDOT:PSS Doping Level and Crystalline Morphology
Source: Adv Sci (Weinh). 2025 Dec 5;13(9):e13726. doi: 10.1002/advs.202513726 (PMC12904090; doi:10.1002/advs.202513726)
Supplement: Supplementary file 1 — Supporting Information [file ADVS-13-e13726-s001.docx]

Supporting Information

Unleashing the Potential of Raman Spectroscopy to Estimate PEDOT:PSS Doping Level and Crystalline Morphology

Tzu-Yi Thomas Yu, Tsu-Yu Chou, Viktor Naenen, Bokai Zhang, Hasan Emre Baysal, Bora Bulut, Martin Rosenthal, Francisco Molina-Lopez*

Tzu-Yi Thomas Yu, Tsu-Yu Chou, Viktor Naenen, Bokai Zhang, Hasan Emre Baysal, Bora Bulut, Dr. Francisco MOLINA-LOPEZ

Department of Materials Engineering (MTM), KU Leuven, Leuven, Belgium

E-mail: francisco.molinalopez@kuleuven.be

Dr. Martin Rosenthal
Department of Chemistry, KU Leuven, Leuven, Belgium

Dr. Martin Rosenthal
European Synchrotron Radiation Facility (ESRF), Grenoble, France

Table of Contents

[**• Experimental Section/Methods** 3](#_Toc213362438)

[• **Supporting Figures** 9](#_Toc213362439)

[• **Supporting Tables** 25](#_Toc213362440)

[• **Derivation of empirical equations to evaluate PEDOT *d_π-π_* and doping level** 28](#_Toc213362441)

[• **Equilibrium equation fitting of applied Na_2_SO_3_ concentration and PEDOT doping level** 31](#_Toc213362442)

[• **Estimation of carrier mobility of PEDOT:PSS samples** 35](#_Toc213362443)

[• **References:** 36](#_Toc213362444)

- Experimental Section/Methods

*Materials:*

Poly(3,4-ethylenedioxythiophene): polystyrene sulfonate (PEDOT:PSS, Clevios PH 1000, ID-No. 81076212) was purchased from Heraeus. Ethanol absolute (EtOH, >=99.8%, Lot: 2489791) was purchased from Fisher Scientific. Ethylene glycol (EG, 99.5%, Lot: 1728159) was purchased from Acros Organics. Sodium sulfite (Na_2_SO_3_, 98+%, Lot: 09215JA), sodium dodecyl sulfonate (SDS, BioReagent 98.5+%), (3-glycidyloxypropyl)trimethoxy-silane (GOPS, >=98%, Lot: STBK3859), and sodium dodecyl-benzenesulfonate (SDBS, technical grade, Lot: MKBK8221V) were purchased from Aldrich Chemistry. Lithium bis(trifluoromethanesulfonimide) (LiTFSI, 99%, Lot: A0448577) was purchased from Thermo Scientific Chemicals. All chemicals were used as received without further purification.

*PEDOT:PSS thin film preparation:*

2.5*2.5 cm^2^ Superfrost Plus^TM^ glass slides (Avantor VWR, Cat. No. 631-0108) were utilized as substrates for preparing electrical conductivity, Seebeck coefficient, and Raman characterization samples. Before spin-coating PEDOT:PSS solutions, substrates were ultrasonicated with a detergent aqueous solution, DI water, ethanol, acetone, and isopropanol, respectively. Then, substrates were dried with N_2_ flow and cleaned with UV-ozone cleaner (NanoBioAnalytics, UVC-1014) for 30 minutes. 5 nm Pt/Pd was sputtered on cleaned glass substrates as bottom electrodes for electrical conductivity and Seebeck coefficient measurement. After applying bottom electrodes, the substrates were again ultrasonicated, dried, and cleaned with the abovementioned procedures. GIWAXS and XPS samples were supported by 1.25*1.25 cm^2^ silicon substrates. Silicon substrates (cut from 100 mm P/Bor <100> silicon wafer, Si-Mat) were cleaned with the same procedure as glass substrates without applying bottom electrodes.

For polar-solvent post-treated samples, PH1000 solution was stirred at room temperature overnight. PEDOT:PSS thin film samples were prepared by spin-coating PH1000 solution on cleaned substrates with a three-step process; 500 rpm (acceleration rate: 250 rpm/s) for 10 seconds, 1000 rpm for 2 minutes (acceleration rate: 250 rpm/s), and 8000 rpm for 2 seconds (acceleration rate: 4000 rpm/s). The resulting PEDOT:PSS thin films are about 60 nm in thickness. Then, PEDOT:PSS thin films were thermally annealed at 120 °C for 10 minutes. After cooling down to room temperature, the polar solvent post-treatment was applied. For solvent liquid post-treatment, EtOH or EG was spin-coated on top of thin film samples at 3000 rpm for 30s. For solvent vapor post-treatment, thin film samples were held in a vacuum chamber (~ 10 Torr) overnight alongside a vial containing the polar solvent. For both liquid and vapor post-treated samples, solvent residues were finally removed by vacuum.

For preparing de-doped samples, the cleaned substrates were treated with GOPS following the reported procedure.^[1]^ Substrates were immersed in 1.5% v/v GOPS in ethanol solution for 1 hour, followed by ethanol rinsing. Then, PH1000 solution is spin-coated on pre-treated substrates with the 3 steps previously described. Samples were then thermally annealed at 120 °C for 10 minutes. EG liquid is further spin-coated on PEDOT:PSS thin film with 3000 rpm for 30s and thermal annealed for another 10 minutes under 120^o^C. To de-dope PEDOT:PSS thin films to different degrees, Na_2_SO_3_ aqueous solution with varied concentrations were dropped on top of thin film samples for 10 minutes. After treatment, samples were rinsed with DI water carefully and dried with N_2_ flow.

Heavily de-doped samples were prepared following the procedure reported by Nitin Saxena *et al*.^[2]^ Na_2_SO_3_ was directly blended with PEDOT:PSS suspension (PH1000) to form 20 mM and 40 mM Na_2_SO_3_ /PH1000 solution. The thin film samples were prepared with the same spin-coating parameters (500 rpm for 10 seconds, 1000 rpm for 2 minutes, and 8000 rpm for 2 seconds) as the samples mentioned above. After annealing at 120 °C for 10 minutes, EG liquid is further spin-coated on PEDOT:PSS thin film with 3000 rpm for 30s and thermal annealed for another 10 minutes under 120 °C.

*Ink-jet printing (IJP) for local post-treatment of PEDOT:PSS thin films*

Ink-jet printing (IJP) is applied to selectively post-treat PEDOT:PSS thin films with EG solvent and 0.1M Na_2_SO_3_ aqueous solution according to the designed patterns. IJP was carried out by a Dimatix Material Printer DMP-2850 from Fujifilm with *Samba®* cartridge module (minimum 2.4pL droplet size). First, pristine PEDOT:PSS thin films were prepared on silicon substrates following the same cleaning process, GOPS-modification, spin-coating procedure, and thermal annealing post-treatment as described in the previous section. The IJP inks were degassed under vacuum and loaded into the cartridges through a syringe filter. The printer plate was kept at 60 ^o^C. Ten consecutive layers of ethylene glycol were printed on the pristine PEDOT:PSS thin film, leaving a 10-second pause between layers (printing parameters: 7 kHz, drop-to-drop spacing: 10 μm, nozzle temp.: 35 ^o^C). Then, another ten layers of 0.1M Na_2_SO_3_ solution were printed, leaving a 3-second pause between layers (printing parameters: 7 kHz, drop-to-drop spacing: 5 μm, nozzle temp.: room temp.). Finally, the IJP-patterned PEDOT:PSS samples were annealed at 120 ^o^C for 10 minutes to remove the residual solvents.

*Electrical conductivity and Seebeck coefficient measurement*

Resistance measurement was performed under ambient conditions by a multimeter (Keithley, 2000 multimeter) with a 4-wire setup. After spin-coating, parts of PEDOT:PSS film are carefully removed by wet swabs to form a well-defined stripe, about 1*2.5 cm^2^. Silver ink (DM-SIP-2006, DYCOTEC) is applied on Pt/Pd bottom electrodes for better contact with metal probes. Film thickness was measured by Profilometer (Formtracer CS-3200, Mitutoyo). Seebeck coefficient was measured with a homemade setup described in previous reports.^[3–5]^ Seebeck voltages under 5 temperature differences were recorded (Thermometer, TENNA 72-7712), and the Seebeck coefficient was calculated by the slope of voltage-temperature difference with the least squares regression method. The Seebeck coefficient measurement is calibrated with a constantan reference to compensate for the Seebeck of the probes (+ 7 μV/K is added to the measured value).

*Kang-Snyder model analysis*

The *Kang-Snyder* (*K-S*) model was applied to analyze the charge transport behavior within PEDOT:PSS thin films.^[6]^ In the *K-S* model, electrical conductivity can be described by the following equation

$$\sigma= \sigma_{E0}\cdot s\cdot F_{s-1}(\eta)$$

where $\sigma_{E0}$ is the transport coefficient, *s* is the transport parameter, and $\eta$ is the reduced chemical potential defined as $\eta=\frac{E_{f}-E_{t}}{k_{B}T}$. $\eta$ is a function of temperature and the difference between the Fermi-level energy, $E_{f}$, and transport edge energy, $E_{t}$. The Seebeck coefficient is included in the *Kang-Snyder* model described with the following equation:

$$S= \frac{k_{B}}{e}\cdot\left[ \frac{(s+1)\cdot F_{s}(\eta)}{s{\cdot F}_{s-1}(\eta)}-\eta\right]$$

as a function of transport parameter (*s*) and reduced chemical potential ($\eta)$. According to the literature ^[6]^, PEDOT:PSS can be fitted with s = 1 in *K-S* model because charges are fully delocalized. Therefore, $\sigma_{E0}$ and $\eta$ can be estimated by fitting experimental $\sigma$-*S* curve. $\sigma_{E0}$ takes the role of a “weighted mobility”. Thus, it indicates if the electrical conductivity change is due to the morphological or chemical change in the present study. Since the PEDOT doping level of all samples is above 20%, the PEDOT critical doping level for charge delocalization reported by Shawn A. Gregory *et al.*^[7]^, charge localization should be ignored, and the *Kang-Snyder* model should describe well the charge carrier transport behavior in the present study. The *Kang-Snyder* model fitting results are shown in **Figure S9**.

*Raman characterization and data process*

Raman characterization of PEDOT:PSS thin films samples were performed with Horiba Jobin-Yvon Labram HR Evolution confocal. After machine warm-up and automatic calibration with a standard (NIST-certified Horiba SP-RCO calibration objective), PEDOT:PSS Raman spectra were collected. The parameters of Raman characterization were optimized for each sample to obtain a sufficient signal-to-noise ratio and prevent samples from being denatured by the laser. 633-nm HeNe laser (max power: 17mW) was utilized to minimize substrate noise and laser damage (**Figure S10-12**). The optimized measurement condition is 10% power (1.7 mW) for 3 seconds to avoid PEDOT:PSS denaturation (**Figure S13**) while maintaining a moderate signal-to-noise ratio. More than 18 spectra from different spots were collected for each sample to represent the average Raman spectra. The Raman spectra of substrates and solvents used in this research (**Figure S15-16**) were also collected to ensure all signals in PEDOT:PSS Raman spectra are contributed by PEDOT. PEDOT:PSS Raman spectra were individually fitted by the pseudo-Voigt function to define peak centers and areas, following the reported protocol.^[8]^ The example of Raman spectrum fitting is shown in **Figure S17**. For samples supported by silicon substrates (those used for XPS and GIWAXs, and hence for the correlation in **Table S2**), Raman spectra were offset to match the measured position of the silicon vibrational peak with the reported value at 520 cm^-1^.^[9,10]^ Furthermore, the area of 577 cm^-1^ vibrational peak was used to normalize the individual spectra to eliminate the thickness effect on absolute intensity.

For mapping the IJP-patterned samples, the scanning area was 1200 µm * 1200 µm. Raman spectra were collected by every 25 µm in x and y direction (scanning mesh size: 25 µm) with 633 nm HeNe laser with 10% power (1.7 mW). The exposure time for each point was 2 seconds. To improve the spectra quality, spectral auto-focus for maximizing the PEDOT C_α_ = C_β_ signal (1400 – 1500 cm^-1^) was carried out at each point before collecting Raman spectra. Mapping results were fitted individually by Matlab function “lsqcurvefit” with the following fitting condition: Maximum iteration 2500, step tolerance 10^-10^, and function tolerance 10^-12^. PEDOT d_π-π_ spacing and doping level of each point was calculated with **Equation 1** and **Equation 2** described in the results section. Spectra with low PEDOT signal and strong background noises were defined as invalid pixels, shown in **Figure S18, and S19**. The PEDOT d_π-π_ spacing and doping level of invalid pixels were estimated by linear interpolating the value of nearby pixels with the Matlab function, “fillmissing2”. Finally, the PEDOT d_π-π_ spacing and doping level mapping were visualized by the Matlab function, “imagesc”.

*GIWAXS characterization and data processing*

Grazing-incidence wide-angle X-ray scattering (GIWAXS) measurement was performed at BM26 (DUBBLE) beamline, European Synchrotron Radiation Facility (ESRF), Grenoble, France. Samples were probed with 12 keV X-ray and a Pilatus 1M detector was used for collecting GIWAXS data. GIWAXS 1D line-cut was processed with GIXSGUI, a Matlab toolbox developed by Sector 8-ID-E, Argonne National Laboratory, United States.^[11]^ 2D GIWAXS data with q range from 0.2 to 2 Å, and polar angle (χ, chi) from 0^o^ to 90^o^ was reduced into 1D line-cut. The peak fitting was performed by a Matlab function, “lsqcurvefit”, with the pseudo-Voigt function, a linear combination of Gaussian and Lorentzian functions. The GIWAXS peak fitting procedure follows the reported protocol.^[12]^ The example of GIWAXS 1D line-cut fitting is shown in **Figure S20**. The PEDOT (020) peak position was transformed into PEDOT π-π spacing, and the full-width-at-half-maximum (FWHM) of PEDOT (020) peak was converted into crystal coherence length in π-π direction (L_c, π-π_) with Scherrer’s equation:

$$L_{c, \pi-\pi}=\frac{2\pi K}{{\Delta q}_{020}}$$

where K is a shape constant, which is assumed to be 1 in present study, and ${\Delta q}_{020}$ is the FWHM of the PEDOT (020) peak. The shape constant was assumed to be 1.^[13,14]^ The paracrystallinity of π-π direction was also calculated with the following equation;

$$g_{\pi-\pi} (\%)=\sqrt{\frac{{\Delta q}_{020}}{2\pi\cdot q_{020}}}$$

where $q_{020}$ and ${\Delta q}_{020}$ are the position and FWHM of the (020) peak.^[13,14]^

*XPS characterization*

PHI - VersaProbe III was utilized for XPS characterization on PEDOT:PSS thin film samples. The X-ray source is Al Kα. The S_2p_ spectra (163~173eV) were used to calculate the PEDOT doping level, following the method reported by T. Liu *et al.*^[15]^ Thiophene signals and PSS signals were fitted by the Gaussian function to deconvolute the peak of PEDOT^0^ (center: 164.1 and 165.3eV), PEDOT^+^ (center: 164.6 and 166eV), PSS^-^ (167.2 and 168.7eV), and PSSH (167.8 and 169eV). The PEDOT doping level was determined by the peak area of PEDOT^+^ divided by the total area of both PEDOT^0^, and PEDOT^+^. PSS-to-PEDOT ratio was calculated as the area of (PSS^-^ + PSSH) divided by the area of (PEDOT^0^ + PEDOT^+^). An example of XPS fitting is shown in Supporting Information. (**Figure S21**)

*Statistical analysis*

To evaluate the correlation between PEDOT Raman spectra parameters (peak position and area), PEDOT crystalline structure (intermolecular spacing *d_π-π_*, crystal coherence length *L_c, π-π_*, and paracrystallinity *g_π-π_*), and PEDOT doping level, the Pearson correlation coefficient of each combination was calculated as follows:

$$\rho= \frac{\sum_{i=1}^{n} \left( x_{i}-\bar{x} \right)\left( y_{i}-\bar{y} \right)}{\sqrt{\sum_{i=1}^{n} \left( x_{i}-\bar{x} \right)^{2}}\sqrt{\sum_{i=1}^{n} \left( y_{i}-\bar{y} \right)^{2}}} (\boldsymbol{Eq. 5})$$

where n is the number of sample points, $x_{i}$ and $y_{i}$ is the x and y value of individual sample points, and $\bar{x}$ and $\bar{y}$ are the sample means. The Spearman rank correlation was also applied to study the correlations, calculated as follows:

$$r_{s}= \frac{\sum_{i=1}^{n} \left( {R[x}_{i}]-\bar{R[x]} \right)\left( {R[y}_{i}]-\bar{R[y]} \right)}{\sqrt{\sum_{i=1}^{n} \left( {R[x}_{i}]-\bar{R[x]} \right)^{2}}\sqrt{\sum_{i=1}^{n} \left( {R[y}_{i}]-\bar{R[y]} \right)^{2}}} (\boldsymbol{Eq. 5})$$

where n is the number of sample points, $R[x_{i}]$ and ${R[y}_{i}]$ is the rank of *x* and *y* value, and $\bar{R[x_{i}]}$ and $\bar{{R[y}_{i}]}$ are the means of ranking. The significance *p* from the “Regression analysis” was applied to verify the confidence in the correlation or reject the null-hypothesis that the scattered data has no correlation but random error. The “Regression analysis” was conducted with a built-in function in Microsoft Excel. Sample number is 1 sample per post-treatment condition (n = 1, 1 measurement per sample) for XPS characterization (Figure 1(a)(d)), 3 samples (n = 3, 1 measurement per sample) for GIWAXS measurement (Figure 1(b)(e), S2, and S4), and 3 samples (n = 3) for thermoelectric characterization (Figure 1(c)(f), and S6). Figure 2 and 3 show the average Raman spectra from 3 samples (n = 3, 18 measurements per sample) tested in GIWAXS measurement. The rest of UV-Vis-NIR (Figure S1) and Raman spectra (Figure S6-8, S10-S15 & S21) shown in Supporting Information correspond to 1 measurement from 1 sample (n = 1, 1 measurement per sample condition) for each condition.

Supporting Figures


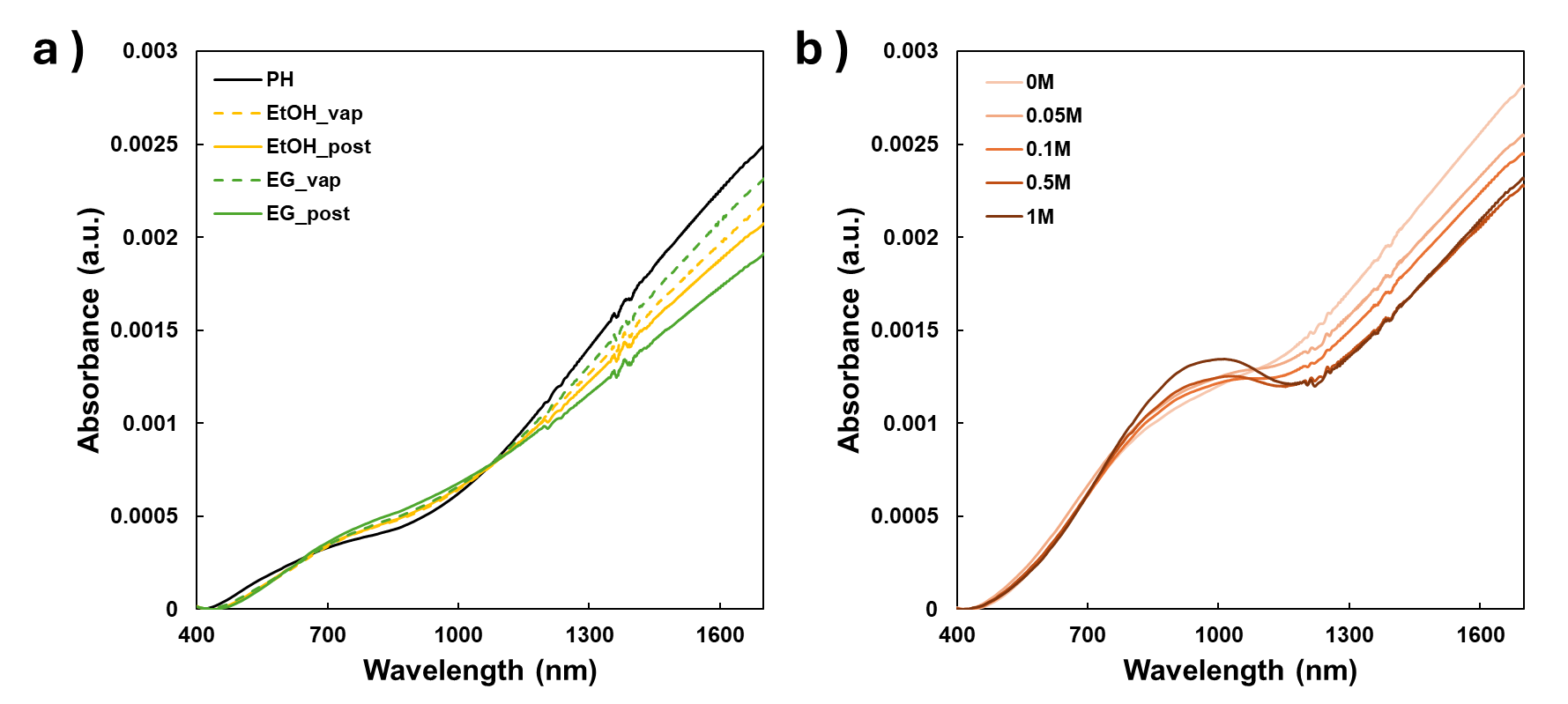


**Figure S1**. (a) UV-Vis-NIR absorption spectra of PSPT PEDOT:PSS samples. *PH* refers to pristine PEDOT:PSS (*PH1000*, *Clevios*). No significant change below wavelengths ~ 1300 nm was observed. (Single sample measurement n = 1) (b) UV-Vis-NIR absorption of Na_2_SO_3_-reduced PEDOT:PSS samples. (Single sample study n = 1). Although quantifying PEDOT doping with UV-vis-NIR is challenging, the reduction of the band spanning wavelengths > 1300 nm at the expense of increasing absorbance of the 700-1300 nm band has been associated previously with de-doping.^[16–18]^


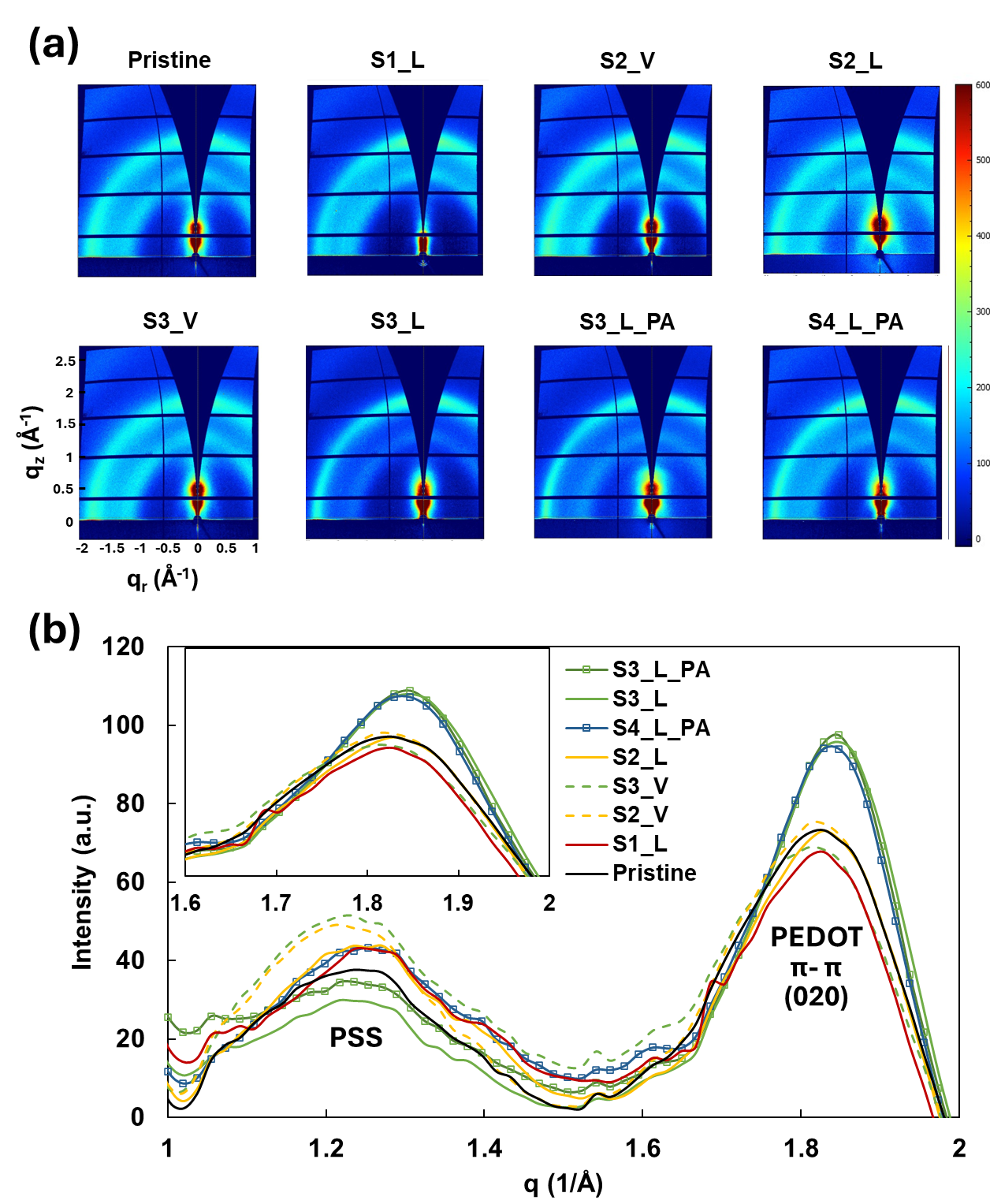


**Figure S2**. Example of (a) GIWAXS 2D patterns and (b) 1D line-cuts of PSPT PEDOT:PSS samples. (Single sample study n = 1). Liquid post-treatment with the most polar solvents, ethylene glycol (S3_L) and glycerol (S4_L), leads to the highest intensity ratio between PEDOT π-π stacking and PSS peaks, which is consistent with the PSS:PEDOT ratio measured with XPS (**Table 1**).


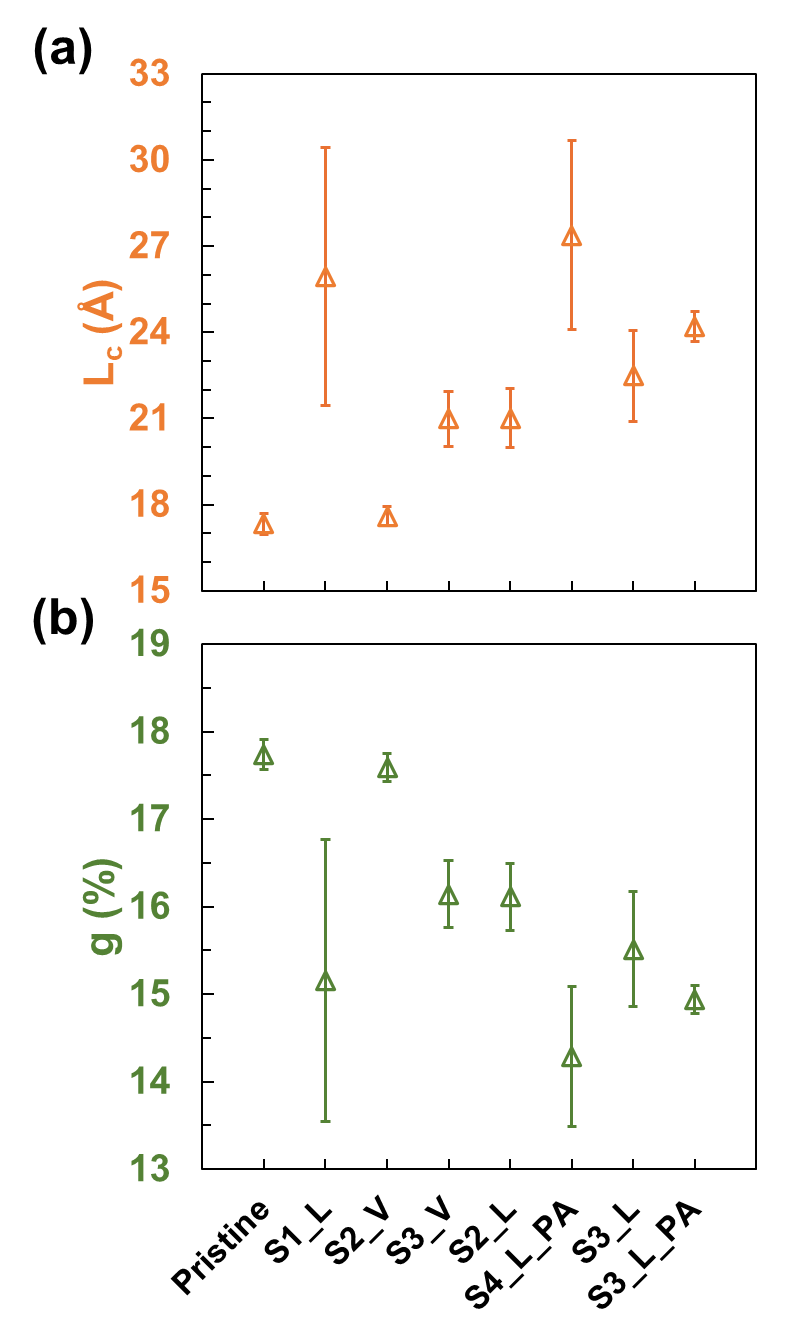


**Figure S3**. (a) π-π crystal coherence length, *L_c_*, and (b) paracrystallinity, *g*, of PSPT PEDOT:PSS samples ordered according to their increasing conductivity value (**Figure 1**). (Sample population n = 3, value: mean $\pm$ standard error). PSPT induced strong changes in those crystallinity features. However, a potential underlying trend between *L_c_* or *g* and conductivity is broken by S1_L (post-treatment with dioxane liquid) and S4_L_PA (post-treatment with glycerol liquid followed by post-annealing) samples. Thus, no universal trend can be claimed.


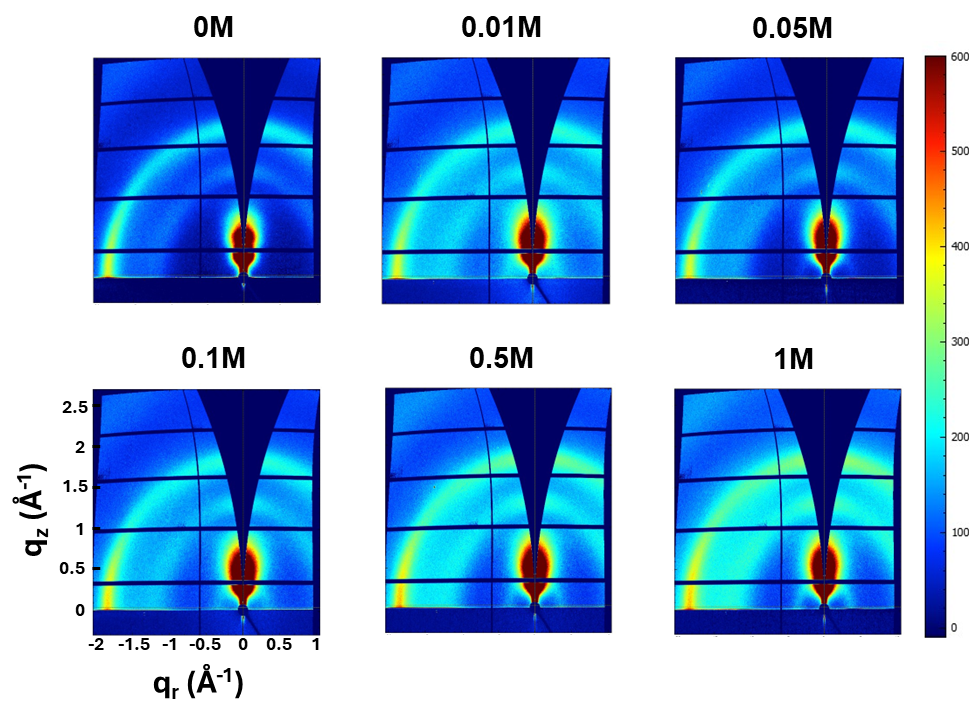


**Figure S4**. Example of GIWAXS 2D patterns of Na_2_SO_3_-reduced PEDOT:PSS samples. (Single sample study n = 1).


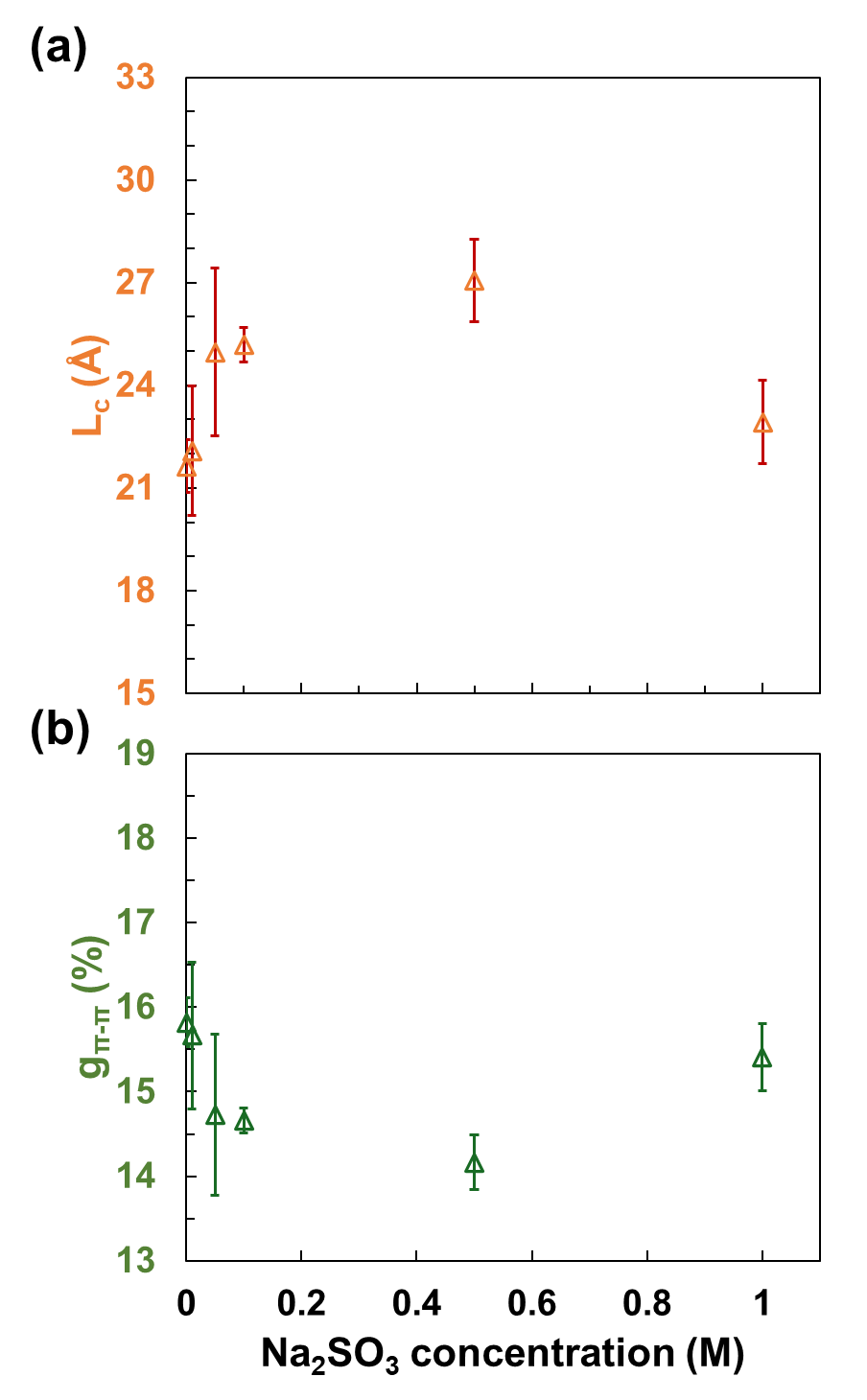


**Figure S5**. (a) π-π crystal coherence length, *L_c_*, and (b) paracrystallinity, *g*, of Na_2_SO_3_-reduced PEDOT:PSS samples post-treated at different Na_2_SO_3_ concentrations. (Sample population n = 3, value: mean $\pm$ standard error). Strong variations (compared to Figure S2) in these crystallinity features with Na_2_SO_3_ concentrations could not be found.


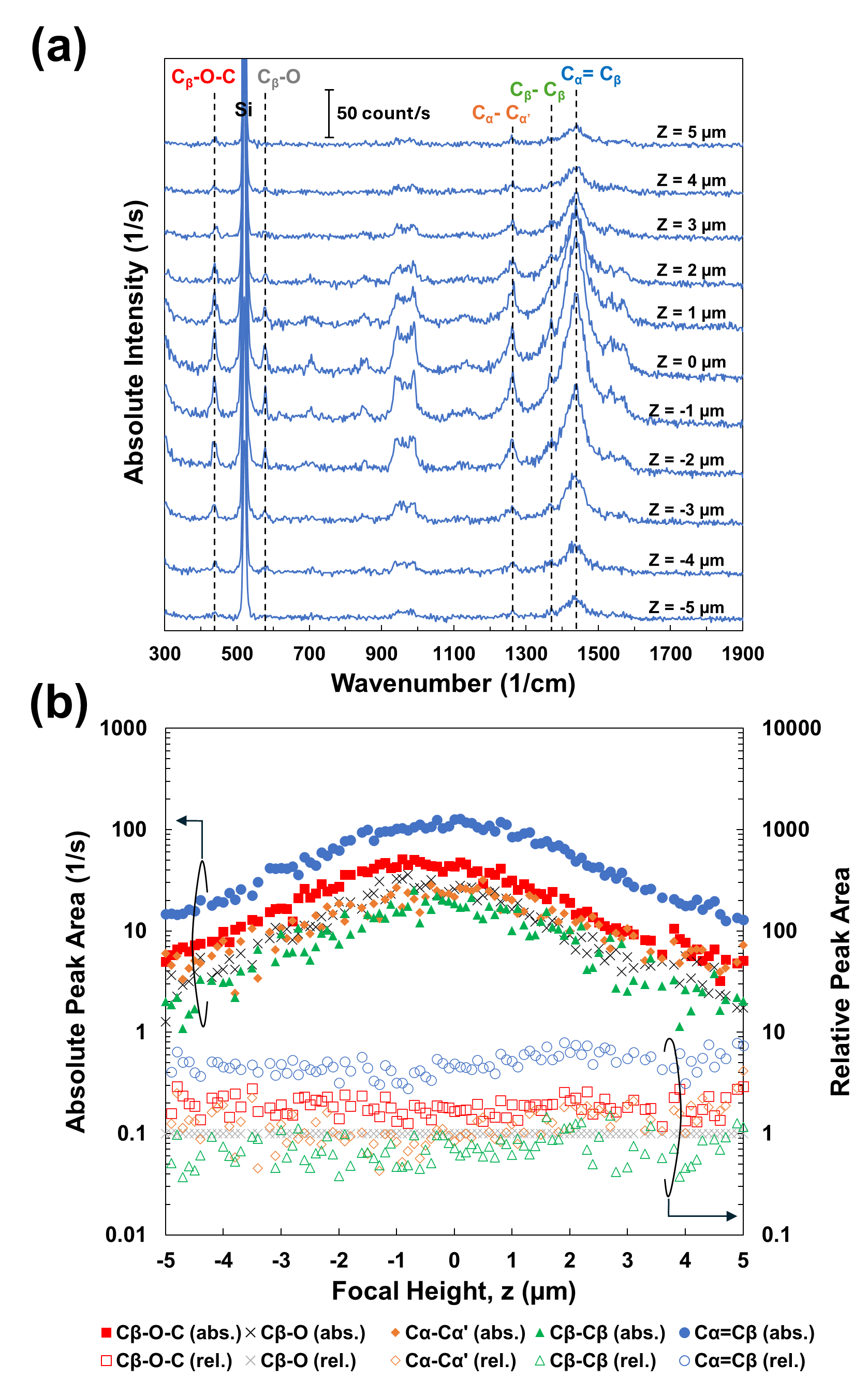


**Figure S6.** (a) Raman spectra of pristine PEDOT:PSS thin film at varied focal height, *Z*. (Single sample study n = 1). All the spectra were collected from the same spot at different focal heights. The focal height with the maximum Raman intensity is defined as *Z* =0. A negative focal height means the microscope focal plane is below the film surface, and a positive focal height means the microscope focal plane is above the film surface. (b) The fitted absolute and relative area of five peaks studied in the correlation analysis (**Table S2**) vs focal height. (n = 1) The relative peak area is calculated by normalizing the absolute intensity of each peak with that of C_β_-O peak area. While the absolute intensity (peak area) is significantly affected by varying focal height, peak normalization provides stable relative intensity.


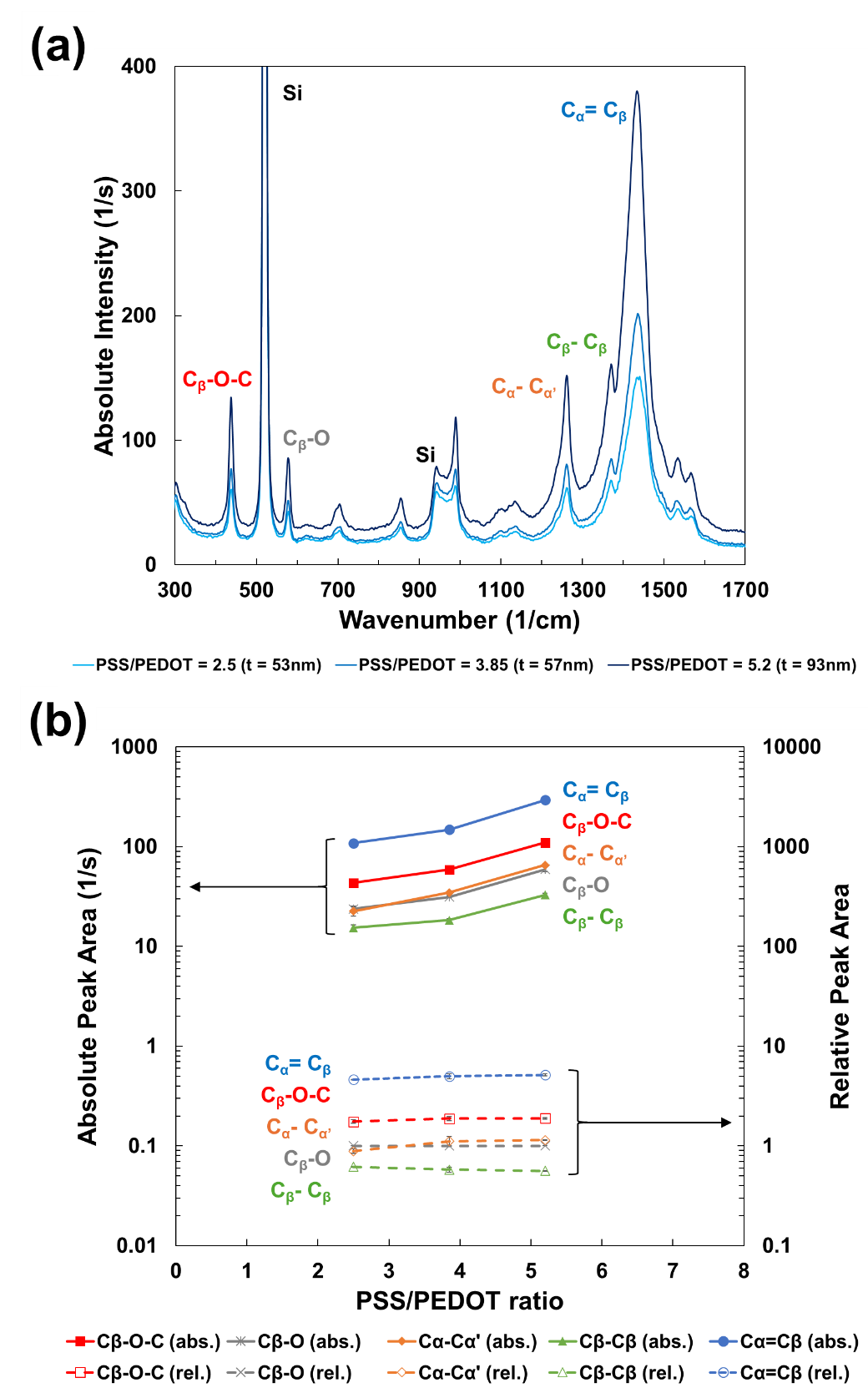


**Figure S7.** (a) Raman spectra of PEDOT:PSS with varied PSS/PEDOT ratio. (Sample population n = 2). The PSS/PEDOT ratio is controlled by adding extra NaPSS into pristine PEDOT:PSS suspension (PH1000), resulting PSS/PEDOT ratios = 2.5 (pristine PH1000), 3.85 (0.5wt% extra NaPSS in PH1000), and 5.2 (1wt% extra NaPSS in PH1000). Due to viscosity change, the thickness of PEDOT:PSS films was increased when extra NaPSS was added. All the films were prepared by spin-coating at 1000 rpm for 2 minutes on a silicon substrate and probed by a 1.7 mW 633 nm HeNe laser. (b) The fitted absolute and relative area of five peaks studied in the correlation analysis (**Table S2**) vs PSS/PEDOT ratio. (n = 2) The relative peak area is calculated by normalizing the absolute intensity of each peak with that of the C_β_-O peak area. The absolute peak area is affected by PSS/PEDOT ratio and film thickness, while the relative peak area remains largely unchanged.


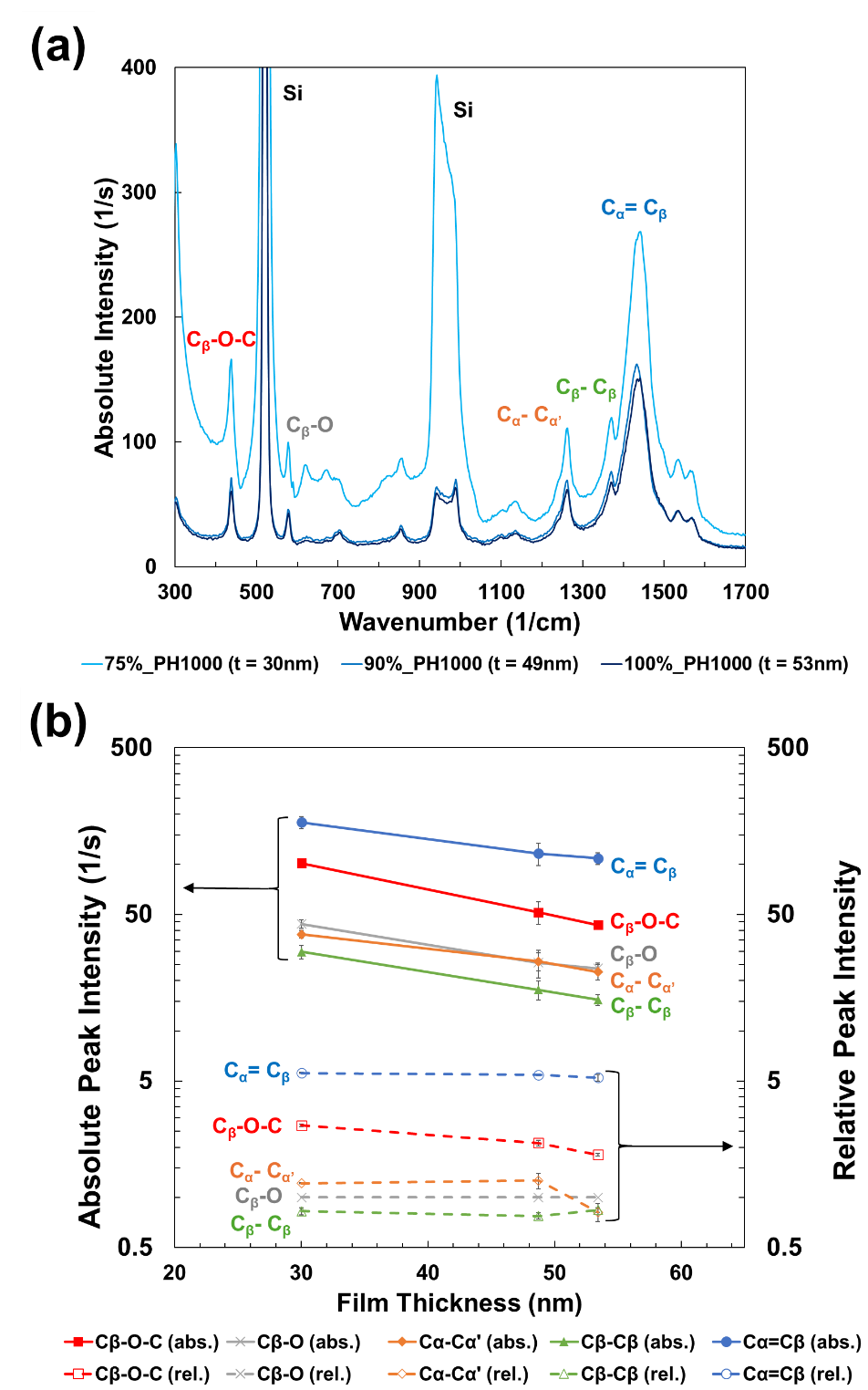


**Figure S8.** (a) Raman spectra of PEDOT:PSS with varied film thickness. (Sample population n = 2). The PSS/PEDOT ratio is controlled by diluting PEDOT:PSS suspension (PH1000) with DI water, resulting in film thickness from 30 nm (75% PH1000) to 53 nm (100% PH1000). Rather than a thicker film, a thinner film shows a stronger absolute Raman intensity, and the possible reason could be attributed to the back reflection of the probing laser at the PEDOT:PSS/Si substrate interface. All the films were prepared by spin-coating at 1000 rpm for 2 minutes on a silicon substrate and probed by a 1.7mW 633 nm HeNe laser. (b) The fitted absolute and relative peak area of five peaks studied in the correlation analysis (**Table S2**) vs film thickness. (n = 2) The relative peak area is calculated by normalizing the absolute intensity of each peak with that of the C_β_-O peak area. The absolute peak area is affected by film thickness while the relative peak area remains largely unchanged.


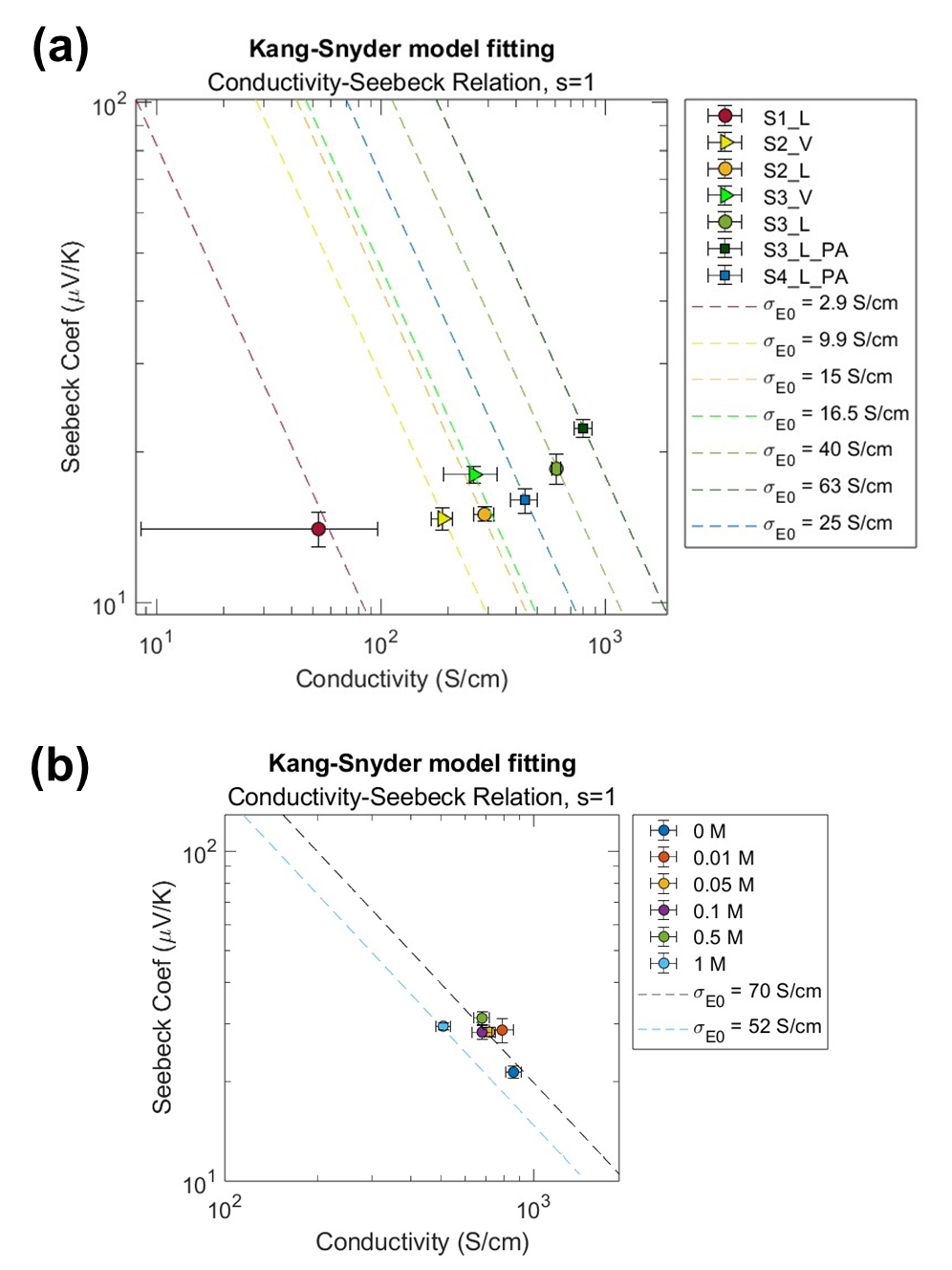


**Figure S9**. Kang-Snyder model fitting of (a) PSPT and (b) Na_2_SO_3_-reduced PEDOT:PSS thin film samples. For all fitting, s = 1. (Sample population for each condition n = 3. Value: mean $\pm$ standard error). The dashed lines correspond to different transport coefficient values σ_E0_ (related to mobility), and different points along the same dashed line represent samples with the same mobility but different charge carrier concentration. It was impossible to fit all samples of the PSPT series with the same line, indicating that they have different mobility values. On the contrary, we could fit relatively well all samples except 1M of the Na_2_SO_3_ reduced series on the same dashed line, indicating that the difference between samples is not mobility but the doping level. Note that for the values in Table S1, no assumption was made, and each sample has been fitted with a different σ_E0_.


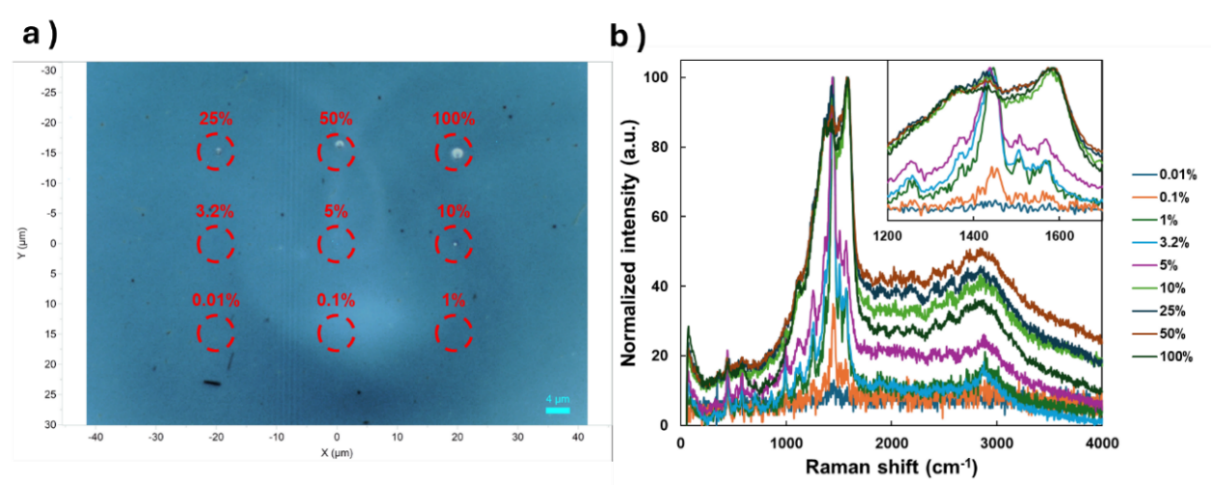


**Figure S10**. (a) Optical microscope image and (b) Raman spectra of PEDOT:PSS probed by a 532nm laser for 1 second (frequency-doubled Nd:YAG laser with 100mW max power) to screen out the optimized measurement condition without damaging samples. (Single sample study n = 1). Laser damage can be recognized by 1) a burned sample at the laser spot in panel (a), and 2) a smeared Raman signal in panel (b). For example, spectra probed by laser above 10% of max power are severely smeared.


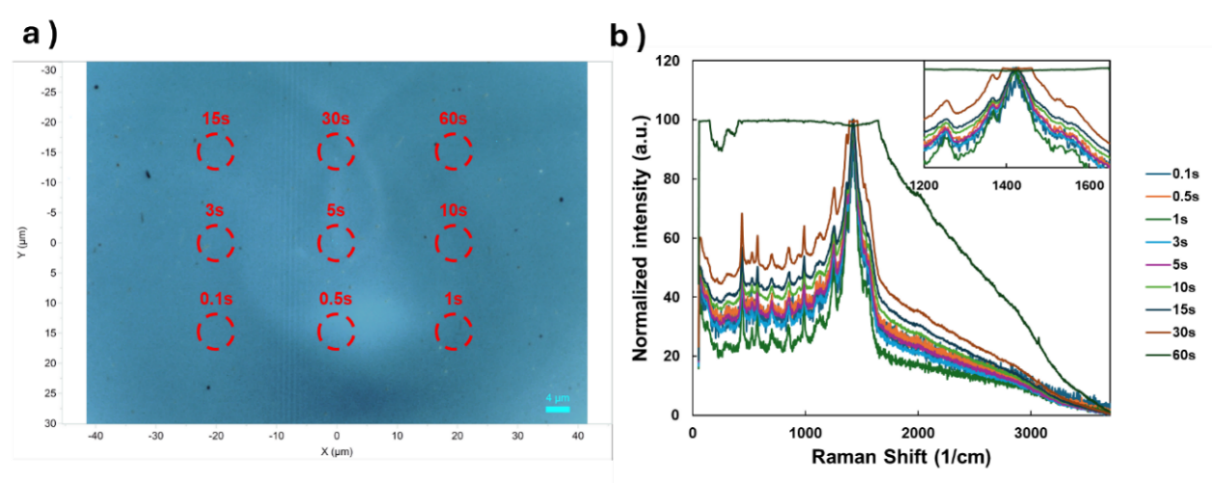


**Figure S11**. (a) Optical microscope image and (b) Raman spectra of PEDOT:PSS probed by a 633nm laser with 100% power (HeNe laser with 17mW max power) to screen out the optimized measurement condition without damaging samples. (Single sample study n = 1). No burn spot is observed in panel (a), even after exposure for a few seconds at the max laser power, and all spectra in panel (b) are well defined, except the signal from the sample exposed for 60s, which is saturated. Even though laser damage has not been identified, excessive laser exposure can modify the Raman signature as indicated in **Figure S13**.


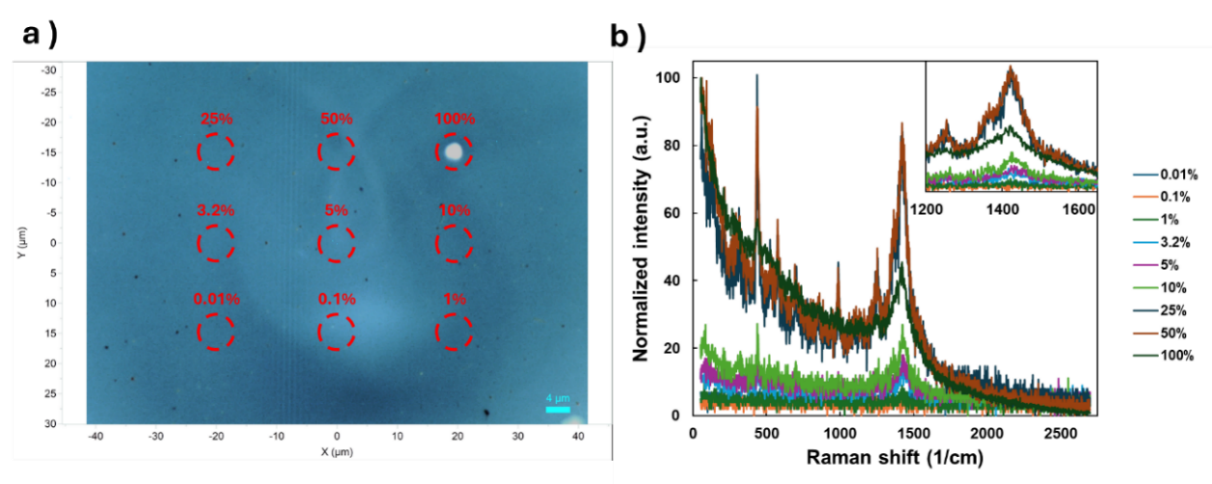


**Figure S12**. (a) Optical microscope image and (b) Raman spectra of PEDOT:PSS probed by a 785nm laser for 1 second (intracavity regulated, diode laser with 100mW max power) to screen out the optimized measurement condition without damaging samples. (Single sample study n = 1). Laser damage can be recognized by a burned sample at the laser spot in panel (a) when the sample is exposed to 100% power.


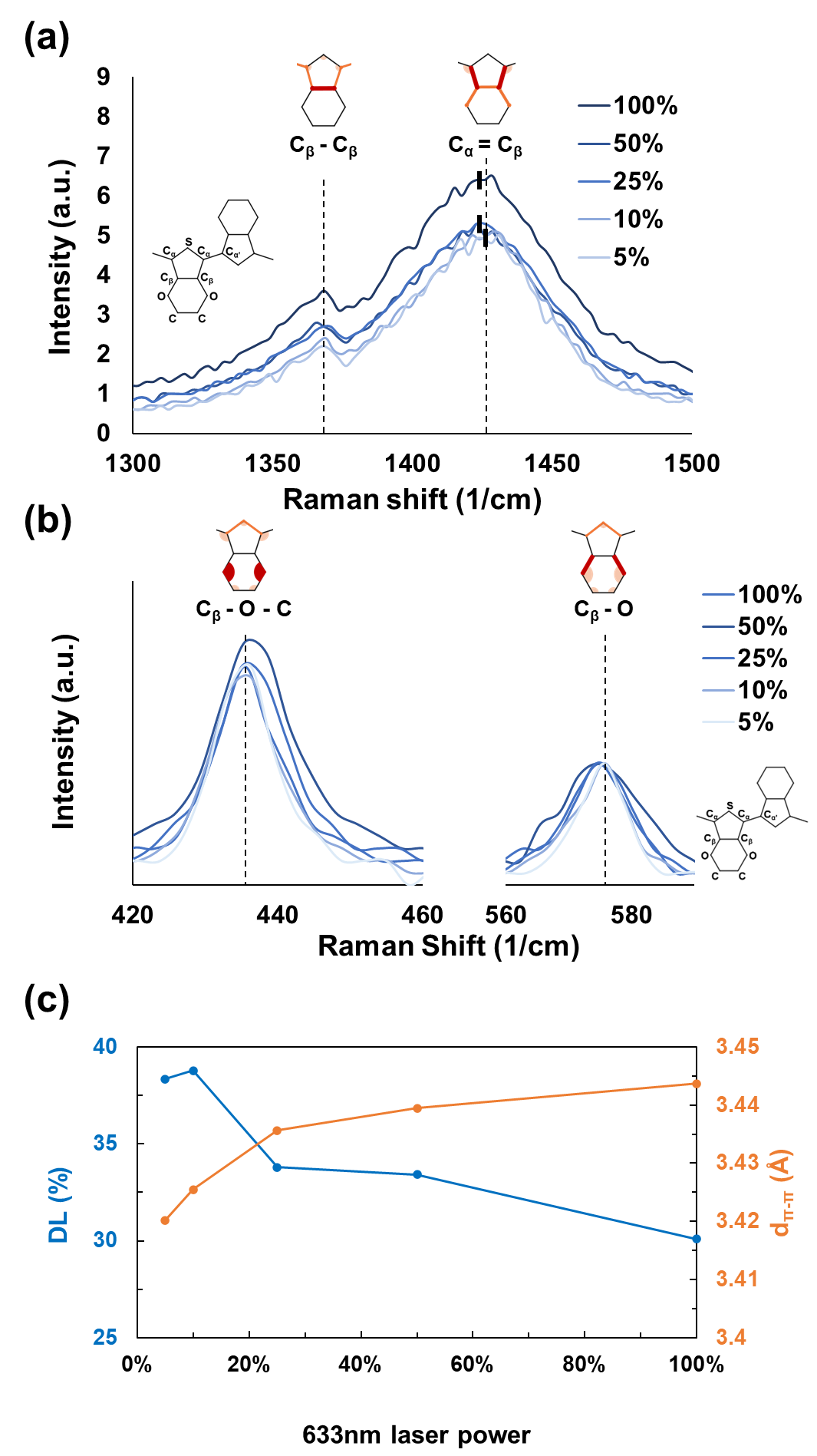


**Figure S13**. Raman spectra of pristine PEDOT:PSS drop-casted film collected with different 633 nm wavelength HeNe laser power levels (max power: 17mW, exposure time: 3 seconds). (a) Wavenumber ranging from 1200 to 1700 cm^-1^: when the laser power exceeds 10% of its maximum power, the C_α_=C_β_ peak significantly shifts, implying that excessive laser exposure induces morphology/doping level changes.^[8,19–21]^ (b) Wavenumber ranging from 400 to 600 cm^-1^, and (c) estimated DL and d_π-π_ of drop-casted pristine PEDOT:PSS film from the C_β_-O-C and C_β_-O peaks in **Figure S11(b)** using **eq. 1** and **2**. (Single sample study n = 1). According to **Table 2**, the shifting and area change of the C_β_-O-C and C_β_-O peaks upon excessive laser exposure are consistent with de-doping and an increase in the *π-π* stacking distance (*d_π-π_*). All spectra are fitted, background-corrected, and normalized by the C_β_-O peak area, as described in the Experimental Method section. 10% of the maximum power was selected for the experiments in this paper.


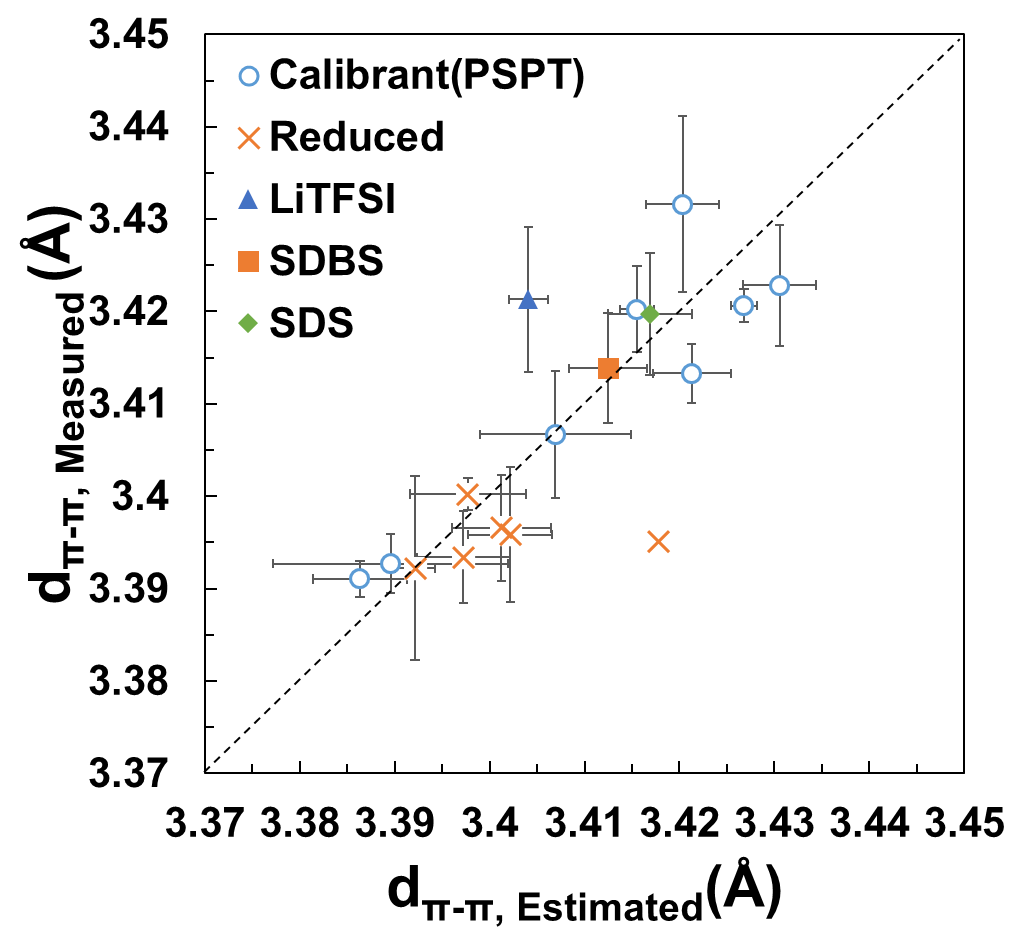


**Figure S14**. Comparison between the average PEDOT π-π intermolecular spacing (d_π_-_π_) measured from GIWAXS, and the estimated d_π-π_ from PEDOT Raman spectra with **Equation 1** for each tested condition (sample population for each condition n = 3; values: mean $\pm$ standard error).


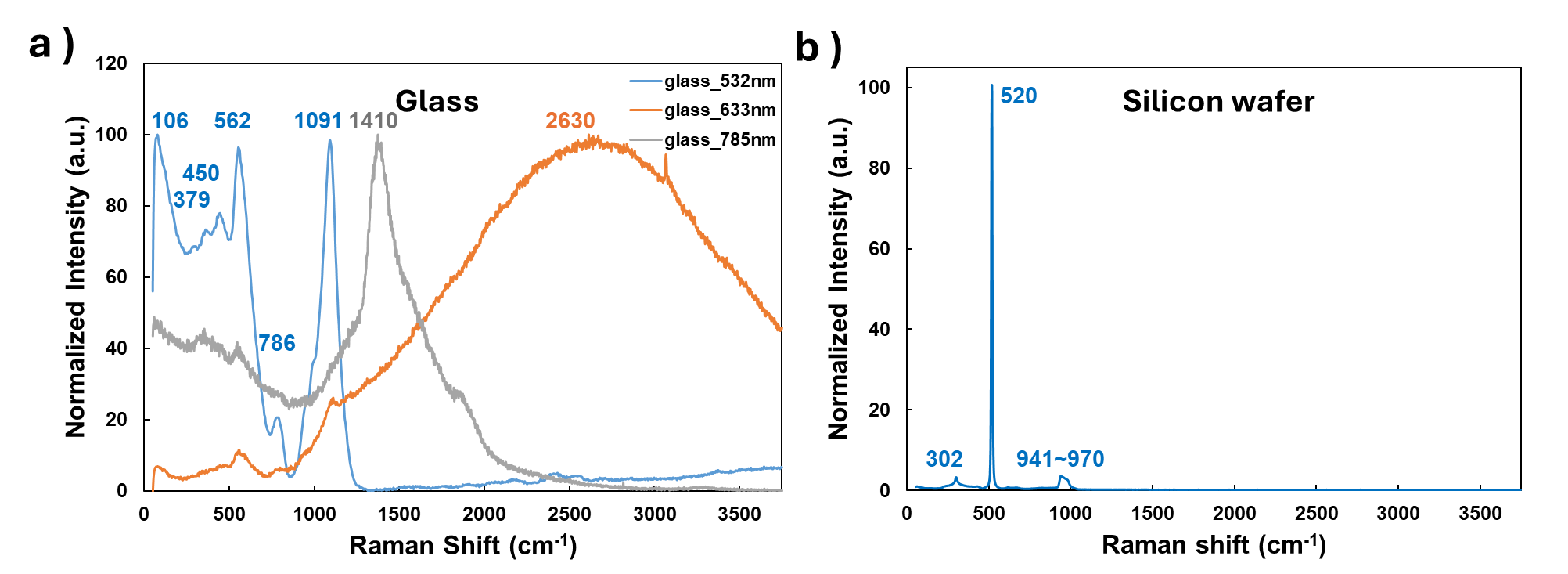


**Figure S15**. Raman spectra of (a) glass (for 3 different laser wavelengths) and (b) silicon substrates. (Single sample study n = 1) The Raman spectrum of the silicon substrate was probed only with a 633 nm wavelength HeNe laser (max power: 17mW) at 100% power for 5 seconds. All the spectra are normalized by the maximum intensity value. Using a 633 nm wavelength HeNe laser can prevent the overlapping between the PEDOT and the glass Raman spectra. The broad background of the glass in the Raman spectra probed by a 633 nm laser can be fitted and removed afterwards (Experimental Methods). The Raman signal from the silicon substrates is consistent with the literature.^[10]^ Therefore, the peak at 520 cm^-1^ serves as a reference for offset during peak fitting of the samples prepared on silicon substrates (GIWAXS and XPS samples).


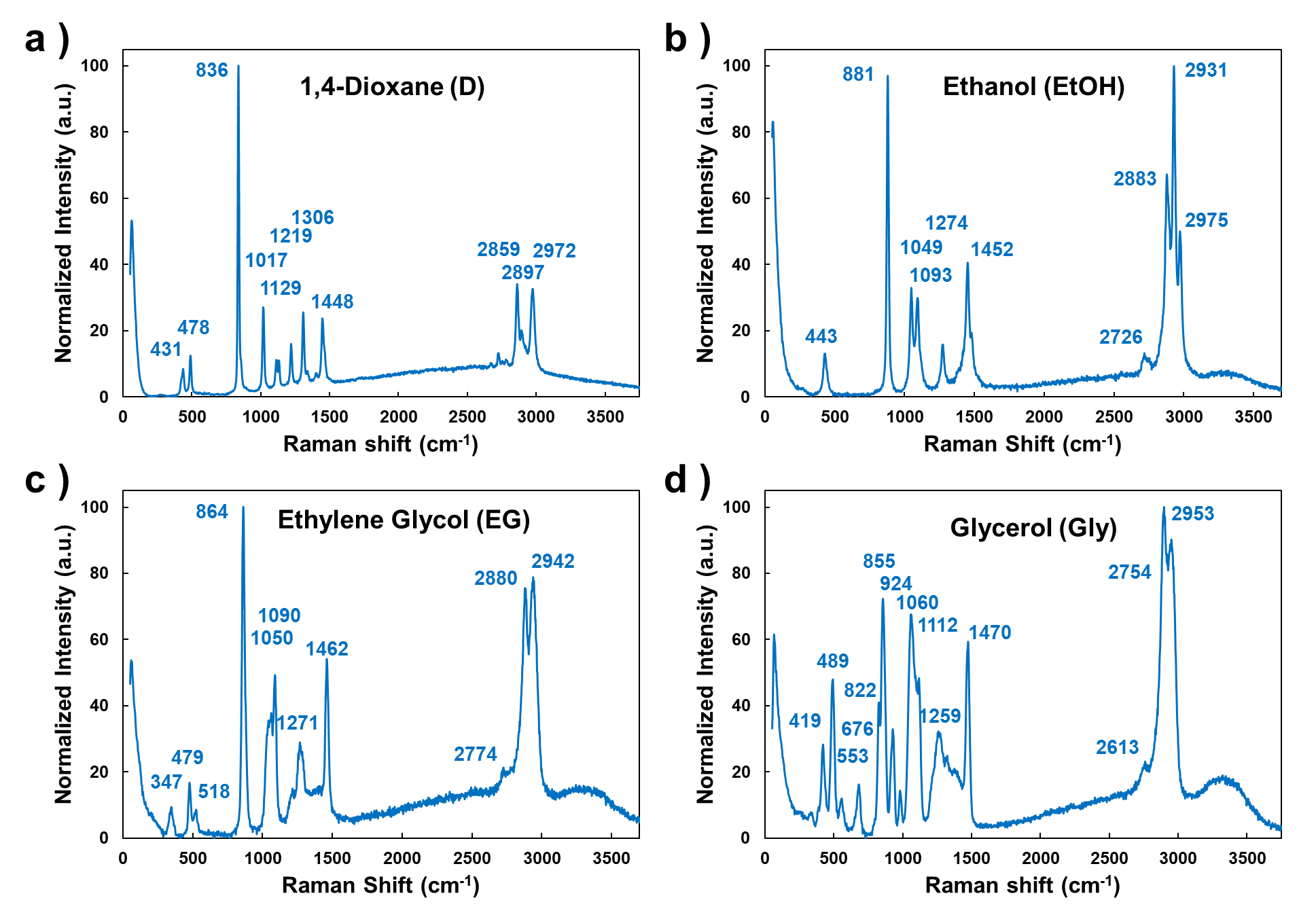


**Figure S16**. Raman spectra of (a) dioxane, (b) ethanol, (c) ethylene glycol, and (d) glycerol used for post-treatment (single sample study n = 1). These Raman spectra were probed with a 633nm HeNe laser (max power: 17mW) at 100% power for 10 seconds on pure liquid solvent. All spectra are normalized by the maximum intensity. The characteristic peaks of each solvent (especially the strongest peak and C-H vibrational peaks) are used to ensure that no residual solvents remain inside the PEDOT:PSS samples post-treated with polar solvents.


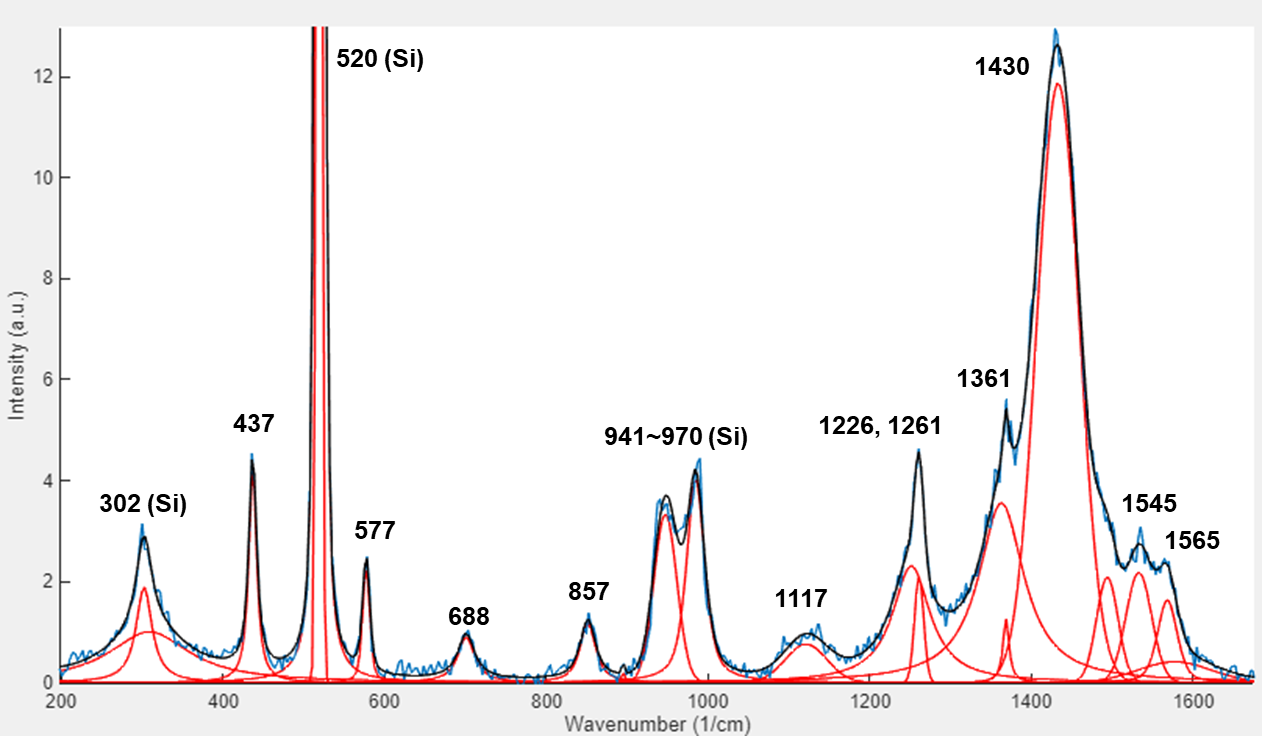


**Figure S17**. A fitting example of PEDOT:PSS Raman spectrum (pristine PEDOT:PSS thin film) (single sample study n = 1). The fitting procedure, peak form, and peak position of PEDOT signals follow the reported method.^[8]^


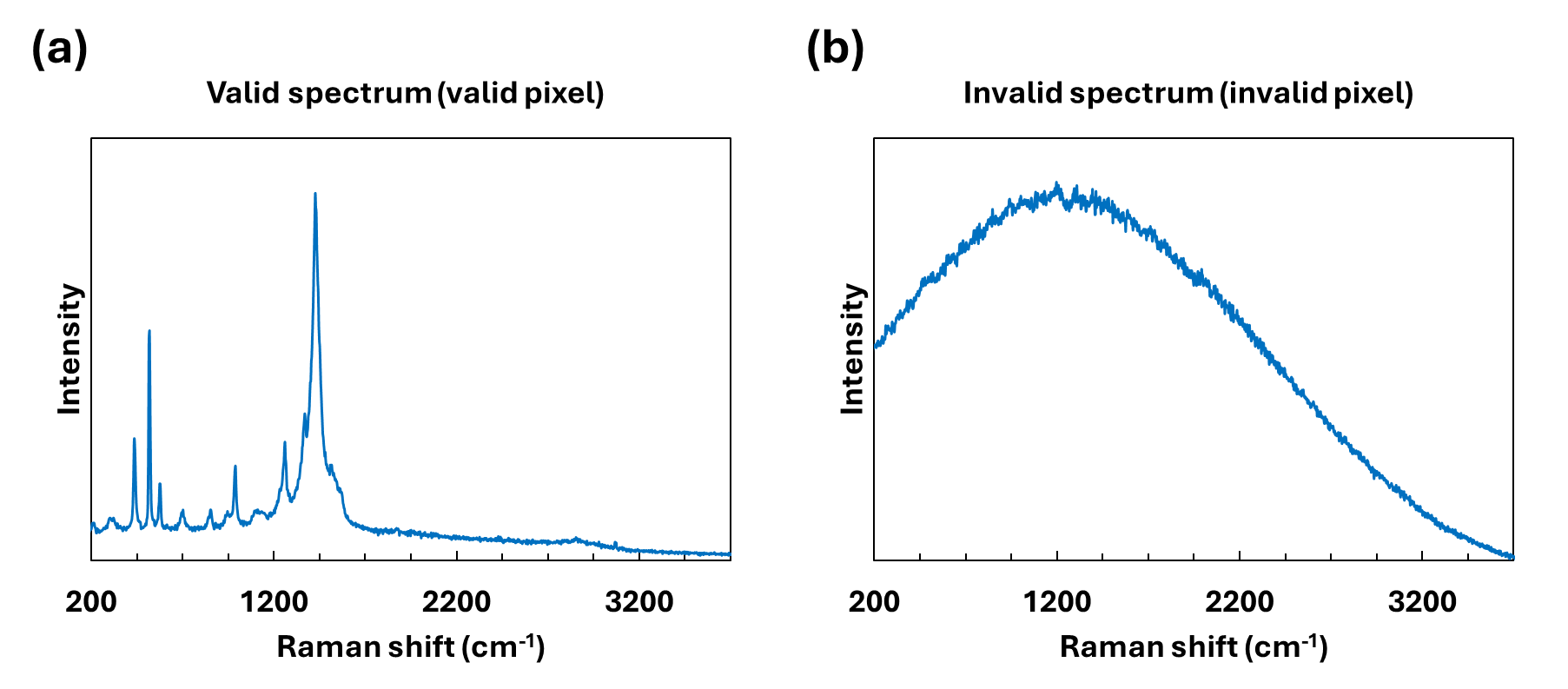


**Figure S18**. Representative Raman spectrum of (a) a valid pixel and (b) an invalid pixel for the mapping in Figure S19. (Single sample study n = 1). The strong background signal shown in the invalid spectrum resulted from the precipitated Na_2_SO_3_ after ink-jet printing 10 layers of Na_2_SO_3_ solution for local post-treatment.


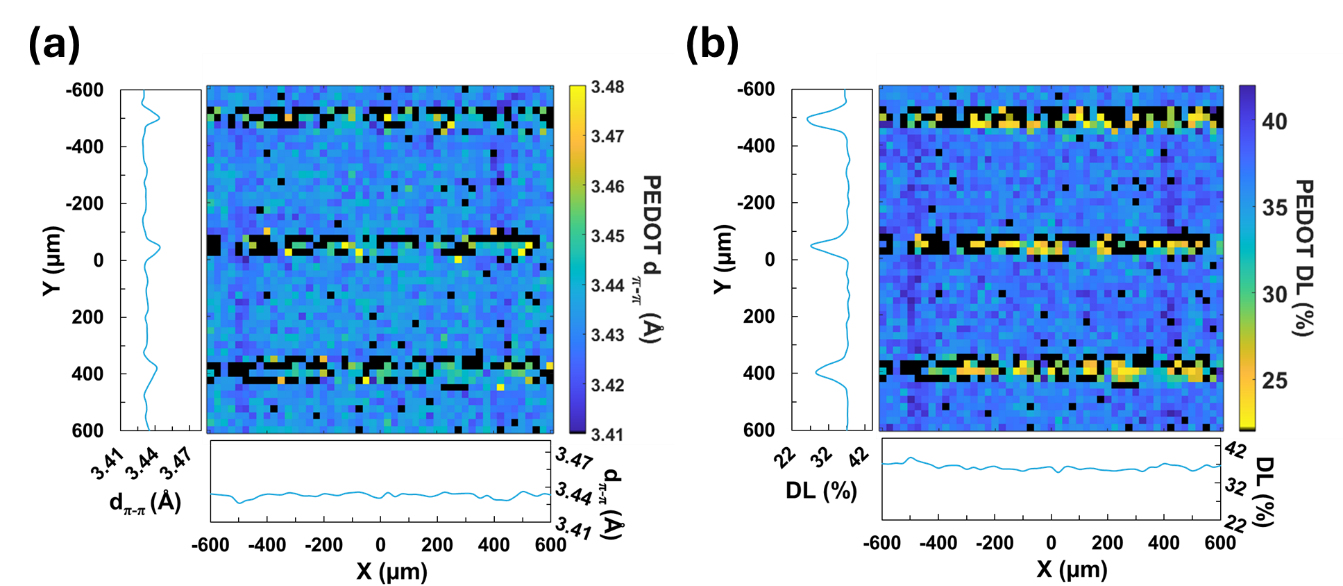


**Figure S19**. (a) PEDOT d_π-π_ and (b) doping level mapping extracted from Raman for samples locally treated by IJP without numerically filling invalid pixels by linear interpolation. (Single sample study n = 1). The black pixels result from the strong background signal from Na_2_SO_3_ precipitation, as shown in **Figure S18**b.


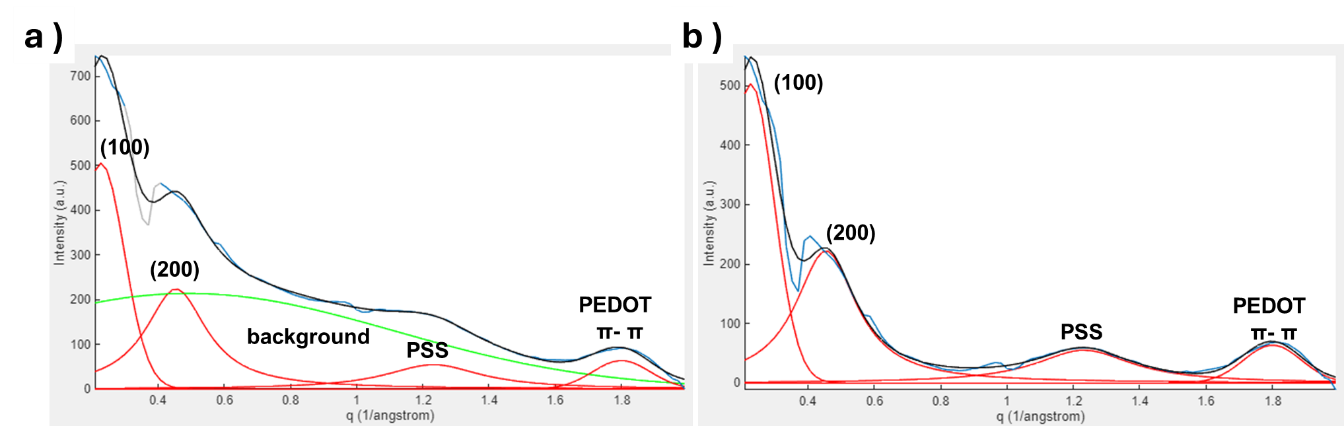


**Figure S20**. Fitting example of PEDOT:PSS 1D GIWAXS line-cut (Pristine PEDOT:PSS thin film) (a) before background subtraction. (b) after background subtraction. (Single sample study n = 1). The fitting procedure follows the one reported recently.^[12]^


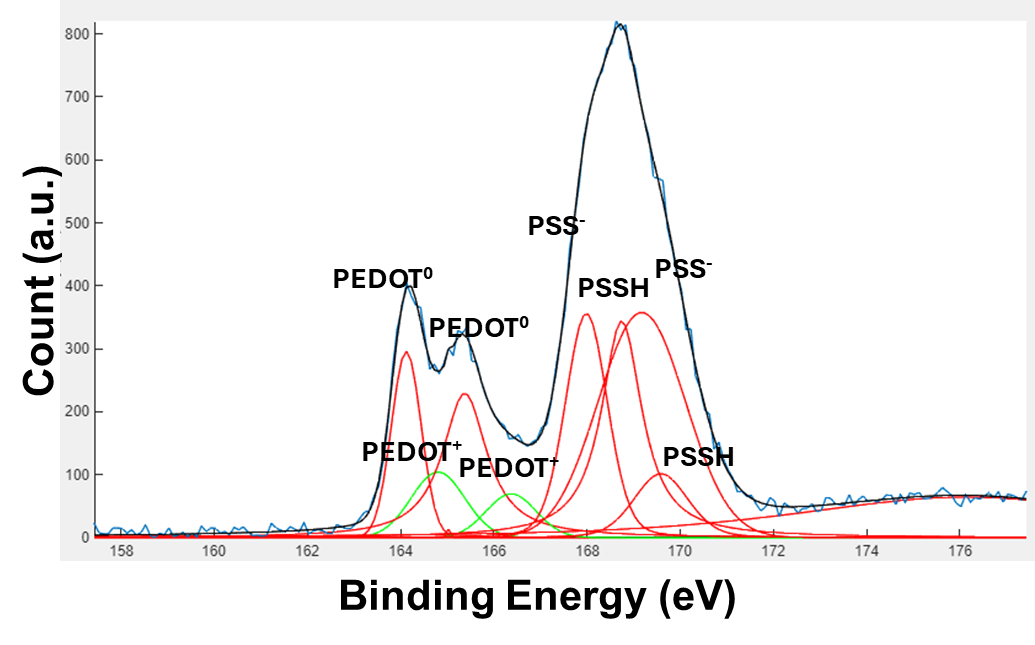


**Figure S21**. A fitting example of PEDOT:PSS XPS spectrum (Pristine PEDOT:PSS thin film). (Single sample study n = 1). The fitting procedure follows the literature.^[15]^ PEDOT doping level is calculated by the area of peaks labeled in green (PEDOT^+^) divided by total PEDOT area (PEDOT^+^+PEDOT^0^).


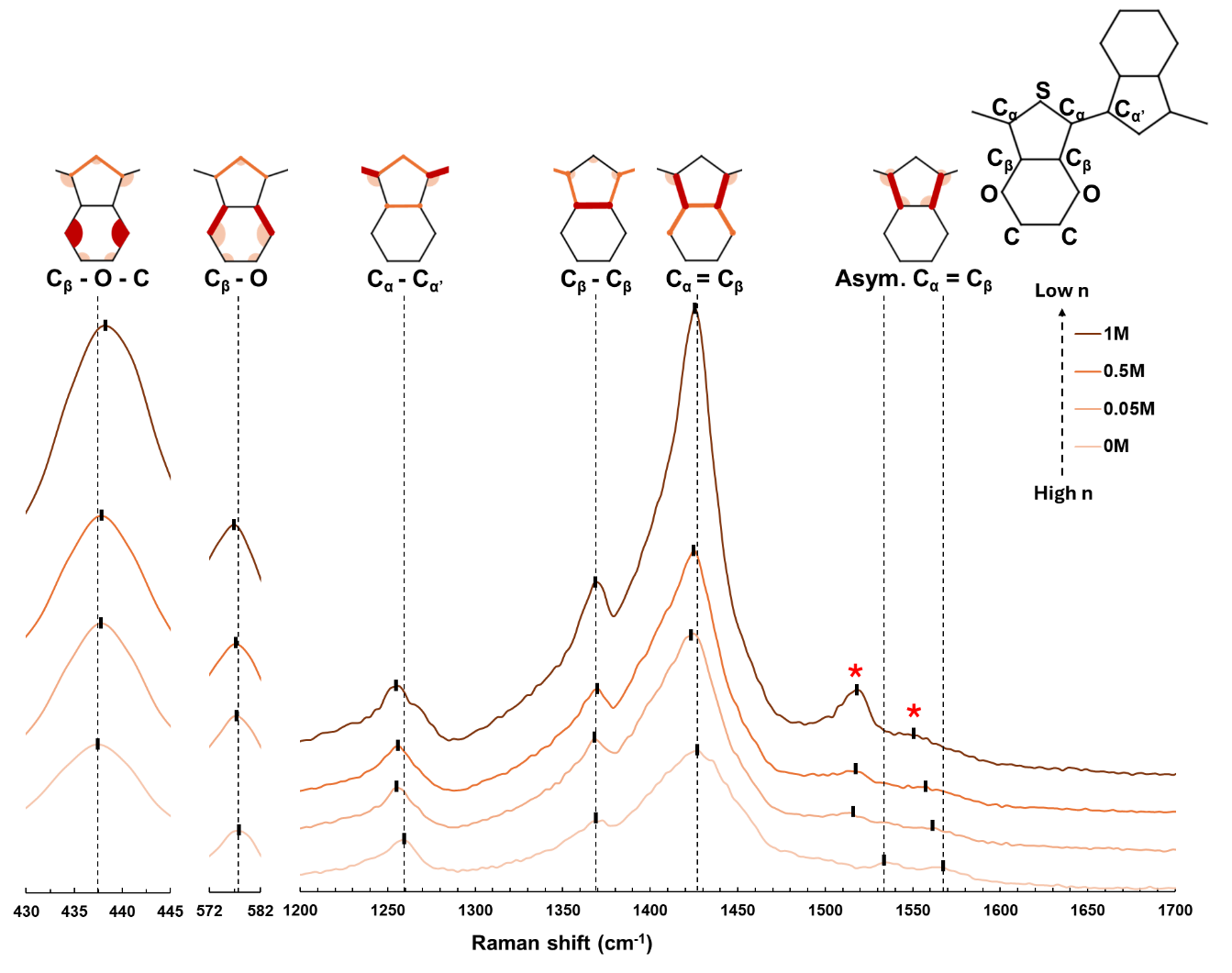


**Figure S22**. Raman spectra of the Na_2_SO_3_-reduced PEDOT:PSS thin film samples used for XPS measurements (**Table 1**). Each presented spectrum is the average of 3 replicate samples on silicon substrates. All spectra are offset with the reported silicon Raman peak (520 cm^-1^) and normalized by the C_β_-O peak area. The background signal is subtracted. The contributing vibrational modes (sector: scissoring and rocking; line: stretching) of each peak are illustrated in EDOT molecular structure. Peaks are named by their most contributing vibrational bonding (noted in dark red). ^[8,16]^ This Figure is different from Figure 3 because instead of showing the spectra for all the de-doped samples, it focuses on those particular samples probed with XPS and used to calculate the correlation between doping level and Raman features in **Table S2** below.

- Supporting Tables

**Table S1**. XPS, GIWAXS, and *K-S* model fitting results of PSPT and Na_2_SO_3_-reduced samples.
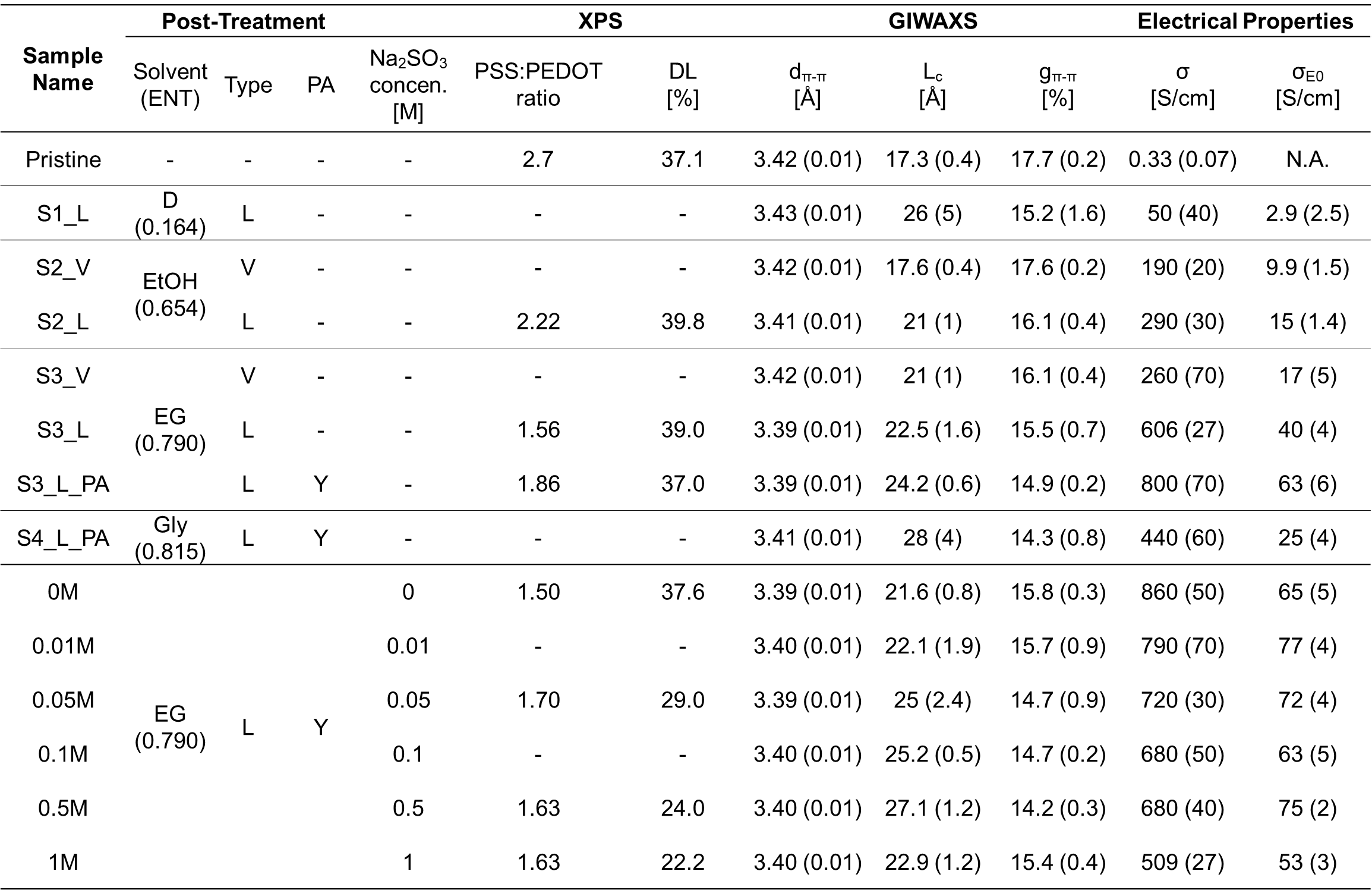


D = dioxane; EtOH = ethanol; EG = ethylene glycol; Gly =: glycerol; ENT = empirical solvent polarity parameter^[22]^; V = vapor post-treatment; L = liquid post-treatment; PA = post-thermal annealed at 120 °C for 10 minutes; DL= PEDOT doping level [%]. Value: mean (standard error).

**Table S2**. Correlation heat map between Raman peaks, crystalline parameters, and doping level. A significant correlation is considered when both Spearman and Pearson coefficients are > 0.5 and p-value < 0.1 (90% confidence). The significant correlations are summarized in Table 2.


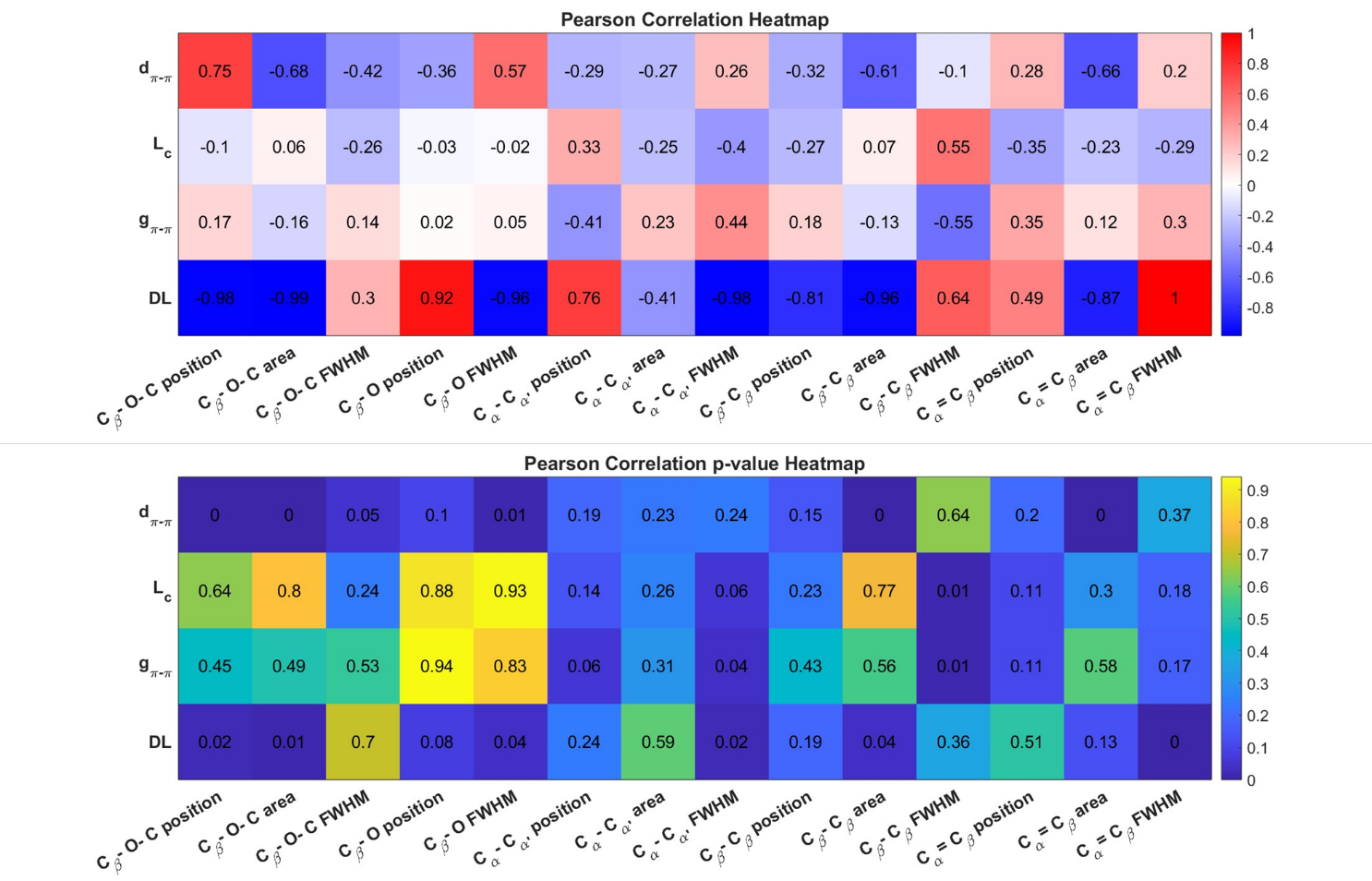

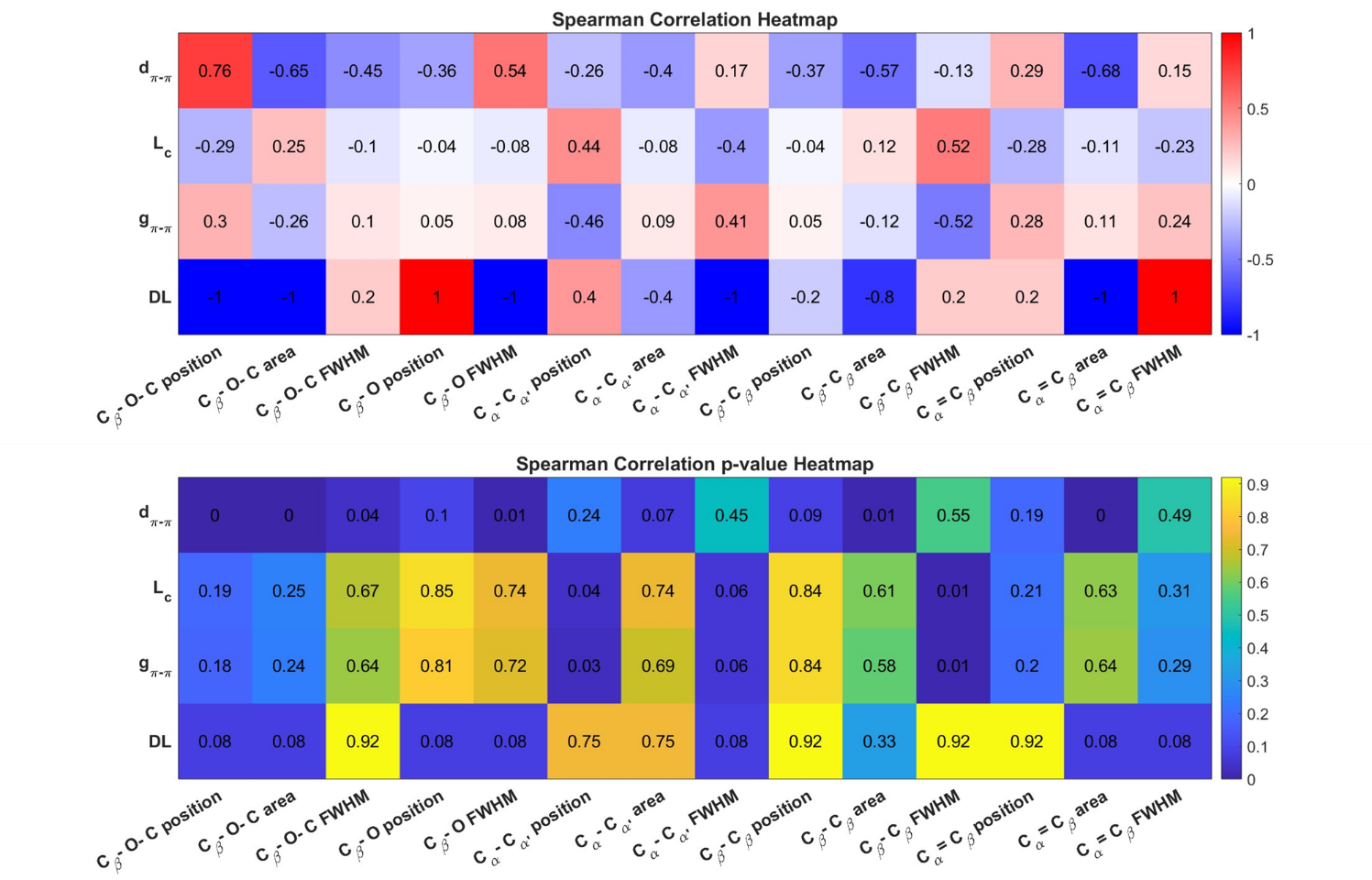


**Table S3**. XPS and GIWAXS results of additive-containing and strongly reduced PEDOT:PSS thin film samples.


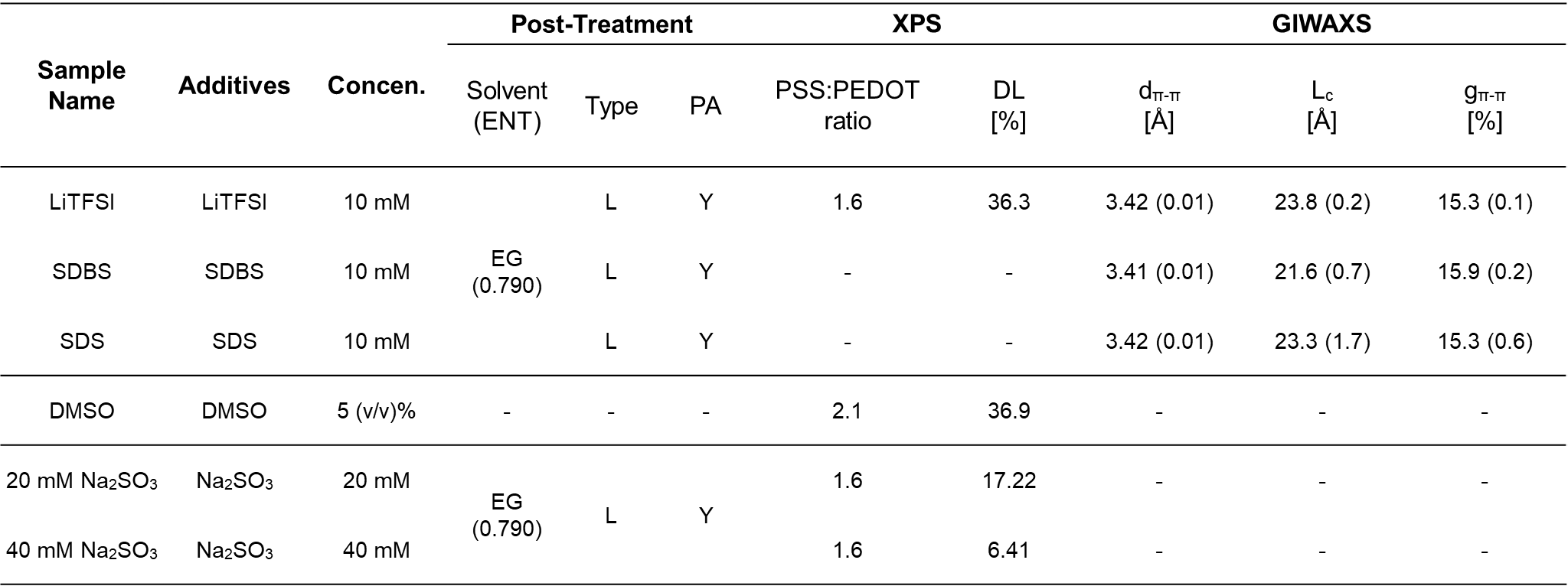


LiTFSI = lithium bis(trifluoromethanesulfonyl)imide; SDBS = sodium dodecylbenzenesulfonate; SDS = sodium dodecylsulfonate; DMSO = dimethylsulfoxide; EG = ethylene glycol; ENT = empirical solvent polarity parameter^[23]^; L= liquid post-treatment; PA = post-thermal annealed at 120 °C for 10 minutes; DL= PEDOT doping level [%]. Value: mean (standard error).

- **Derivation of empirical equations to evaluate PEDOT *d_π-π_* and doping level**

Based on the correlation heat map of PSPT and Na_2_SO_3_-reduced PEDOT PSS samples, the empirical equations for evaluating PEDOT π-π spacing (*d_π-π_*) and doping level (*DL*) were derived based on the position and area of C_β_-O-C Raman peak. In PEDOT:PSS Raman spectra, the C_β_-O-C and C_β_-O peaks are isolated and sharp, which can be fitted easily. Considering the absolute Raman peak shift reported in this study is exclusive for a Si substrate, and it will not be the same for free-standing samples and those supported by substrates other than silicon, the Raman shift difference between the C_β_-O-C peak and C_β_-O peak ($\Delta\upsilon_{C_{\beta}-O-C}- {\Delta\upsilon}_{C_{\beta}-O}$, represented as *ΔR* in this section), is chosen as a variable in the equation to generalize it to any PEDOT:PSS-based sample. Furthermore, the C_β_-O-C peak area is normalized by the C_β_-O peak area (A_Cβ-O-C_ /A_Cβ-O_, noted as *A’*) to compensate for factors affecting Raman scattering intensities, such as laser power fluctuations, instrument variation, PEDOT-PSS ratio difference, and film thickness. Unsurprisingly, since the peaks used to extract the variables *ΔR* and *A’* are correlated with *d_π-π_* and *DL* (**Table S2**), so are the variables themselves, as shown in **Figure S23**a-d. All the correlations exhibit a determination coefficient R^2^ > 0.5 and p-value < 0.1, demonstrating strong correlations among these parameters.


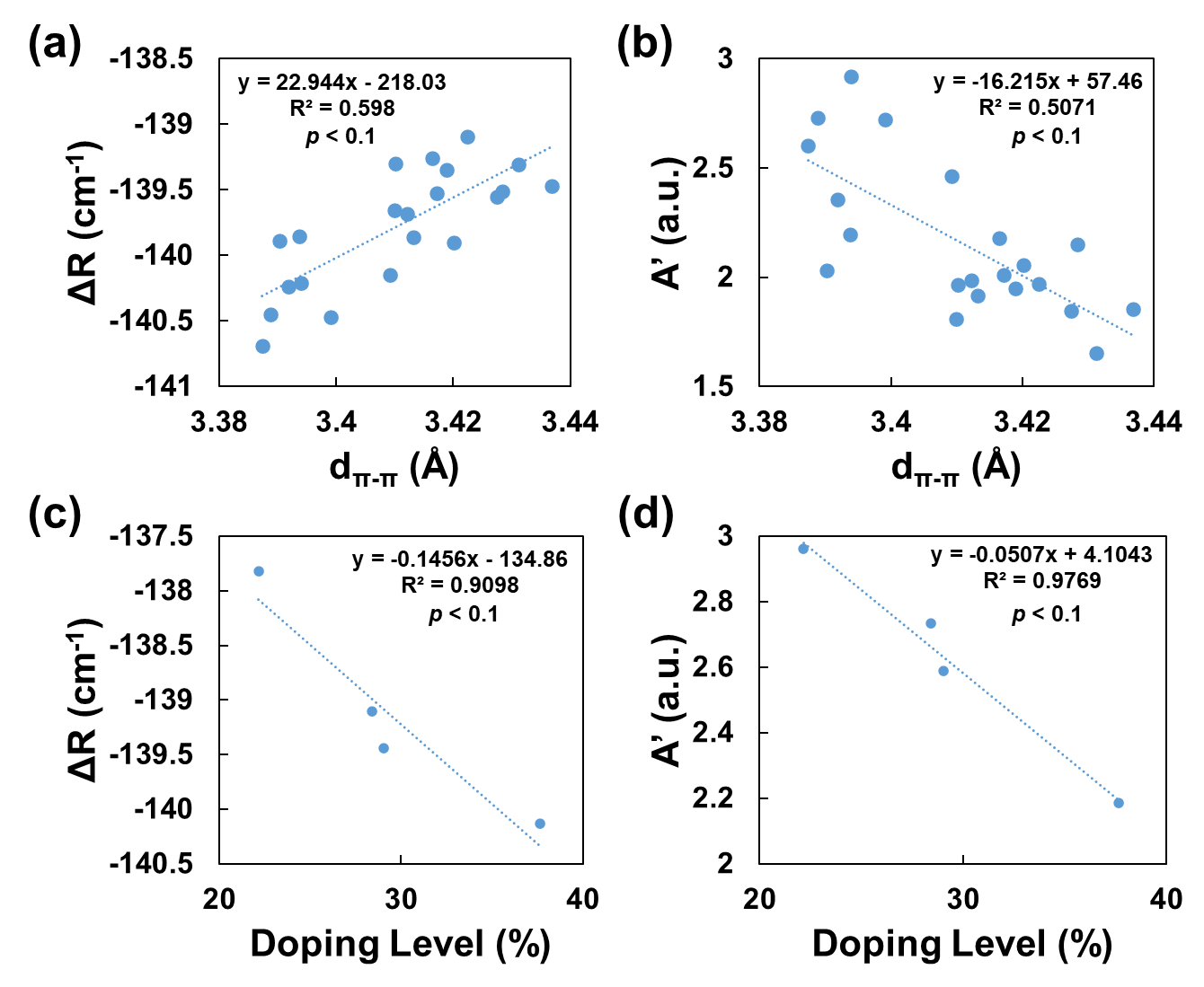


**Figure S23**. Correlation of **eq. 1** & **2** variables, ΔR and A’, with (a), (b) π-π spacing (PSPT samples), and (c), (d) doping level (Na_2_SO_3_-reduced PEDOT samples) The p-value of the presented regressions are all below 0.1, indicating a level of confidence above 95%.

To derive empirical equations evaluating *d_π-π_* and *DL* from Raman parameters, *ΔR* and *A’* are first described as functions of *d_π-π_* and *DL*, as follows

$$\left\{ \begin{aligned} \Delta R= \Delta R\left( d_{\pi-\pi}, DL \right) \{{cm}^{-1}\} \\ A^{'}= A^{'}\left( d_{\pi-\pi}, DL \right) \{\} \end{aligned} \right. (\boldsymbol{Eq.S}\boldsymbol{1})$$

Then, for further calculation, three assumptions are made: (1) all the correlations shown in **Figure S23** are linear (which is reasonable for a small range of *d_π-π_* and *DL*), (2) samples in **Figure S23**a and b have the same *DL*, and (3) samples in **Figure S23**c and d have the same *d_π-π_*. The assumptions (2) and (3) are supported by the experimental data shown in the main text (**Table S1**).

Based on assumption (1), **Eq. S1** can be reformed in the matrix form shown in **Eq. S2**:

$$\left[ \begin{matrix} \Delta(\Delta R) \\ {\Delta A}^{'} \end{matrix} \right]=\left[ \begin{matrix} \frac{\partial\Delta R}{\partial d_{\pi-\pi}} & \frac{\partial\Delta R}{\partial DL} \\ \frac{\partial A^{'}}{\partial d_{\pi-\pi}} & \frac{\partial A^{'}}{\partial DL} \end{matrix} \right]\left[ \begin{matrix} {\Delta d}_{\pi-\pi} \\ \Delta DL \end{matrix} \right] (\boldsymbol{Eq.S}\boldsymbol{2})$$

By multiplying both sides of **Eq. S2** with the inverse matrix, **Eq. S3** can be derived**.**

$\left[ \begin{matrix} \frac{\partial\Delta R}{\partial d_{\pi-\pi}} & \frac{\partial\Delta R}{\partial DL} \\ \frac{\partial A^{'}}{\partial d_{\pi-\pi}} & \frac{\partial A^{'}}{\partial DL} \end{matrix} \right]^{-1}\left[ \begin{matrix} \Delta(\Delta R) \\ {\Delta A}^{'} \end{matrix} \right]=\left[ \begin{matrix} \frac{\partial\Delta R}{\partial d_{\pi-\pi}} & \frac{\partial\Delta R}{\partial DL} \\ \frac{\partial A^{'}}{\partial d_{\pi-\pi}} & \frac{\partial A^{'}}{\partial DL} \end{matrix} \right]^{-1}\left[ \begin{matrix} \frac{\partial\Delta R}{\partial d_{\pi-\pi}} & \frac{\partial\Delta R}{\partial DL} \\ \frac{\partial A^{'}}{\partial d_{\pi-\pi}} & \frac{\partial A^{'}}{\partial DL} \end{matrix} \right]\left[ \begin{matrix} {\Delta d}_{\pi-\pi} \\ \Delta DL \end{matrix} \right]=\left[ \begin{matrix} {\Delta d}_{\pi-\pi} \\ \Delta DL \end{matrix} \right]$ (***Eq. S3***)

Then, based on the assumptions (2) and (3) mentioned above, the slope of each correlation in **Figure S23** stands for the partial derivative coefficients in **Eq. S3**. Finally, the average of *d_π-π_*, *DL*, $\Delta$*R*, and *A’*, represented by $\bar{d_{\pi-\pi}}$ (3.420 Å), $\bar{DL}$ (29.31%), $\bar{\Delta R}$ (-139.789 and -139.124 cm^-1^ for GIWAXS and XPS samples, respectively), and $\bar{A^{'}}$ (2.154 and 2.618 for GIWAXS and XPS samples, respectively), respectively, are introduced as individual references (**Eq. S4**):

$$\left\{ \begin{aligned} \frac{\partial\Delta R}{\partial d_{\pi-\pi}}=22.944 \{{cm}^{-1}Å^{-1}\} \\ \frac{\partial\Delta R}{\partial DL}=-0.1456 \{{cm}^{-1}\} \\ \frac{\partial A^{'}}{\partial d_{\pi-\pi}}=-16.215 \{Å^{-1}\} \\ \frac{\partial A^{'}}{\partial DL}=-0.0507 \{\} \end{aligned} \right., \left\{ \begin{aligned} \Delta\left( \Delta R \right)=\Delta R-\bar{\Delta R} \{{cm}^{-1}\} \\ {\Delta A}^{'}=A^{'}-\bar{A^{'}} \{\} \\ {\Delta d}_{\pi-\pi}=d_{\pi-\pi}-\bar{d_{\pi-\pi}} \{Å\} \\ \Delta\left( DL \right)=DL-\bar{DL} \{\} \end{aligned} \right. (\boldsymbol{Eq.S}\boldsymbol{4})$$

By substituting the parameters in **Eq. S4**, **Eq. S5** can be obtained.

$\left[ \begin{matrix} 0.0144 & -0.0413 \\ -4.601 & -6.5106 \end{matrix} \right]\left[ \begin{matrix} \Delta R-\bar{\Delta R} \\ A^{'}-\bar{A'} \end{matrix} \right]=\left[ \begin{matrix} d_{\pi-\pi}-\bar{d_{\pi-\pi}} \\ DL-\bar{DL} \end{matrix} \right]$ (***Eq. S5***)

The equation can be expanded into two individual equations.

$$\left\{ \begin{aligned} d_{\pi-\pi}-3.42 =0.0144*\left( \left( \Delta\upsilon_{C_{\beta}-O-C}- {\Delta\upsilon}_{C_{\beta}-O} \right)-\left( -139.789 \right) \right)-0.0413*(\frac{A_{C-O-C}}{A_{C_{\beta}-O}}-2.154) \{Å\} (\boldsymbol{Eq. S}\boldsymbol{5.1}) \\ DL-29.31= -4.60*\left( \left( {\Delta\upsilon}_{C_{\beta}-O-C}- \Delta\upsilon_{C_{\beta}-O} \right)-(-139.124) \right)-6.51*(\frac{A_{C_{\beta}-O-C}}{A_{C_{\beta}-O}}-2.618) \{\%\} (\boldsymbol{Eq. S}\boldsymbol{5.2}) \end{aligned} \right.$$

Finally, we obtained the empirical equations, **Eq. 1 & 2**.

$\left\{ \begin{aligned} d_{\pi-\pi} =0.0144*\left( \Delta\upsilon_{C_{\beta}-O-C}- {\Delta\upsilon}_{C_{\beta}-O} \right)-0.0413*\frac{A_{C-O-C}}{A_{C_{\beta}-O}}+5.5114 \{Å\} (\boldsymbol{Eq. 1}) \\ DL = -4.60*\left( {\Delta\upsilon}_{C_{\beta}-O-C}- \Delta\upsilon_{C_{\beta}-O} \right)-6.51*\frac{A_{C_{\beta}-O-C}}{A_{C_{\beta}-O}}-593.77 \{\%\} (\boldsymbol{Eq. 2}) \end{aligned} \right.$

- **Equilibrium equation fitting of applied Na_2_SO_3_ concentration and PEDOT doping level**

According to the literature, sulfite anion can reduce oxidized EDOT^+^ into neutral EDOT^0^ with the following reversible reaction (***Eq. 3***):

$$2{EDOT}^{+}+{{SO}_{3}}^{2-}+H_{2}O\rightleftharpoons2{EDOT}^{0}+{{SO}_{4}}^{2-}+{2H}^{+} (\boldsymbol{Eq. 3})$$

During the reduction process, Na_2_SO_3_-reduced PEDOT:PSS thin films were post-treated for 15 minutes to ensure the system had sufficient time to reach the equilibrium state. For that, the final concentration of each species in the system can be described as the following equilibrium equation (***Eq. 4***):

$$K_{eq.} = \frac{\left[ {EDOT}^{0} \right]^{2}\cdot\left[ {{SO}_{4}}^{2-} \right]\cdot\left[ H^{+} \right]^{2}}{\left[ {EDOT}^{+} \right]^{2}\cdot[{{SO}_{3}}^{2-}]} \{M^{2}\} (\boldsymbol{Eq. 4})$$

Where K_eq_ is an equilibrium constant only affected by temperature, $\left[ {EDOT}^{0} \right]$, $\left[ {EDOT}^{+} \right]$, $\left[ {{SO}_{4}}^{2-} \right]$, $[{{SO}_{3}}^{2-}]$, and $\left[ H^{+} \right]$ are the concentration of neutral EDOT, oxidized EDOT, sulfate anion, sulfite anion, and proton concentration in the system.

For calculating the concentration of each species, we need the following physical parameters first.

1. **PEDOT doping level (DL)**: the molar ratio of doped EDOT units (EDOT^+^) among all EDOT units, which can be obtained by XPS fitting shown in **Figure S21**.

$$\left[ EDOT \right]=\left[ {EDOT}^{+} \right]+\left[ {EDOT}^{0} \right] \{M\}$$

$$DL= \frac{[{EDOT}^{+}]}{\left[ {EDOT}^{+} \right]+[{EDOT}^{0}]}=\frac{[{EDOT}^{+}]}{\left[ EDOT \right]} \{ \}$$

2. **PSS-to-PEDOT ratio (PPR)**: the molar ratio of sulfonate (styrene sulfonate units, SS^-^, from PSS) to thiophene (EDOT units from PEDOT), which can be obtained from XPS spectra.

$$PPR= \frac{[{SS}^{-}]}{\left[ {EDOT}^{+} \right]+[{EDOT}^{0}]}=\frac{[{SS}^{-}]}{\left[ EDOT \right]} \{ \}$$

PPR is fixed as 1.64 (the average PPR of all Na_2_SO_3_-reduced XPS samples) in the following calculations for convenience.

1. **Molar ratio of EDOT, SS^-^, and Na^+^ ($\boldsymbol{C}_{\boldsymbol{EDOT}}$, $\boldsymbol{C}_{\boldsymbol{SS}^{\boldsymbol{-}}}$, and $\boldsymbol{C}_{\boldsymbol{Na}^{\boldsymbol{+}}}$)**:

$$\left\{ \begin{aligned} C_{EDOT}=\frac{\left[ EDOT \right]}{\left[ EDOT \right]+\left[ {SS}^{-} \right]+[{Na}^{+}]} \{ \} \\ C_{{SS}^{-}}=\frac{\left[ {SS}^{-} \right]}{\left[ EDOT \right]+\left[ {SS}^{-} \right]+[{Na}^{+}]} \{ \} \\ C_{{Na}^{+}}=\frac{[{Na}^{+}]}{\left[ EDOT \right]+\left[ {SS}^{-} \right]+[{Na}^{+}]} \{ \} \end{aligned} \right.$$

EDOT units, styrene sulfonate units, SS^-^, and sodium are assumed to be the only species in pristine PEDOT:PSS. Therefore, we have the first constraint:

$$C_{EDOT}+C_{{SS}^{-}}+C_{{Na}^{+}}=1$$

The sodium styrene sulfonate units are assumed to be fully dissociated, and the charged species have to follow the second constraint below to be a neutral system.

$$\left[ {SS}^{-} \right]=\left[ {EDOT}^{+} \right]+[{Na}^{+}]$$

Using the two constraints and the equations in points 1)-2) mentioned above, the molar concentration of each species can be present by DL and PPR as follows;

1. **Average molecular weight ($\boldsymbol{M}_{\boldsymbol{average}}$)**: the average molecular weight of PEDOT:PSS is calculated as follows:

$$M_{average}= M_{EDOT}\cdot C_{EDOT}+M_{{SS}^{-}}\cdot C_{{SS}^{-}}+M_{{Na}^{+}}\cdot C_{{Na}^{+}}$$

Where $M_{average}$, $M_{{SS}^{-}}$, and $M_{{Na}^{+}}$ are the molecular weight of EDOT unit (140.18 g/mol), styrene sulfonate (SS^-^) units (184.21 g/mol), and sodium (22.99 g/mol), respectively. In here, the EDOT unit molecular weight is 2 Dalton less than EDOT molecular weight because 2 protons per repeating EDOT unit are removed during the polymerization process. By the molecular weight and molar ratio of each species, the average molecular weight is obtained as follows:

$$M_{average}= \frac{140.18+184.21*PPR+22.99*(PPR-DL)}{1+2PPR-DL}\{\frac{g}{mole}\}$$

1. **PEDOT:PSS density ($\boldsymbol{D}_{\boldsymbol{PEDOT:PSS}}$)**: for convenience, the PEDOT:PSS density is assumed to be the same as its dried film density (1.5 g/cm^3^) reported in the literature.^[23]^ Because the swelled film density and the reduced film density are not available, we made two assumptions to simplify the following calculation: 1) The swelling of PEDOT:PSS is ignored ($\boldsymbol{D}_{\boldsymbol{PEDOT:PSS}}$ is fixed as 1.5g/cm^3^), and 2) the molar ratios between species ($C_{EDOT}$, $C_{{SS}^{-}}$, $C_{{Na}^{+}}$) and average molecular weight ($M_{average}$) are considered as fixed values.

Therefore, we obtained the initial molar ratio of each species before the reduction reaction and average molecular weight as follows. Note that even though *DL_0_* is available from XPS measurements (37.6 % as shown in **Table 1** or **Table S1, S3**), it is kept as an unknown to be fitted into the equilibrium equation. This is done to assess the validity of this development by comparing the fitted value with the measured one by XPS):

$$M_{average}= \frac{140.18+184.21*1.64+22.99*(1.64-{DL}_{0})}{1+2*1.64-{DL}_{0}}=\frac{479.988-22.99*{DL}_{0}}{4.28-{DL}_{0}}\{\frac{g}{mole}\}$$

Then, the $\left[ {EDOT}^{0} \right]$ and $\left[ {EDOT}^{+} \right]$ were estimated as follows:

$$\left\{ \begin{aligned} \left[ {EDOT}^{0} \right]=\frac{D_{PEDOT:PSS}}{M_{average}}\cdot C_{EDOT}\cdot\left( 1-DL \right) \\ \left[ {EDOT}^{+} \right]= \frac{D_{PEDOT:PSS}}{M_{average}}\cdot C_{EDOT}\cdot DL \end{aligned} (\boldsymbol{Eq.S}\boldsymbol{6}) \right.$$

By importing the mentioned constants into **Eq. S6**, we obtained the initial state of concentrations before the reaction, shown as follows:

As the reaction produces *t* mole/liter of SO_4_^2-^ to reach an equilibrium state, the equilibrium concentration of each species will be as follows:

$$\left\{ \begin{aligned} \left[ {EDOT}^{0} \right]_{eq.}=\left[ {EDOT}^{0} \right]_{t=0}+2t= \frac{1.5}{479.988-22.99*{DL}_{0}}*{(1-DL}_{t=0})+2t \{M\} \\ \left[ {EDOT}^{+} \right]_{eq.}=\left[ {EDOT}^{+} \right]_{t=0}-2t= \frac{1.5}{479.988-22.99*{DL}_{0}}*{DL}_{t=0}-2t \{M\} \\ \left[ {{SO}_{4}}^{2-} \right]_{eq.}=t \{M\} \\ {[{{SO}_{3}}^{2-}]}_{eq}={[{{SO}_{3}}^{2-}]}_{t=0}= 0, 0.01, 0.05, 0.1, 0.5, or 1 \{M\} \\ \left[ H^{+} \right]_{eq.}={10}^{-7}+2t \approx2t \{M\} \end{aligned} \right.$$

First, $\left[ {EDOT}^{+} \right]_{t=0}$ and $\left[ {EDOT}^{0} \right]_{t=0}$ have been replaced by **Eq. S7.** Because the volume of Na_2_SO_3_ solution is much larger than that of PEDOT:PSS film, the bath is considered a reservoir of SO_3_^2-^ with an unaltered concentration. Then, if Na_2_SO_3_ is assumed to be fully dissociated, the experimental concentration of Na_2_SO_3_ is ${[{{SO}_{3}}^{2-}]}_{t=0}=$ ${[{{SO}_{3}}^{2-}]}_{eq}$. Also, since the reacted SO_4_^2-^ should be much more than 10^-7^ mole/liter, the $\left[ H^{+} \right]_{eq}$ is assumed as 2*t*.

$$t=\frac{\frac{1.5}{479.988-22.99*{DL}_{0}}*{DL}_{0}-\left[ {EDOT}^{+} \right]_{eq.}}{2}$$

Finally, the equilibrium equation **Eq. 4** can be rewritten as a function of $\left[ {EDOT}^{+} \right]_{eq.}$ and $[{{SO}_{3}}^{2-}]$ (both known quantities), shown in the following:

$$K_{eq.}= \frac{{{(\frac{1.5}{479.988-22.99*{DL}_{0}}-\left[ {EDOT}^{+} \right]}_{eq.})}^{2}\cdot(\frac{{\frac{1.5}{479.988-22.99*{DL}_{0}}\cdot{DL}_{0}-\left[ {EDOT}^{+} \right]}_{eq.}}{2})\cdot{({\frac{1.5}{479.988-22.99*{DL}_{0}}\cdot{DL}_{0}-\left[ {EDOT}^{+} \right]}_{eq.})}^{2}}{{\left[ {EDOT}^{+} \right]_{eq}}^{2}\cdot\left[ {{SO}_{3}}^{2-} \right]}$$

Then, one can re-write the equation into the following form for fitting (**Figure S24**),

$$\left[ {{SO}_{3}}^{2-} \right]=\frac{1}{K_{eq.}} \frac{{{(\frac{1.5}{479.988-22.99*{DL}_{0}}-\left[ {EDOT}^{+} \right]}_{eq.})}^{2}\cdot{({\frac{1.5}{479.988-22.99*{DL}_{0}}\cdot{DL}_{0}-\left[ {EDOT}^{+} \right]}_{eq.})}^{3}}{2*{\left[ {EDOT}^{+} \right]_{eq}}^{2}} (\boldsymbol{Eq.S}\boldsymbol{8})$$

$\left[ {{SO}_{3}}^{2-} \right]$ is the sodium sulfite concentration applied for post-treatment, and $\left[ {EDOT}^{+} \right]_{eq.}$ can be obtained from XPS measured *DL* with **Eq. S6**. By fitting the equation *K_eq._* and *DL_0_* result 0.2647 M^2^ and 38.77%, respectively. Conveniently, the value obtained for *DL_0_* is in agreement with the one measured in XPS for non-reduced PEDOT:PSS (37.6 % as shown in **Table 1** or **Table S1, S3**), confirming the validity of the equilibrium equation derivation and fitting.


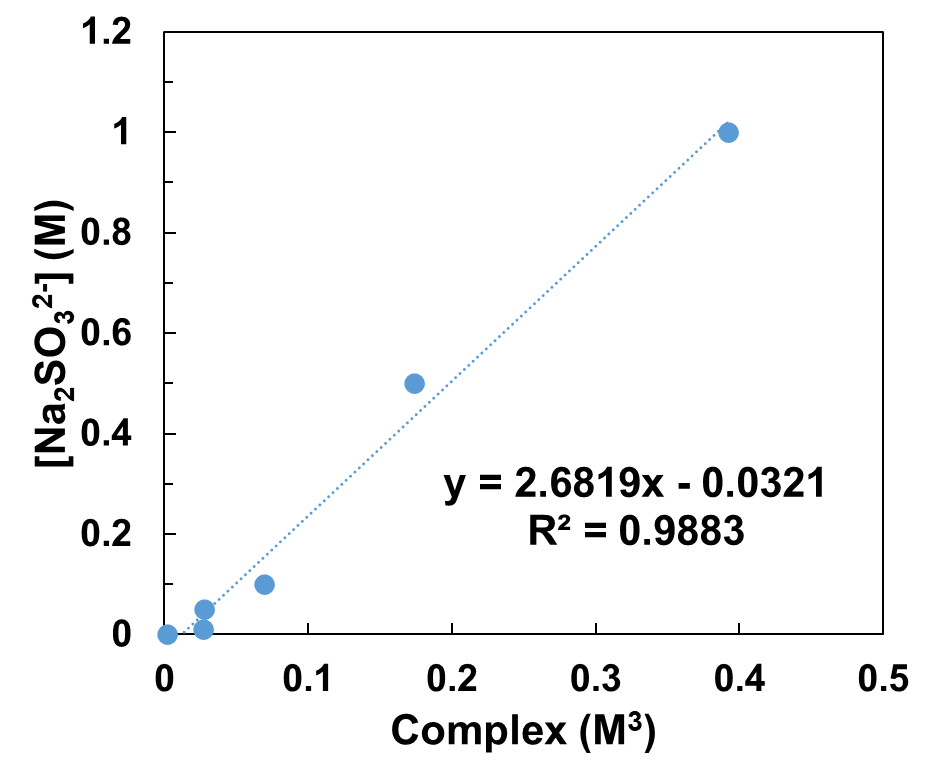


**Figure S24.** [Na_2_SO_3_^2-^] versus the complex right side of **Eq. S8** for *K_eq._* and *DL_0_* fitting.

- **Estimation of carrier mobility of PEDOT:PSS samples**

The oxidized EDOT concentration $\left[ {EDOT}^{+} \right]$ is equivalent to carrier concentration. Therefore, the carrier concentration $N_{h^{+}}$ can be estimated from **Eq. S6** as follows,

$N_{h^{+}}=\left[ {EDOT}^{+} \right]\cdot{10}^{-3}{\cdot N}_{A}=\frac{D_{PEDOT:PSS}}{M_{average}}\cdot C_{EDOT}\cdot DL\cdot N_{A}=1.92\cdot DL\cdot{10}^{21} \{\frac{1}{{cm}^{3}}\}$ (**Eq. S9**)

Where *N_A_* is the Avogadro number needed to pass from moles of charges to absolute number of charges, *PPR* is obtained by fitting XPS results as described in the Experimental Section and shown in **Figure S20**, and *DL* can be extracted from XPS or the proposed **Eq. 2**. The obtained carrier concentration $N_{h^{+}}$ ranges from 4*10^20^ to 8*10^20^ /cm^3^, depending on the post-treatment condition. These estimated values of $N_{h^{+}}$ are close to the reported numbers.^[24]^

Then the carrier mobility can be further calculated from the measured electrical conductivity and Drude’s model:

$\mu_{h^{+}}=\frac{\sigma}{e\cdot N_{h^{+}}}=\frac{\sigma}{e\cdot1.92\cdot DL\cdot{10}^{21}} \{\frac{{cm}^{2}}{V\cdot s}\}$ (**Eq. S10**)

Where $e$ is the elementary charge constant.

- **References**

[1] P. O. Osazuwa, C. Y. Lo, X. Feng, A. Nolin, C. Dhong, L. V. Kayser, "Surface Functionalization with (3-Glycidyloxypropyl)trimethoxysilane (GOPS) as an Alternative to Blending for Enhancing the Aqueous Stability and Electronic Performance of PEDOT:PSS Thin Films," *ACS Appl Mater Interfaces* 15 no.47 (2023): 54711, https://doi.org/10.1021/acsami.3c09452

[2] N. Saxena, J. Keilhofer, A. K. Maurya, G. Fortunato, J. Overbeck, P. Müller-Buschbaum, "Facile Optimization of Thermoelectric Properties in PEDOT:PSS Thin Films through Acido-Base and Redox Dedoping Using Readily Available Salts," *ACS Appl Energy Mater* 1 no.2 (2018): 336, https://doi.org/10.1021/acsaem.7b00334

[3] Y. Tian, I. Florenciano, H. Xia, Q. Li, H. E. Baysal, D. Zhu, et al., "Facile Fabrication of Flexible and High-Performing Thermoelectrics by Direct Laser Printing on Plastic Foil," *Advanced Materials* 36 no.15 (2024), https://doi.org/10.1002/adma.202307945

[4] B. Zhang, T.-Y. Yu, A. Meulemans, V. Naenen, M. Rosenthal, D. Escudero, et al., "Brush and Dry: A Posttreatment-Free Simple Process to Pattern PEDOT:PSS Films with Enhanced Thermoelectric Performance via Shear Flow-Induced Morphology Tuning," *ACS Appl Mater Interfaces* (2025), https://doi.org/10.1021/acsami.5c00803

[5] H. E. Baysal, T. Y. Yu, V. Naenen, S. De Smedt, D. Hiz, B. Zhang, et al., "Omnidirectional 3D Printing of PEDOT: PSS Aerogels with Tunable Electromechanical Performance: A Playground for Unconventional Stretchable Interconnects and Thermoelectrics," *Advanced Science* (2025), https://doi.org/10.1002/advs.202412491

[6] S. Dongmin Kang, G. Jeffrey Snyder, "Charge-transport model for conducting polymers," *Nat Mater* 16 no.2 (2017): 252, https://doi.org/10.1038/nmat4784

[7] S. A. Gregory, R. Hanus, A. Atassi, J. M. Rinehart, J. P. Wooding, A. K. Menon, et al., "Quantifying charge carrier localization in chemically doped semiconducting polymers," *Nat Mater* 20 no.10 (2021): 1414, https://doi.org/10.1038/s41563-021-01008-0

[8] M. Kong, M. Garriga, J. S. Reparaz, M. I. Alonso, "Advanced Optical Characterization of PEDOT:PSS by Combining Spectroscopic Ellipsometry and Raman Scattering," *ACS Omega* 7 no.43 (2022): 39429, https://doi.org/10.1021/acsomega.2c05945

[9] E. L. Lee, I. E. Wachs, "In situ Raman spectroscopy of SiO2-supported transition metal oxide catalysts: An isotopic 18O-16O exchange study," *Journal of Physical Chemistry C* 112 no.16 (2008): 6487, https://doi.org/10.1021/jp076485w

[10] K. Brunner, G. Abstreiter, B. O. Kolbesen, H. W. Meul, “Strain at Si SiO2 interfaces studied by Micron-Raman spectroscopy”, *Appl Surf Sci* 39 no. 1-4 (1989): 116-126, https://doi.org/10.1016/0169-4332(89)90424-8

[11] Z. Jiang, " GIXSGUI: a MATLAB toolbox for grazing-incidence X-ray scattering data visualization and reduction, and indexing of buried three-dimensional periodic nanostructured films," *J Appl Crystallogr* 48 no.3 (2015): 917, https://doi.org/10.1107/S1600576715004434

[12] L. Taussig, M. Ghasemi, S. Han, A. L. Kwansa, R. Li, S. T. Keene, et al., "Electrostatic self-assembly yields a structurally stabilized PEDOT:PSS with efficient mixed transport and high-performance OECTs," *Matter* 7 no.3 (2024): 1071, https://doi.org/10.1016/j.matt.2023.12.021

[13] S. Marina, E. Gutierrez-Fernandez, J. Gutierrez, M. Gobbi, N. Ramos, E. Solano, et al., "Semi-paracrystallinity in semi-conducting polymers," *Mater Horiz* 9 no.4 (2022): 1196, https://doi.org/10.1039/d1mh01349a

[14] J. Rivnay, R. Noriega, R. J. Kline, A. Salleo, M. F. Toney, "Quantitative analysis of lattice disorder and crystallite size in organic semiconductor thin films," *Phys Rev B Condens Matter Mater Phys* 84 no.4 (2011), https://doi.org/10.1103/PhysRevB.84.045203

[15] T. Liu, L. Sun, X. Dong, Y. Jiang, W. Wang, C. Xie, et al., "Low-Work-Function PEDOT Formula as a Stable Interlayer and Cathode for Organic Solar Cells," *Adv Funct Mater* 31 no.51 (2021), https://doi.org/10.1002/adfm.202107250

[16] S. Garreau, G. Louarn, J. P. Buisson, G. Froyer, S. Lefrant, "In situ spectroelectrochemical Raman studies of poly(3,4-ethylenedioxythiophene) (PEDT)," *Macromolecules* 32 no.20 (1999): 6807, https://doi.org/10.1021/ma9905674

[17] O. Bubnova, Z. U. Khan, A. Malti, S. Braun, M. Fahlman, M. Berggren, et al., "Optimization of the thermoelectric figure of merit in the conducting polymer poly(3,4-ethylenedioxythiophene)," *Nat Mater* 10 no.6 (2011): 429, https://doi.org/10.1038/nmat3012

[18] N. Massonnet, A. Carella, O. Jaudouin, P. Rannou, G. Laval, C. Celle, et al., "Improvement of the Seebeck coefficient of PEDOT:PSS by chemical reduction combined with a novel method for its transfer using free-standing thin films," *J Mater Chem C Mater* 2 no.7 (2014): 1278, https://doi.org/10.1039/c3tc31674b

[19] I. Alessandri, F. Torricelli, B. Cerea, M. Speziani, P. Romele, Z. M. Kovacs-Vajna, et al., "Why PEDOT:PSS Should Not Be Used for Raman Sensing of Redox States (and How It Could Be)," *ACS Appl Mater Interfaces* 14 no.50 (2022): 56363, https://doi.org/10.1021/acsami.2c17147

[20] J. Peng, Q. Lin, T. Földes, H. H. Jeong, Y. Xiong, C. Pitsalidis, et al., "In-Situ Spectro-Electrochemistry of Conductive Polymers Using Plasmonics to Reveal Doping Mechanisms," *ACS Nano* 16 no.12 (2022): 21120, https://doi.org/10.1021/acsnano.2c09081

[21] J. Ouyang, Q. Xu, C. W. Chu, Y. Yang, G. Li, J. Shinar, "On the mechanism of conductivity enhancement in poly(3,4- ethylenedioxythiophene):poly(styrene sulfonate) film through solvent treatment," *Polymer (Guildf)* 45 no.25 (2004): 8443, https://doi.org/10.1016/j.polymer.2004.10.001

[22] S. Spange, N. Weiß, T. G. Mayerhöfer, "The Global Polarity of Alcoholic Solvents and Water – Importance of the Collectively Acting Factors Density, Refractive Index and Hydrogen Bonding Forces," *ChemistryOpen* 11 no.10 (2022), https://doi.org/10.1002/open.202200140

[23] Z. Guo, T. Sato, Y. Han, N. Takamura, R. Ikeda, T. Miyamoto, et al., "Band transport evidence in PEDOT:PSS films using broadband optical spectroscopy from terahertz to ultraviolet region," *Commun Mater* 5 no.1 (2024), https://doi.org/10.1038/s43246-024-00451-1

[24] S. T. Keene, W. Michaels, A. Melianas, T. J. Quill, E. J. Fuller, A. Giovannitti, et al., "Efficient Electronic Tunneling Governs Transport in Conducting Polymer-Insulator Blends," *J Am Chem Soc* 144 no.23 (2022): 10368, https://doi.org/10.1021/jacs.2c02139
